# Counterion Structure Tunes Cetyltrimethylammonium Association in Water: Thermodynamics and Weak-Contact Networks

**DOI:** 10.1021/acs.langmuir.6c04226

**Published:** 2026-10-01

**Authors:** Jakub Brzeski, Sylwia Freza, Dariusz Wyrzykowski

**Affiliations:** Faculty of Chemistry, 49646 University of Gdańsk, Wita Stwosza 63, Gdańsk 80-308, Poland

## Abstract

The role of counterion structure in the stability and interaction mechanisms of cetyltrimethylammonium ion pairs was investigated by employing isothermal titration calorimetry (ITC), molecular dynamics simulations, and quantum-chemical calculations. Alkyl sulfate anions with increasing chain length and tetraphenylborate were used to distinguish between the effects of electrostatic and hydrophobic interactions as well as static anion polarizability. The ITC measurements showed stronger binding for longer alkyl sulfate chains. Namely, the corresponding Gibbs free energy spanned from −6.78 kcal/mol for octyl sulfate to −8.82 kcal/mol for dodecyl sulfate. Tetraphenylborate showed similar binding strength with ΔG equal to −8.75 kcal/mol. Molecular dynamics simulations were applied to study the conformational space of CTA^+^/anion associates in water. Afterward, the representative structures were selected for quantum-chemical characterization. The energy decomposition analysis showed that although electrostatic interaction dominates, dispersion becomes more important for both longer alkyl sulfates and tetraphenylborate-based systems. Quantum theory of atoms in molecules (QTAIM) analysis showed that stabilization is not determined by a single strong contact. Instead, longer alkyl chains form more weak hydrophobic contacts. The results explain how the structure of the studied counterions affects the CTA^+^ association through a balance of electrostatic interactions and multiple hydrophobic contacts.

## Graphical abstract

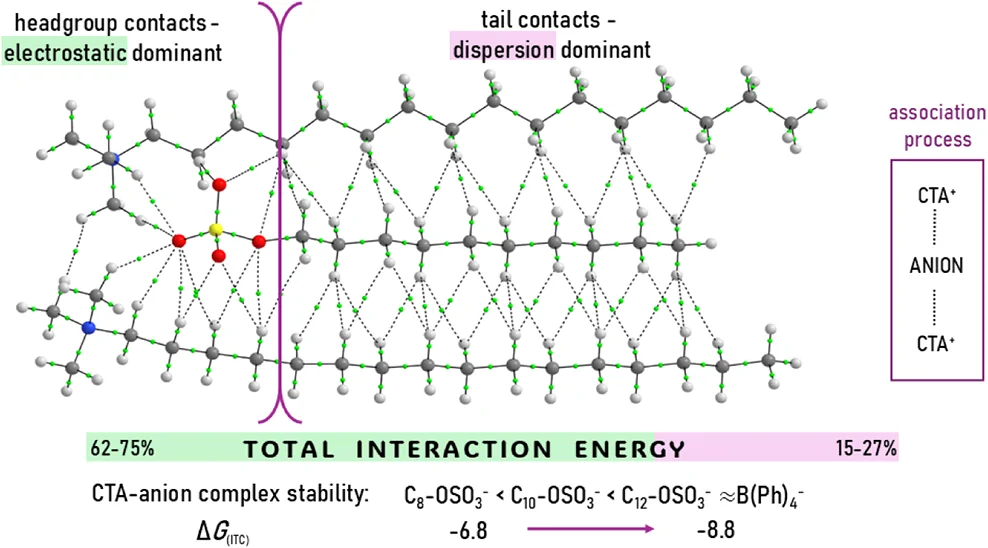

## Introduction

The amphiphilic nature of species characterized by a hydrophobic tail and a hydrophilic headgroup, gives rise to key physicochemical properties such as self-aggregation, surface activity (including interfacial adsorption), reduction of surface and interfacial tension, and self-assembly in aqueous solution.^1^ Among these species, quaternary ammonium surfactants and particularly those containing a quaternary ammonium head group, are one of the most extensively studied and utilized groups. Quaternary ammonium surfactants are a central class of cationic amphiphiles. Their simple headgroup architecture allows for an analysis of counterion binding, micellization, and ion-specific effects.^2,3^ Besides being used as surfactants, they have also received well-deserved attention in last decades mostly due to their pharmaceutical properties.^4,5^ Within this family, alkyltrimethylammonium compounds stand out as a key subclass due to their simple architecture, which makes them ideal models for investigating micelle growth and counterion effects.^6^ A prominent example is the cetyltrimethylammonium cation, typically paired with bromide to form cetyltrimethylammonium bromide (CTAB). CTAB is widely employed in studies colloidal organization, interfacial processes, and molecular interactions.^7–11^ Moreover, it is also a reliable reagent for both basic laboratory practices and advanced life science research.^12^

The presence of counterions is essential to the behavior of cationic surfactants. In most cases, the role of the anion is played by inorganic species such as chloride, bromide, iodide or nitrate.^13–15^ Nonetheless, more hydrophobic organic anions such as acetate, tosylate or benzoate are also widely used.^16–19^ Regardless of the chemical nature of the counterion in question, their indisputable role is to balance the charge and participate in the self-assembly process. By partially screening the positive charge they neutralize the repulsion between adjacent cationic surfactant headgroups, thus affecting critical micelle concentration as well as its shape, size and adsorption properties.^20^ It has also been shown that counterions impact the overall type of micelle formed—spherical, wormlike, vesicular, coacervate, or other.^15,17^ An important consideration in this context is ion specificity, as not all anions bind to cationic surfactants with equal affinity.^21^ Counterion binding typically increases with the polarizability and hydrophobicity of the anion.^22–24^ Consequently, hydrophobic organic anions tend to bind more strongly than simple halides, inserting into the interfacial region and promoting tighter packing.^24^ The counterions were also found to affect the phase behavior and solubility, including the Krafft temperature,^25^ i.e., a narrow temperature range above which the surfactant’s solubility is equal to the critical micelle concentration.^26^ To summarize, counterions control cationic-surfactant behavior through three coupled effects: (i) electrostatic screening, (ii) specific binding/ion pairing and (iii) interfacial dehydration or hydrophobic insertion.^27,28^

Although the interactions between simple inorganic counterions and ionic surfactants have been relatively well studied, the role of organic counterions, particularly those with a tunable balance of hydrophobic and hydrophilic properties, has received relatively little attention, often being considered only in specific contexts.^29–32^ This point is relevant because variations in the shape, size, and hydrophobicity of organic counterions can alter surfactant self-assembly, interfacial properties, and phase behavior. Therefore, a thorough understanding of how the molecular structure of an anion governs its binding to a cationic surfactant is essential for the design of surfactant-based systems. Motivated by this need, in this paper the hexadecyltrimethylammonium cation (CTA^+^) has been selected as a well-defined model system, for which the formation of ion pairs with a series of structurally diverse anions is investigated. Specifically, the alkyl sulfate anions (C_n_–OSO_3_^–^) with varying hydrophobic chain lengths:^33^ octyl sulfate (C_8_–OSO_3_^–^), decyl sulfate (C_10_–OSO_3_^–^) and dodecyl sulfate (C_12_–OSO_3_^–^), each of which possesses a linear, flexible hydrocarbon chain are explored. For comparison, also tetraphenylborate (B­(Ph)_4_^–^), which features four phenyl groups arranged tetrahedrally around a central boron atom to form a rigid, bulky, highly hydrophobic anion is included. Even though isolated ion pairs do not fully reproduce the complexity of a micelle, they constitute a molecular model of the counterion binding motifs present at micellar interfacial regions.

Intermolecular interactions are known to influence molecular properties and electronic structure, and even individually weak contacts can become important through accumulation.^34–36^ To establish a physicochemical background to these interactions within studied systems, and to correlate the structural features of the anions with their binding modes, a combined experimental and quantum chemical approach was undertaken. Although the effects of counterions in cationic surfactant systems are widely recognized, the molecular basis of anion-dependent stability in CTA^+^-based ion pairs has not been fully elucidated. To the best of authors’ knowledge, no study has yet systematically linked microscopic interaction mechanisms, such as electrostatic, induction and dispersion forces, to macroscopic behavior across a matched series of CTA^+^ ion pairs.

In this work, the influence of anion identity on CTA^+^ association was investigated using ITC, molecular dynamics, and quantum chemistry methods. The obtained results are discussed in terms of their impact on the stability of the associations, thereby providing a rationale for counterion-specific effects in cationic surfactant systems. The molecular level research presented in this paper provides a basis for future research on higher order self-assemblies.

## Materials and Methods

### Experimental Methods

#### Materials

Hexadecyltrimethylammonium bromide (CTAB, ≥99%, CAS: 57-09-0), sodium dodecyl sulfate (C_12_–OSO_3_Na, CAS: 151-21-3, ≥99%), sodium decyl sulfate (C_10_–OSO_3_Na, CAS: 142-87-0, 98%), sodium octyl sulfate (C_8_–OSO_3_Na, CAS: 142-31-4, ≥95%) and sodium tetraphenylborate (Na­[B­(Ph)_4_], ≥99.5%, CAS: 143-66-8) were purchased from Sigma-Aldrich (Poland) and used without further purification. Double-distilled water (purified by Hydrolab-system) with conductivity not exceeding 0.18 μS cm^–1^ was used for preparations of aqueous solutions.

#### Isothermal Titration Calorimetry

All ITC experiments were performed at 298.15 K using an AutoITC isothermal titration calorimeter (MicroCal Inc., GE Healthcare, Northampton, USA). The thermodynamic parameters, namely the binding constant (*K*_ITC_) and the enthalpy change (Δ*H*_(ITC)_) and the stoichiometry (*N*), were derived directly from the ITC titration data. The details of the measuring devices and experimental setup were described previously.^37^ The experimental isotherms were fitted using a nonlinear least-squares regression to a model assuming a single set of identical and independent binding sites.^38^ This model provided the best fit between the calculated and experimental data for all investigated systems (CTA^+^ with C8-, C10-, C12-alkyl sulfates and tetraphenylborate ion). The standard thermodynamic relationships: Δ*G*_ITC_ = −R*T* ln *K*_ITC_ = Δ*H*_ITC_ – *T*Δ*S*_ITC_ were used to calculate the free energy of binding, Δ*G*_ITC_, and the entropy change, Δ*S*_ITC_. Uncertainties (Δ*G*_ITC_, TΔ*S*_ITC_) were propagated using the standard error propagation formulas. The reagents were dissolved directly in water. To ensure that the measured thermodynamic parameters reflect genuine monomer–monomer ion-pair formation rather than aggregate or micelle-associated processes, all ITC experiments were performed at total surfactant concentrations well below the critical micelle concentrations (CMCs) of the individual components and below the onset concentration for catanionic aggregate formation. Each experiment began with an initial sample cell containing approximately 0.05 mM CTAB solution. Into this cell, 29 injections were made: the first injection was 2 μL, and the remaining 28 injections were 10.02 μL each, with an injection duration of 20 s and injection interval 210 s. The titrant solutions were either 1 mM surfactant (C_8_–OSO_3_Na, C_10_–OSO_3_Na or C_12_–OSO_3_Na) or 1 mM Na­[B­(Ph)_4_]. For each titration, a corresponding blank experiment was performed by injecting the same titrant into a cell containing only water. The resulting heat of dilution was subtracted from the main titration data. Reagent concentrations were selected to remain below their respective critical micelle concentrations.^39^

### Computational Methods

#### Molecular Dynamics Simulations

The force-field parameters of CTA^+^ as well as C_8_–OSO_3_^–^, C_10_–OSO_3_^–^, C_12_–OSO_3_^–^ and B­(Ph)_4_^–^ based on HF/6-31G* electrostatic potential calculations carried out in Gaussian (Revision C.02) were prepared using GAFF2 force field.^40^ The partial atomic charges were calculated using the Restrained Electrostatic Potential (RESP) method^41^ and AmberTools 25,^42^ as implemented in Amber24.^43^ Atomic types as well as bonded/nonbonded parameters for organic ions were assigned in the GAFF2 standard. The missing parameters were checked and generated with parmchk2 program, while the consistency of the obtained topology and parameter files was confirmed with tleap. For B­(Ph)_4_^–^ RESP charges and GAFF2 aromatic atom types were used for the phenyl rings. Due to the fact that GAFF2 does not provide a complete automatic description of the tetracoordinate boron center, the missing boron-related bonded and nonbonded parameters were created as a GAFF2-compatible parameter set in this work. This was performed by using the QM-optimized B­(Ph)_4_^–^ geometry and standard tetrahedral/aromatic geometry constraints as reference. This approach is in agreement with similar previous simulations of tetraphenylborate and related aromatic boron compounds.^44,45^ The simulated systems were constructed as explicit salt mixtures corresponding to the experimental stoichiometry. Namely, for the CTA:anion 1:1 ratio a system containing: 32 CTA^+^, 32 Br^–^, 32 A^–^ and 32 Na^+^ was used. Whereas, for the 2:1 stoichiometry, the following composition was assumed: 32 CTA^+^, 32 Br^–^, 16 A^–^ and 16 Na^+^ was used. Additionally, a CTAB-only reference system containing 32 CTA^+^ and 32 Br^–^ ions was simulated to separate the intrinsic CTA^+^/Br^–^ aggregation behavior from the effects induced by the added organic anions. Periodic simulation boxes were created by solvating ionic configurations formed in Packmol^46^ with TIP3P^47^ water model using a 6 Å solvent buffer. First, the systems were minimized with restraints on the organic solutes. This was followed by an unrestrained minimization of the full system. The temperature was then increased from 50 to 300 K over 250 ps with restraints on the organic solutes, followed by a 1 ns NPT density relaxation step with weaker restraints. All restraints were removed for the final 5 ns NPT equilibration. Afterward, the production MD was carried out under periodic boundary conditions for 400 ns per replica. These were performed as four consecutive 100 ns segments propagated through restart files. Production simulations were unrestrained and used a 2 fs timestep, coordinate saving every 100 ps, and restart propagation between segments. Temperature, relative cell volume, and mean-centered potential energy remained stable after the initial relaxation; therefore, CTA-anion complexes were extracted only from the final 200 ns of each trajectory (vide Figures S1–S3). Atom-resolved contact maps were calculated from the final 200 ns of each 400 ns production trajectory, using one frame every 1 ns. For each stoichiometry, three independently initialized simulations were analyzed separately. Full details of the map construction are provided in the Supporting Information. After the production simulations have finished a total of 1914 structures of 1:1 and 2:1 CTA:A were extracted from the simulations. The final dataset contained 240 structures for C8 1:1, C10 1:1, C12 1:1, C12 2:1, and B­(Ph)_4_^–^ 1:1, and 238 structures for C8 2:1, C10 2:1, and B­(Ph)_4_^–^ 2:1. The complete extraction procedure is presented in Supporting Information.

#### Quantum Chemical Calculations

The extracted MD structures were optimized using g-XTB^48^ (version 2.0.1) method parametrized to approximate ωB97M-V^49^/def2-TZVPPD level calculations. Solvent effects were approximated using the domain decomposition conductor-like screening model (ddCOSMO).^50^ After the optimizations, all isomers of each system were ranked with regard to energy. The 15 lowest energy for each stoichiometry were then subjected to DFT characterization.

When it comes to the equilibrium structures of the isolated species i.e., CTA^+^, C_8_–OSO_3_^–^, C_10_–OSO_3_^–^, C_12_–OSO_3_^–^ and B­(Ph)_4_^–^, the DFT calculations were preceded by comprehensive conformational search within CREST (version 3.0.2) software^51^ using the medadynamics workflow^52^ at the GFN2-XTB^53^ level of theory. Solvent effects were approximated implicitly by using the analytical linearized Poisson-Boltzmann (ALPB) water solvation model.^54^ This search has led to 71443, 1887, 6175, 10967, and 3 conformers for CTA^+^, C_8_–OSO_3_^–^, C_10_–OSO_3_^–^, C_12_–OSO_3_^–^ and B­(Ph)_4_^–^, respectively. The three conformers of B­(Ph)_4_^–^ and 15 lowest of alkyl-chain amphiphiles were then subjected to DFT analysis. The optimized geometries of CTA^+^, C_8_–OSO_3_^–^, C_10_–OSO_3_^–^, C_12_–OSO_3_^–^, B­(Ph)_4_^–^as well as those of the corresponding 1:1 and 2:1 CTA^+^:anion complexes were then optimized using *ω*B97X-3c composite method^55^ and the conductor-like polarizable continuum model (CPCM)^56^ solvation. The vibrational analysis was carried out at the very same level in order to assure that all found stationary points are true minima on the potential energy surface. Thus, obtained thermochemical data were further processed using GoodVibes software (version 4.3.0)^57^ to obtain quasi-harmonically corrected Gibbs free energies and to perform the final ranking of the isomers. The modified rigid-rotor harmonic-oscillator entropy correction was applied^58^ using a frequency cutoff of 100 cm^–1^, together with the quasi-harmonic enthalpy treatment^59^ at 298.15 K and 1.0 mol/L. The final selection of the lowest-energy isomers was based on the resulting quasi-harmonically corrected Gibbs free energies. These corrections are important because the studied ion-pair complexes are flexible and contain many low frequency motions. If these motions are treated only harmonically, the vibrational entropy may be overestimated, which can affect the relative Gibbs free energies and the isomer ranking. Nevertheless, one has to bear in mind that quasi-harmonic treatment is an approximate description of the entropy of highly flexible complexes. This is due to the fact, that low-frequency modes may exhibit significant anharmonicity. Moreover, a single-minimum treatment does not fully account for the multiple accessible conformations. Therefore, the calculated Gibbs free energies should be treated with caution.

The Cartesian coordinates of the equilibrium structures of the lowest Gibbs free energy isomer of the components as well as five lowest energy isomers of the complexes are collected in Table S1. Subsequent analyses were performed using the lowest Gibbs free energy isomer of each system. All DFT calculations were carried out using ORCA software (version 6.1.1).^60^ The Gibbs free energies of association (Δ*G_assoc_*) were calculated according to the following equation:

1 $$\Delta G_{assoc}=G_{solv}^{comp}-G_{solv}^{anion}-nG_{solv}^{CTA^{+}}$$

where “*solv*” indicates solvated phase, “*comp*” superscript denotes the complex, “*anion*” the free anion, and “*n*” the number of CTA molecules in a system.

In the context of surfactant-counterion association, polarizability is an important descriptor, as it is related to the induction and dispersion contributions to the total interaction energy. As such, it may affect the stabilization of studied ion pairs and, indirectly counterion binding at micellar interfaces. Static polarizabilities were calculated for the gas-phase structures from the corresponding dipole polarizability tensors obtained from ωB97X-D4 ^61^/def2-TZVPD single-point calculations employing CPCM solvation. The reported isotropic polarizability values, *α_iso_* correspond to one third of the trace of the polarizability tensor:

2 $$\alpha_{\mathrm{iso}}=\frac{\alpha_{xx}+\alpha_{yy}+\alpha_{zz}}{3}$$

The polarizability anisotropy was evaluated as the invariant γ of the polarizability tensor. Which for a symmetric tensor in its principal-axis frame equals:^62^

3 $$\gamma =\sqrt{\frac{{\left(\alpha_{1}-\alpha_{2}\right)}^{2}+{\left(\alpha_{2}-\alpha_{3}\right)}^{2}+{\left(\alpha_{3}-\alpha_{1}\right)}^{2}}{2}}$$

For the 1:1 complexes, Symmetry-Adapted Perturbation Theory (SAPT2)^63^ calculations were carried out with aug-cc-pVDZ Dunning-type basis set^64^ of double-ζ quality in order to quantify the strength and nature of the associations formed. The calculations employed density-fitted self-consistent field (DF-SCF) with the aug-cc-pVDZ-JKFIT auxiliary basis set.^65^ For both 1:1 and 2:1 CTA-guest stoichiometries the interaction energies were calculated using the many-body procedure at the ωB97X-V/aug-cc-pVDZ level with Valiron-Mayer function counterpoise correction (VMFC).^66^ All interaction energy calculations were carried out using PSI4 software (1.9.1 release).^67^ These calculations were performed in order to assess the overall stabilization of the larger aggregates and to enable comparison with the corresponding 1:1 systems.

The quantitative analysis of molecular surface as implemented in Multiwfn package (version 2026.3.11) was performed based on the ωB97X-D4/def2-TZVPD wavefunctions for all complexes.^68,69^ The Quantum Theory of Atoms in Molecules (QTAIM)^70^ analysis was performed in order to get a better understanding of the intermolecular interactions stabilizing CTA-counterion systems. All QTAIM calculations were carried out using AIMALL (Version 19.10.12) computational package.^71^ The molecular graphs of the optimized complexes were inspected to locate intermolecular bond paths and bond critical points (BCPs) within the studied complexes. For the representative contacts stabilizing the studied systems, the values of the Laplacian of the electron density (∇^2^ρ­(r)), the absolute ratio of the potential energy density to the kinetic energy density at the bond critical point $\left(\frac{\left|V_{BCP}\right|}{G_{BCP}}\right)$ , as well as the corresponding interaction energies (*E_I_*) estimated using the Espinosa formula are reported.^72^

## Results and Discussion

### Experimental Characterization of CTA-Anion Association

#### CTAB/SXS Interactions

The interactions between the hexadecyltrimethylammonium cation (CTA^+^) and three alkyl sulfate anions (C_n_–OSO_3_^–^) with varying hydrophobic chain lengths, namely: octyl sulfate (C_8_–OSO_3_^–^), decyl sulfate (C_10_–OSO_3_^–^) and dodecyl sulfate (C_12_–OSO_3_^–^), in aqueous solutions, were successfully investigated using the ITC technique. Representative ITC binding isotherms are shown in Figure 1, and the corresponding thermodynamic parameters are summarized in Table 1.

**Figure 1:**
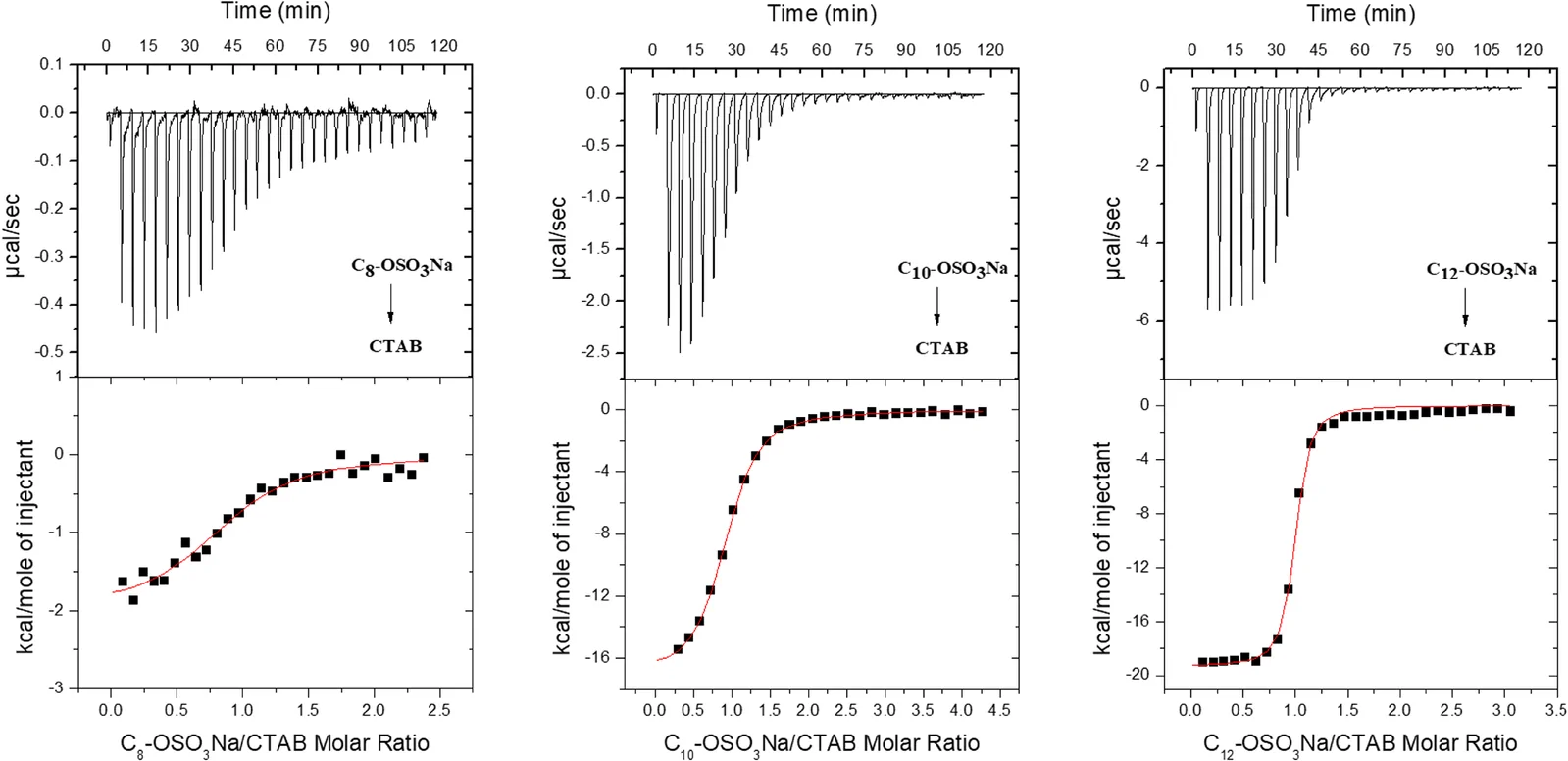
Calorimetric titration isotherms of binding interactions of hexadecyltrimethylammonium cation (CTA^+^) with alkyl sulfate anions (C_n_–OSO_3_^–^; n = 8, 10 and 12) in water at 298.15 K.

**Table 1:** Thermodynamic Parameters of Binding Interactions of CTA^+^ with Alkyl Sulfate Anions (C_n_–OSO_3_^–^) in Water at 298.15 K (Standard Deviation Values in Parentheses)

Parameter	C_8_–OSO_3_^–^	C_10_–OSO_3_^–^	C_12_–OSO_3_^–^
*N = [*C_n_–OSO_3_^–^ *]/[CTA* ^*+*^ *]* *(stoichiometry)*	0.90 (±0.02)	0.91 (±0.05)	0.95 (±0.02)
log*K*_(ITC)_	4.97 (±0.11)	5.56 (±0.02)	6.46 (±0.07)
Δ*G*_(ITC)_ [kcal/mol]	–6.78 (±0.15)	–7.58 (±0.02)	–8.82 (±0.09)
Δ*H*_(ITC)_ [kcal/mol]	–2.01 (±0.12)	–17.17 (±0.12)	–19.31 (±0.19)
*T*Δ*S*_(ITC)_ [kcal/mol]	4.77	–9.59	–10.49

It has been found that the strength of the interactions increases markedly with the hydrophobic chain length. This trend is reflected in the change in Gibbs free energy (Δ*G*), which becomes more negative by approximately −0.4 to −0.6 kcal/mol per methylene group (−CH_2_−) added to the alkyl chain of the alkyl sulfate anions. Therefore, it is likely that both the hydrophobic effect and enhanced van der Waals contacts play important roles in stabilizing the resulting associates.

For all three C_n_–OSO_3_^–^/CTA^+^ systems studied, the enthalpy change Δ*H*_(ITC)_ is negative (Table 1). This indicates that the first step of molecular recognition is governed primarily by electrostatic attraction between oppositely charged species. However, the balance between enthalpy and entropy contributions to the overall stability depends on the length of the hydrophobic moiety of the C_n_–OSO_3_^–^ anion.

Only for the shortest alkyl chain sulfate, namely octyl sulfate (C_8_–OSO_3_^–^), the reaction is both enthalpy- and entropy-driven. In this case, the overall system becomes more disordered upon binding (*T*Δ*S*_(ITC)_ > 0), likely because the C8 chain is too short to engage in significant hydrophobic interactions with the CTA^+^ tail. Consequently, the CTA^+^/C_8_–OSO_3_^–^ complex probably forms a loose ion pair in which both reagents retain considerable conformational freedom.

In contrast, for the complexes with decyl sulfate (C_10_–OSO_3_^–^) and dodecyl sulfate (C_12_–OSO_3_^–^), the negative enthalpy overcompensates the unfavorable entropic contribution to the stabilization of the ion pair. Here, the overall system becomes more ordered upon binding (*T*Δ*S*_(ITC)_ < 0) due to interactions between the hydrophobic tails of C_n_–OSO_3_^–^ and CTA^+^. As a result, these tails lose conformational flexibility.

This pattern indicates that the longer the alkyl chain (C12 > C10 ≫ C8), the greater the reduction in flexibility of the reagents upon complex formation. The C_12_–OSO_3_^–^/CTA^+^ complex seems to be the most rigid and well-ordered, whereas the C_8_–OSO_3_^–^/CTA^+^ complex remains relatively flexible and dynamic.

#### CTAB/Na­[B­(Ph)_4_] Interactions

In contrast to alkyl sulfate anions (C_n_–OSO_3_^–^), which possess a linear, flexible hydrocarbon chain, tetraphenylborate ions (B­(Ph)_4_^–^) feature phenyl groups arranged tetrahedrally around a central boron atom. This rigid, bulky structure enables binding to the CTA^+^ cation (which contains a 16-carbon alkyl chain) through a combination of hydrophobic and electrostatic forces. The resulting complex has a 1:1 stoichiometry (*N* = [B­(Ph)_4_^–^]/[CTA^+^]), and the thermodynamic parameters for this interaction are as follows: log*K*_(ITC)_ = 6.42 (±0.05), Δ*G*_(ITC)_ = −8.75 (±0.02) [kcal/mol], Δ*H*_(ITC)_ = −19.93 (±0.15) [kcal/mol], and *T*Δ*S*_(ITC)_ = −11.18 [kcal/mol] (Figure 2).

**Figure 2:**
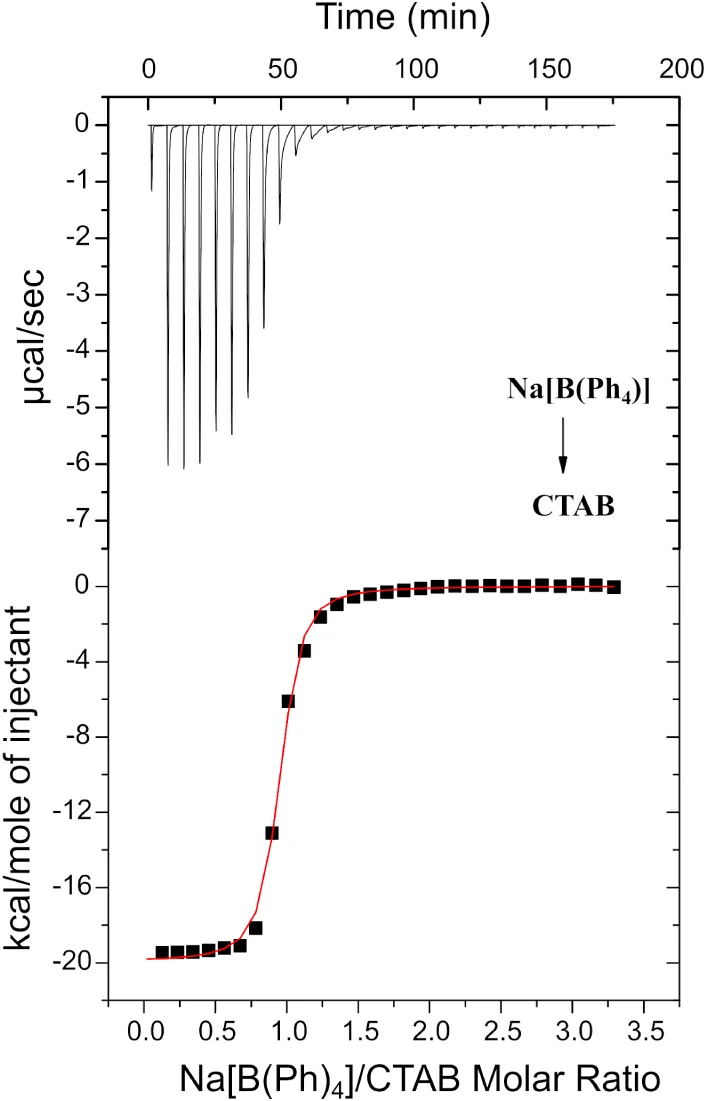
Calorimetric titration isotherms of binding interactions of hexadecyltrimethylammonium cation (CTA^+^) with tetraphenylborate ions (B­(Ph)_4_^–^) in water at 298.15 K.

The tetrahedral geometry of B­(Ph)_4_^–^ allows its phenyl rings to simultaneously engage in hydrophobic contacts and possibly π–cation interactions with both the tail and the headgroup of CTA^+^. The binding process is enthalpy-driven, with a negative entropic contribution (*T*Δ*S* < 0) to the overall stability. This indicates that binding leads to significant ordering of the system, as the flexible CTA^+^ tail becomes constrained around the rigid phenyl moieties.

Despite the loss of conformational freedom of the CTA^+^ cations (an unfavorable entropy change), the binding remains fairly strong because it is overcompensated by highly favorable enthalpy changes. Due to the combination of electrostatic attraction and extensive hydrophobic/π interactions, the resulting complex is more stable and ordered than any complex formed with a linear alkyl sulfate anion, irrespective of the alkyl chain length.

#### Contact Patterns between CTA^+^ and Counterions

The atom-resolved contact maps presented of Figure 3 show a common association motif for CTA^+^ and the alkyl sulfate anions.^30,73^ Namely, the most important contacts involve the chain segments located close to the polar groups. At the same time the contact probability decreases toward the terminal parts of the chains. The elongated diagonal region shows that the adjacent segments of the two hydrocarbon chains are often close to each other. This suggest significant chain alignment. Increasing the alkyl sulfate chain length extends this contact region, indicating larger hydrophobic surface available for association. The somewhat similar patterns observed for both studied compositions indicate that stoichiometry has little influence on the preferred local arrangement.

**Figure 3:**
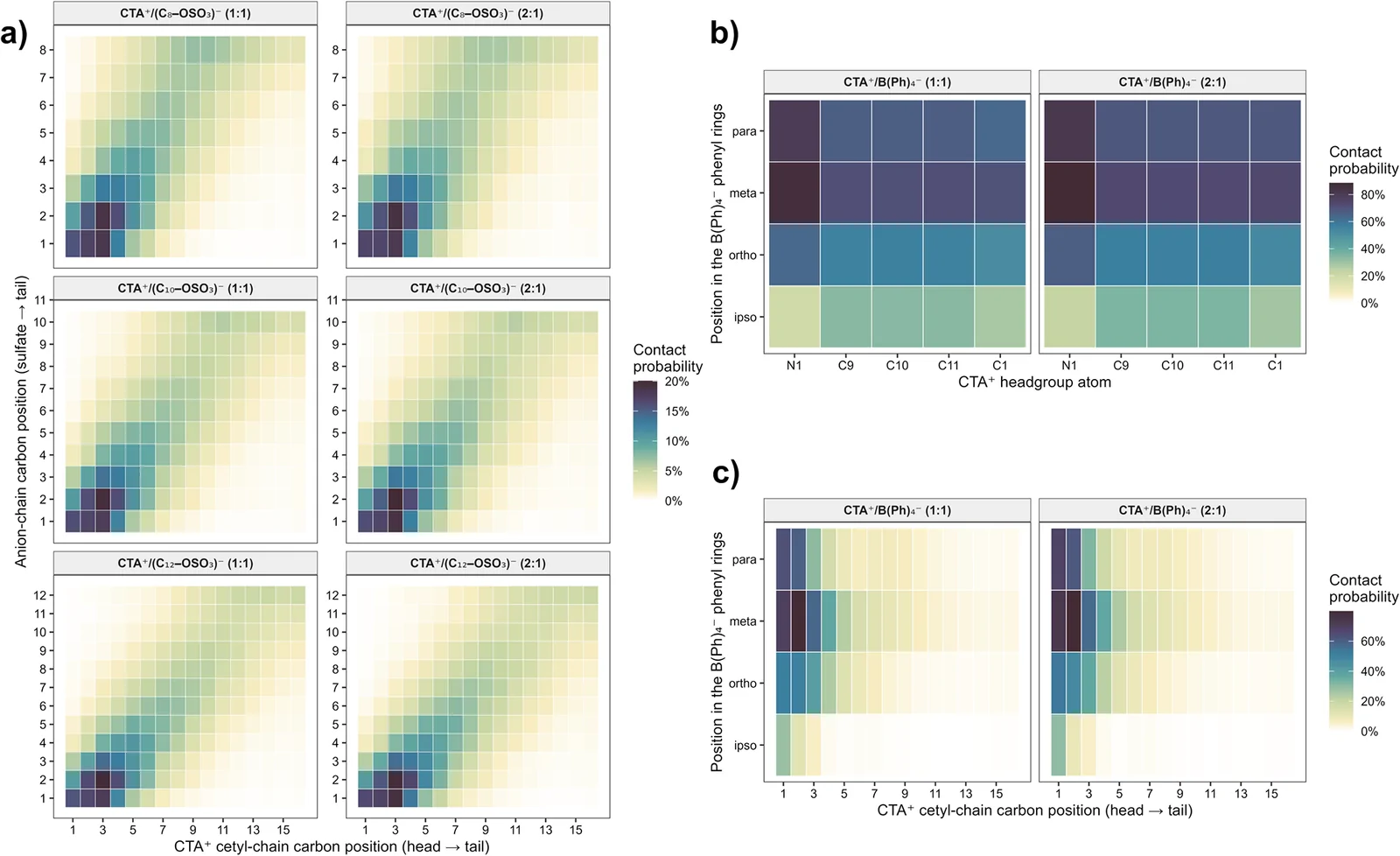
Atom-resolved contact probability maps for CTA^+^ with (a) alkyl sulfate chains and B­(Ph)_4_^–^ phenyl rings involving the (b) CTA^+^ headgroup and (c) cetyl chain.

The CTA^+^ headgroup forms broad contacts with the B­(Ph)_4_^–^ phenyl rings. The central nitrogen region shows a preference for the meta and para portions of the rings. Whereas contacts with the ipso positions are less significant. The adjacent headgroup carbon atoms display very similar patterns. This suggest that the interaction occurs over the phenyl surface rather than at one specific site. The same contact pattern is observed at both compositions. This indicates that the headgroup arrangement around B­(Ph)_4_^–^ is determined by the local CTA^+^-anion geometry rather than by the CTA^+^:anion ratio.

B­(Ph)_4_^–^ contacts with the CTA^+^ cetyl chain are located mainly near the headgroup part of the chain and decrease significantly toward the terminal methyl group. Meta and para sites ring positions form the most significant contacts, while the ortho sites show an intermediate contribution. At the same time the ipso sites are generally less involved. Interactions farther along the CTA^+^ chain suggest that the phenyl surface is in contact with a large part of the chain. The calculations for both ratios show the same pattern.

The maps indicate that B­(Ph)_4_^–^ engages both the cationic headgroup and the hydrocarbon chain of CTA^+^. At the same time, alkyl sulfate association involves close polar groups and aligned hydrocarbon chains.

#### Energetic Origin of CTA-Anion Association

The values of interaction energies calculated for the systems studied in this paper are collected in Table 2. Due to the ionic nature of studied systems, it comes as no surprise that regardless of the complex studied, the total interaction energy is dominated by electrostatic contribution. Namely, electrostatics accounts for no less than 62% of the total attractive interaction in each 1:1 complex studied here. This indicates that the interaction between the positively charged headgroup of CTA and the negatively charged head of the anion is the main factor stabilizing the system and agrees with the physicochemistry of ionic surfactants.^15,74^ For the complexes based on the alkyl sulfate homologues, the electrostatic contribution to the interaction energy spans a narrow 73–76 kcal/mol energy range. Each elongation of the alkyl chain increases the hydrophobic contact area allowing for stronger London-type interaction between the tails of the CTA and that of the anion. This is reflected in the values of dispersion contribution, which values increase as follows: 15.38, 15.71, and 19.45 kcal/mol for C8, C10 and C12 alkyl sulfates, respectively. The corresponding percentage contribution changes from 15.56 via 15.34 to 18.49% in the same order. The increase in Pauli repulsion reflects tighter packing, but it is compensated by the concomitant growth of dispersion. Importantly, the increasing dispersion contribution from C8 to C12 is mostly balanced by the increase in exchange, so the total 1:1 interaction energies change only slightly. The nearly constant induction term across the C8/C10/C12 series suggests, that electrostatics and induction constitute a common interaction baseline. Although SAPT results provide a more informative physical decomposition of the interaction energy, the CP-corrected supermolecular ωB97X-V/aug-cc-pVDZ results are more appropriate as reference total interaction energies. As regards the C8–C12 series, the values span from −85.83 to −88.04 and −87.28 to −89.32 kcal/mol for the SAPT and the supermolecular approach, respectively. Thus, it can be concluded that both approaches remain in very good agreement, differing by ca. 1.4–1.6 kcal/mol.

**Table 2:** The Energy Decomposition of the 1:1 CTA-Anion Complexes and Counterpoise-Corrected Interaction Energies for the Corresponding 2:1 Systems^a^

	1:1						2:1	
System	Electrostatics	Exchange	Induction	Dispersion	*E_int_*_(*SAPT*)_	*E_int_*_(*cp*)_	2-body *E_int_*_(*cp*)_	3-body *E_int_*_(*cp*)_
CTA^+^/(C_8_–OSO_3_)^−^	–73.59 (74.46)	13.01	–9.86 (9.98)	–15.38 (15.56)	–85.83	–87.28	–145.60	–137.33
CTA^+^/ (C_10_–OSO_3_)^−^	–76.21 (74.41)	14.60	–10.50 (10.25)	–15.71 (15.34)	–87.83	–89.32	–149.08	–140.73
CTA^+^/ (C_12_–OSO_3_)^−^	–75.54 (71.83)	17.13	–10.18 (9.68)	–19.45 (18.49)	–88.04	–89.62	–149.36	–141.15
CTA^+^/B(Ph)_4_^–^	–65.72 (62.43)	22.54	–11.74 (11.14)	–27.96 (26.52)	–82.88	–82.77	–133.93	–126.20

a Values in parentheses denote percentage contributions of individual interactions to the total attractive interaction of 1:1 complexes. The counterpoise-corrected interaction energies, *E_int_*_(*cp*)_ are also listed for the 1:1 complexes. For the 2:1 systems, the counterpoise-corrected two-body and three-body interaction energies are given. All values are given in kcal/mol.

For the alkyl sulfate complexes with 2:1 stoichiometry, the sum of pairwise interaction energies becomes progressively more favorable when going from C8 to C12 (−145.60 → −149.36 kcal/mol). A similar trend is observed for the 3-body CP-corrected interaction energies, which decrease from −137.33 to −141.15 kcal/mol. This indicates that elongation of the alkyl chain slightly stabilizes the trimeric complexes. The effect is relatively modest and is most pronounced between C8 and C10.

It is important to note, however, that the 3-body interaction energies are consistently less negative than the corresponding 2-body sums. The difference between these values, is positive for all alkyl sulfate systems: 8.27, 8.35, and 8.21 kcal/mol for C8, C10, and C12, respectively. Thus, the many-body contribution has a destabilizing effect on studied complexes and remains nearly constant across the sulfate series. It can be thus concluded that the increasing stability of the 2:1 complexes therefore originates mainly from more favorable pairwise interactions rather than from enhanced cooperative three-body effects. Altogether, it can be concluded that the SAPT results are in qualitative agreement with the ITC data, as both approaches indicate more prominent CTA^+^-anion association upon elongation of the alkyl sulfate chain.

As regards the CTA^+^/B­(Ph)_4_^–^, the SAPT energy contributions show a different picture. Namely, although the electrostatic term for CTA^+^/B­(Ph)_4_^–^ is ca. 10 kcal/mol smaller than for the alkyl sulfates, the tetraphenylborate anion regains much of the lost stabilization through a pronounced dispersion contribution and relatively strong induction, which account for 26.52 and 11.4% respectively, of the total attractive interaction. This is consistent with the behavior expected for a large, soft and highly polarizable aromatic counterion capable of additional CH···π, π···alkyl, and local cation-π interactions. The values of *E_int_*_(*SAPT*)_ and *E_int_*_(*cp*)_ calculated for this system are equal to −82.88 and −82.77 kcal/mol respectively. These values make the system least bound out of all 1:1 complexes studied. For the borate 2:1 complex, the 3-body *E_int_*_(*cp*)_ values are again less attractive (by 8 kcal/mol) than the corresponding 2-body terms, indicating that the pairwise stabilization is only partially retained in the full trimer because of non-additive effects.

The Gibbs free energies of association (Δ*G_assoc_*) of all studied systems are collected in Table 3. The first thing that stands out is that all values calculated in implicit solvation are slightly positive. This reflects heavy desolvation penalty associated with ion-pair formation. For that reason, the positive values of Δ*G_assoc_* should not be viewed as evidence of association. They rather constitute proof of the limitations of implicit solvation that does not explicitly account for first-shell hydration or hydrophobic organization. Hence, these values are most useful for comparing relative trends rather than absolute binding free energies.^75,76^

**Table 3:** Gibbs Free Energies of Association for 1:1 and 2:1 CTA^+^-Anion Complexes Given in kcal/mol

	Δ*G_assoc_*	
System	1:1	2:1
CTA^+^/(C_8_–OSO_3_)^−^	5.10	9.73
CTA^+^/ (C_10_–OSO_3_)^−^	1.86	7.16
CTA^+^/ (C_12_–OSO_3_)^−^	2.92	5.60
CTA^+^/B(Ph)_4_^–^	4.11	12.04

The calculated Gibbs free energies of association indicate that, among the studied systems, CTA^+^ forms the most favorable 1:1 complex with C_10_–OSO_3_^–^ and 2:1 complex with C_12_–OSO_3_^–^. Thus, the 1:1 alkyl sulfate series does not show a monotonic dependence on chain length, as association becomes more favorable from C8 to C10, but becomes less favorable again for C12. This suggests that the C10 chain provides the most favorable balance between electrostatic attraction, hydrophobic interactions, conformational flexibility, and solvation effects.^77^ The regular decrease in Δ*G_assoc_* values observed for the 2:1 series likely reflects the increasing ability of longer alkyl chains to compensate for the electrostatic penalty. This likely happens through extended hydrophobic and dispersion contacts.^78^ However, because the 2:1 complexes remain positively charged, they are still less favorable than the corresponding neutral 1:1 ion pairs. Among the 2:1 systems, CTA^+^/B­(Ph)_4_^–^ shows the least favorable association, suggesting that this anion does not efficiently stabilize complexes containing two CTA^+^ cations.

Comparison of the calculated Δ*G_assoc_* values with the ITC data shows partial qualitative agreement between the two kinds of results. Namely, the calculations indicate that 1:1 complexes are more favorable than the corresponding 2:1 systems, which is consistent with the ITC stoichiometries close to unity. Additionally, both approaches identify CTA^+^/C_8_–OSO_3_^–^ as the weakest alkyl sulfate complex. This confirms that the shortest alkyl chain provides the smallest hydrophobic stabilization. On the other hand, the calculated trend does not fully reproduce the experimental one. Namely, ITC results show an increase in binding strength with alkyl chain length. Computationally this observation is true only for the 2:1 series. This suggests that the calculations capture local ion pair stabilization but underestimate the hydrophobic effects. It also needs to be mentioned here, that the largest difference is observed for tetraphenylborate. Experimentally, CTA^+^/B­(Ph)_4_^–^ is comparable in stability to CTA^+^/C_12_–OSO_3_^–^, whereas DFT calculations give a higher Δ*G_assoc_* value for CTA^+^/B­(Ph)_4_^–^ than for the C10- and C12-alkyl sulfate complexes. The issues with implicit solvation identified here are consistent with previous results regarding the limitations of implicit solvation Gibbs free energy calculations.^79^ Additionally, one has to bear in mind that the discrepancy most likely reflects the fact that ITC provides an ensemble-averaged association free energy in solution, whereas the calculated Δ*G_assoc_* shown in this paper describe a representative low Gibbs energy ion pair minimum in implicit solvent.

Results obtained within the framework of this paper may form a basis for considerations regarding different anion classes. In the case of small inorganic anions, such as halides or nitrates, hydrophobic interactions with the alkyl chain of CTA^+^ are absent. Due to that, the association of these anions is expected to depend rather on factors such as ion hydration or polarizability. Organic counterions such as benzoate, salicylate or tosylate, which combine an ionic headgroup with hydrophobic aromatic ring may exhibit additional stabilization. Therefore, their behavior is expected to be similar to that of the alkyl sulfates studied in this work.

#### Structural Organization and Intermolecular Contacts

The equilibrium structures of the studied complexes, together with selected intermolecular contacts identified from the QTAIM analysis are presented in Figure 4. Since the complete QTAIM molecular graphs of these already intertwined complexes would include a dense network of intermolecular BCPs and bond paths, displaying all of them would obscure the molecular geometry. Therefore, only contacts corresponding to the largest estimated interaction energy within each interaction region are highlighted.

**Figure 4:**
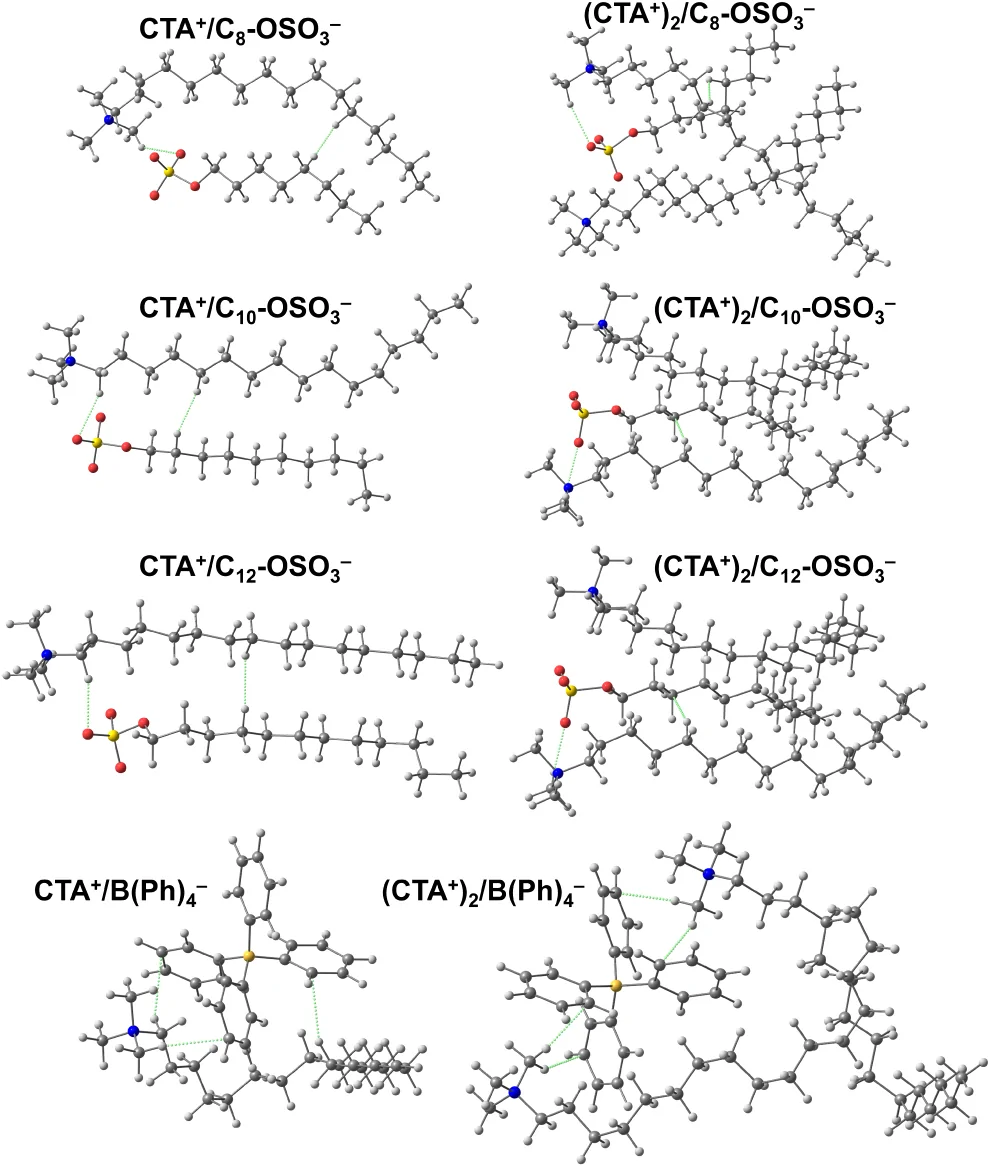
The equilibrium structures of the minimum Gibbs free energy complexes with strongest intermolecular contacts of each group identified from the QTAIM.

For the purpose of the QTAIM analysis, the intermolecular CTA^+^-anion BCPs were grouped according to predefined interaction regions (vide Table 4). Namely, in the alkyl sulfate complexes, the contacts were divided into headgroup and alkyl-chain regions. The former includes contacts between the sulfate group and the ammonium part of CTA^+^. Whereas the latter corresponds to contacts between the hydrocarbon fragments of the anion and the cetyl chain of CTA^+^. In contrast, for tetraphenylborate the head/chain classification is not applicable. Thus, the contacts were grouped according to the phenyl ring regions involved in the interaction.

**Table 4:** QTAIM Descriptors of the Weakest and Strongest CTA^+^-Anion Bond Critical Points (BCPs) within Each Contact Class^a^

System	Contact region	*n_BCPs_*	Δ^2^ *ρ_min_*	${\frac{|V_{BCP}|}{G_{BCP}}}_{\min}$	*E_Imin_*	Δ^2^ *ρ_max_*	${\frac{|V_{BCP}|}{G_{BCP}}}_{\max}$	*E_Imax_*
CTA^+^/C_8_–OSO_3_^–^	head	6	0.0078	0.6820	0.32	0.0305	0.7427	1.41
	chain	11	0.0071	0.6336	0.26	0.0161	0.8181	0.87
CTA^+^/C_10_–OSO_3_^–^	head	6	0.0107	0.7231	0.48	0.0291	0.7474	1.36
	chain	12	0.0077	0.6772	0.31	0.0148	0.7447	0.69
CTA^+^/C_12_–OSO_3_^–^	head	5	0.0106	0.6849	0.43	0.0405	0.7535	1.92
	chain	21	0.0049	0.5741	0.15	0.0155	0.7839	0.78
CTA^+^/B(Ph)_4_^–^	ring 1	6	0.0052	0.6526	0.20	0.0173	0.7702	0.85
	ring 2	9	0.0089	0.7032	0.38	0.0189	0.7906	0.97
	ring 3	4	0.0082	0.6706	0.32	0.0173	0.7931	0.89
(CTA^+^)_2_/C_8_–OSO_3_^–^	head	11	0.0115	0.7324	0.52	0.0346	0.7392	1.59
	chain	23	0.0040	0.5908	0.13	0.0196	0.8240	1.08
(CTA^+^)_2_/C_10_–OSO_3_^–^	head	9	0.0070	0.6984	0.29	0.0333	0.7410	1.54
	chain	25	0.0040	0.5775	0.13	0.0202	0.7871	1.03
(CTA^+^)_2_/C_12_–OSO_3_^–^	head	9	0.0067	0.6955	0.28	0.0346	0.7414	1.60
	chain	26	0.0046	0.5762	0.15	0.0201	0.7855	1.02
(CTA^+^)_2_/ B(Ph)_4_^–^	ring 1	5	0.0106	0.7154	0.46	0.0243	0.7926	1.25
	ring 2	3	0.0107	0.7759	0.53	0.0225	0.7840	1.14
	ring 3	9	0.0068	0.6116	0.24	0.0250	0.7898	1.28
	ring 4	2	0.0134	0.7579	0.64	0.0249	0.7899	1.28

a *n_BCPs_* denotes the number of intermolecular CTA^+^-anion BCPs. For the BCPs with the minimum and maximum interaction energy, corresponding ∇^2^ρ and $\frac{\left|V_{BCP}\right|}{G_{BCP}}$ and *E_I_* are reported. The ∇^2^ρ values are given in atomic units, $\frac{\left|V_{BCP}\right|}{G_{BCP}}$ is a dimensionless quantity , whereas *E_I_* is in kcal/mol.

Table 4 shows that CTA^+^-anion association is not controlled by a single dominant contact, but by the balance between relatively stronger polar contacts and a significant number of weak hydrophobic contacts.^80–82^ For the 1:1 alkyl sulfate complexes, the strongest headgroup region contacts are consistently stronger than the strongest chain contacts. The maximum estimated contact energy in the head region is 1.41 kcal/mol for C_8_–OSO_3_^–^, 1.36 kcal/mol for C_10_–OSO_3_^–^, and increases to 1.92 kcal/mol for C_12_–OSO_3_^–^. By contrast, the strongest chain contacts remain weaker, with maximum values of only 0.87, 0.69, and 0.78 kcal/mol, respectively. This seems to suggest that the sulfate/CTA^+^ headgroup region acts as the local anchoring site of the whole complex. Whereas the alkyl chains contribute through somewhat weaker dispersive contacts. It must be noted here, that the chain elongation does not systematically strengthen individual chain BCPs. It does however increases their number. The number of chain region BCPs rises from 11 for C8 to 12 for C10 and then significantly to 21 for C12. The number of chain region BCPs rises from 11 for C8 to 12 for C10 and then considerably to 21 for C12. Thus, it can be said that C12 leads to greater stabilization because its longer chain can form more weak hydrophobic contacts.

This is also supported by the results obtained for 2:1 complexes. Addition of the second CTA^+^ increases the number of intermolecular BCPs, especially in the chain region (23, 25, and 26 for C8, C10, and C12, respectively). The strongest chain contacts in these 2:1 systems are somewhat larger than in the corresponding 1:1 complexes. Namely, they reach about 1.02–1.08 kcal/mol. The second CTA^+^ mainly adds more weak contacts around the anion. This leads to the conclusion that the larger number of BCPs in 2:1 structures reflects increased contact surface and molecular size. It should however not be taken as direct evidence that the 2:1 complexes are thermodynamically preferred.

For B­(Ph)_4_^–^, the trend is different. Namely, the 1:1 complex contains 19 CTA^+^-anion BCPs distributed over three phenyl ring contact regions, with maximum contact energies between 0.85 and 0.97 kcal/mol. These contacts are somewhat weaker than the strongest sulfate-headgroup contacts, and comparable to the alkyl-chain contacts in the sulfate systems. In the 2:1 B­(Ph)_4_^–^ complex, the total number of ring-associated BCPs remains 19, rather than increasing strongly as in the alkyl sulfate 2:1 complexes. However, the strongest contacts become somewhat larger, reaching 1.14–1.28 kcal/mol. This suggests that B­(Ph)_4_^–^ forms several localized contacts with aromatic surfaces, involving C–H···π and alkyl/aromatic interactions (see Figure 4).

Across all studied systems, the QTAIM descriptors corresponding to the BCPs indicate closed-shell character. The positive ∇^2^ρ values and the values of |V|/G below 1 show that contacts remain weak and noncovalent.^83^ The highest |V|/G values approach about 0.82, but they do not surpass the limit associated with covalent character. Thus, the main conclusion from this analysis is that longer chains create extensive networks of weak dispersion dominated contacts.^80–82^

#### Electronic Response and Polarity of CTA-Anion Complexes

The analysis of the electronic response descriptors collected in Table 5 and the values calculated for the isolated ions shown in Table S2, give insights into the stabilization of CTA^+^-anion complexes by anions. Namely, in the alkyl sulfate series, the staticpolarizability of the isolated anions increases with the alkyl chain length from 181.42 a.u. for C_8_–OSO_3_^–^ via 213.34 a.u. for C_10_–OSO_3_^–^ to 246.36 a.u. for C_12_–OSO_3_^–^. Similar trend is observed for the corresponding polarizability anisotropy. In particular, it increases from 9.69 to 13.67 and 17.73 a.u., respectively. Therefore, it can be concluded that elongation of the hydrocarbon chain enhances both the static polarizability and directionality of the response.

**Table 5:** Isotropic Polarizability, Polarizability Anisotropy (in a.u.) Calculated for 1:1 and 2:1 CTA^+^-Anion Complexes

	1:1		2:1	
System	*α_iso_*	*γ*	*α_iso_*	*γ*
CTA^+^/(C_8_–OSO_3_)^−^	496.48	40.82	811.97	37.32
CTA^+^/ (C_10_–OSO_3_)^−^	529.66	53.74	844.82	44.71
CTA^+^/ (C_12_–OSO_3_)^−^	562.84	50.70	877.93	45.78
CTA^+^/B(Ph)_4_^–^	743.53	66.47	1059.22	63.30

This trend continues in the CTA^+^-alkyl sulfate complexes. Namely, both 1:1 and 2:1 stoichiometry systems show increasing *α_iso_* values while going from C_8_–OSO_3_^–^ to C_12_–OSO_3_^–^. This reflects growing molecular size and thus larger hydrophobic surface available for dispersion-type interactions. This finding is consistent with the SAPT results showing that the dispersion contribution to total attractive energy increases with increasing alkyl chain length. In the alkyl sulfate series, this trend links the increasing isolated anion polarizability (181.42, 213.34, and 246.36 a.u.), growing SAPT dispersion contribution (15.38, 15.71, and 19.45 kcal/mol) and the larger number of chain region BCPs in the 1:1 complexes (11, 12 and 21). Thus, it can be concluded that the enhanced stabilization of the longer chain systems, especially C12, appears to arise mainly from the cumulative buildup of many weak hydrophobic contacts rather than from strengthening of a single dominant interaction. The formation of 2:1 complexes is naturally accompanied by higher *α_iso_* values compared with the corresponding 1:1 systems, due to the presence of the additional CTA^+^ cation.

The tetraphenylborate anion differs substantially from the alkyl sulfate anions. Namely, due to the presence of four phenyl rings its isolated *α_iso_* value is equal to 428.87 a.u., and as such is much higher than that of alkyl sulfates. At the same time, the isolated B­(Ph)_4_^–^ anion has a relatively low γ value of 7.06 a.u., consistent with its tetrahedral and relatively isotropic structure. However, upon complex formation with CTA^+^, the polarizability anisotropy increases significantly. This indicates that the spatial arrangement of CTA^+^ around B­(Ph)_4_^–^ breaks the nearly isotropic character of the isolated anion. Thus, although isolated B­(Ph)_4_^–^ is only weakly anisotropic, the CTA^+^/B­(Ph)_4_^–^ complexes display pronounced anisotropic electronic response.

Comparison of the complex polarizabilities with the sums of the corresponding isolated ion values shows that *α_iso_* values are nearly additive. For the 1:1 complexes, the differences between the calculated complex *α_iso_* values and the sums *α_iso_* (CTA^+^) + *α_iso_* (anion) do not exceed ca. 0.35%. Similarly, for the 2:1 systems, the deviations from 2*α_iso_* (CTA^+^) + *α_iso_* (anion) remain below ca. 0.30%. This indicates that complex formation does not substantially change the magnitude of static polarizability. It also proves that *α_iso_* is controlled mainly by both the size and composition of the complex.

In contrast, polarizability anisotropy is clearly non-additive. The γ values calculated for the complexes differ substantially from the simple sums of the isolated ion γ values. This effect is particularly evident for the 2:1 complexes, where the calculated γ values are much lower than the scalar sums of the isolated components. Such behavior is expected because γ depends both on the magnitude of the individual polarizability tensors and their mutual orientation. Thus, it can be concluded that the anisotropy of static polarizability is controlled by the spatial organization.

Overall, the electronic-response descriptors show that the anion structure modulates CTA^+^ association. Longer alkyl sulfates increase the polarizability and promote more directional hydrophobic organization. At the same time, B­(Ph)_4_^–^ was found to contribute a large polarizability and additionally induce strong anisotropic response upon complex formation with CTA^+^. It also needs to be noted that the polarizabilities studied here are static. Inclusion of dynamic solvent effects could also refine the presented results.

## Conclusions

Despite the model-system limitations, the results presented in this paper provide a molecular level explanation for counterion-specific stabilization in CTA^+^ ion pairs. Combining ITC with molecular dynamics and quantum chemical calculations enabled a thorough analysis of these interactions. The key conclusions drawn from the results are as follows:

i. All studied ion pairs are dominated by electrostatics. However, the alkyl sulfates and tetraphenylborate complexes gain significant stabilization from dispersion.
ii. As indicated by ITC and supported by the quantum-chemical interaction analyses, increasing the alkyl sulfate chain length strengthens CTA binding through more hydrophobic contacts and larger London dispersion contributions.
iii. The thermodynamics of C8–OSO_3_^–^ association suggest a relatively loose ion pair with significant residual conformational freedom. In contrast, C10 and C12 binding are strongly enthalpy-driven but entropically penalized.
iv. The equilibrium structures and corresponding QTAIM results show two recurring stabilizing regions: the CTA^+^ headgroup that associates with either the anionic head or tetrahedral tetraphenylborate and the hydrophobic chain interacting via numerous weak contacts.
v. The electronic-response and polarity descriptors indicate that anion structure modulates CTA^+^ association beyond simple charge compensation. Namely, longer alkyl sulfates promote more directional polarizability and hydrophobic organization, whereas B­(Ph)_4_^–^ supplies aromatic polarizability.

Investigating how molecular-level interaction trends translate into higher-order self-organization is an important direction for future research.

## Supplementary Material



## Notes

MD stability checks, contact-analysis details, Cartesian coordinates of low energy complexes, and polarizability data (PDF)
