# Supplementary material for: Counterion Structure Tunes Cetyltrimethylammonium Association in Water: Thermodynamics and Weak-Contact Networks
Source: Langmuir. 2026 Oct 1;42(40):30124–35. doi: 10.1021/acs.langmuir.6c04226 (PMC13674275; doi:10.1021/acs.langmuir.6c04226)
Supplement: Supplementary file 1 [file la6c04226_si_001.pdf]

Supporting Information

Counterion Structure Tunes Cetyltrimethylammonium Association in Water: Thermodynamics and Weak-Contact Networks

Jakub Brzeski[1]*, Sylwia Freza[1], Dariusz Wyrzykowski[1]

[1]Faculty of Chemistry, University of Gdańsk, Wita Stwosza 63, 80-308 Gdańsk, Poland

*Correspondence: jakub.brzeski@ug.edu.pl

Contents

Production MD Stability Checks 2

CTA^+-anion contact analysis 4

Selection of MD Representatives 5

Cartesian coordinates 6

References 110

Production MD Stability Checks

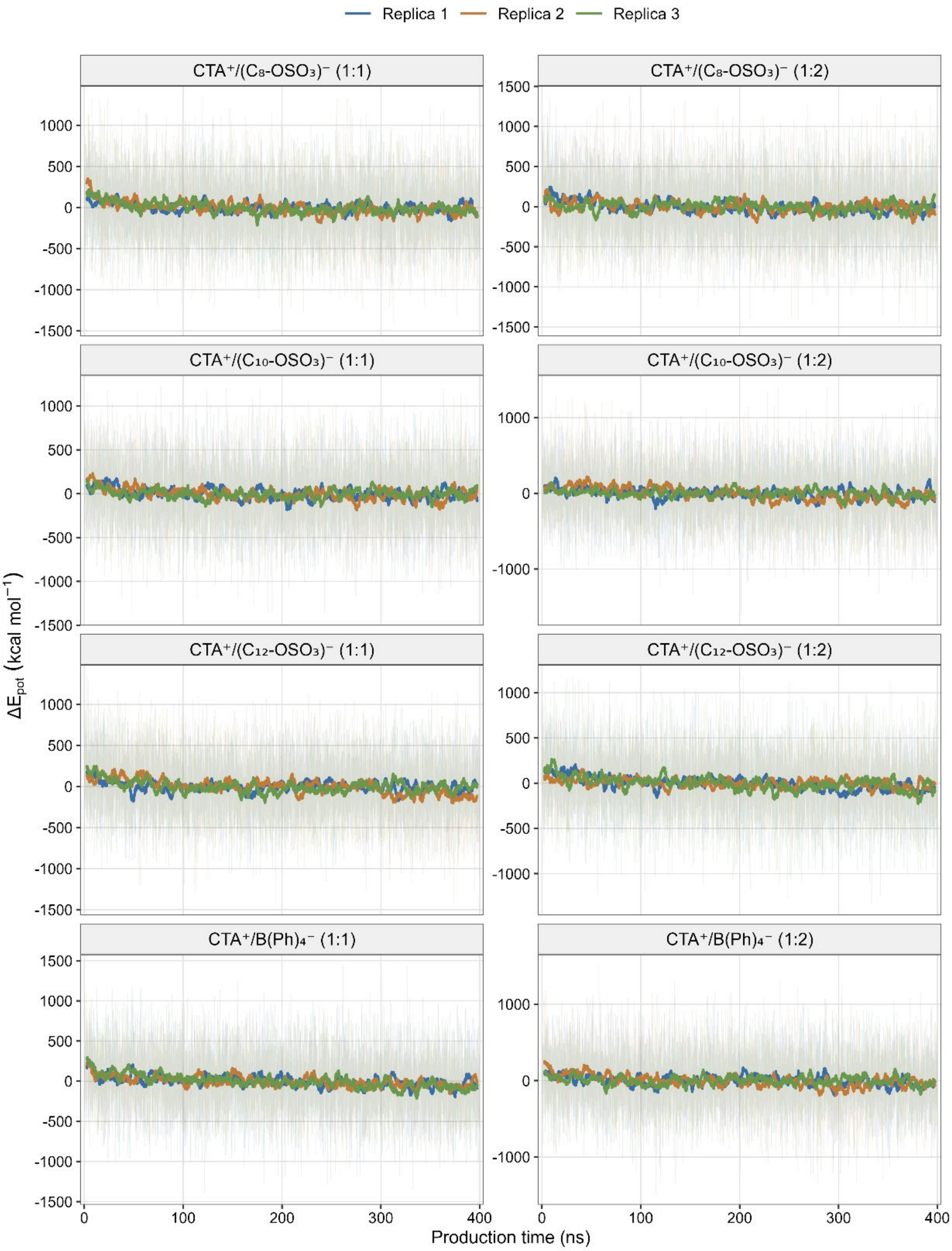

Figure S1. Time evolution of the mean-centered potential energy during the 400 ns production MD simulations. For each trajectory, the potential energy is expressed as $\Delta E_{\mathrm{pot}}(t) = E_{\mathrm{pot}}(t) - \langle E_{\mathrm{pot}} \rangle$). Thin lines show 100 ps data, and solid lines show 5 ns moving averages.

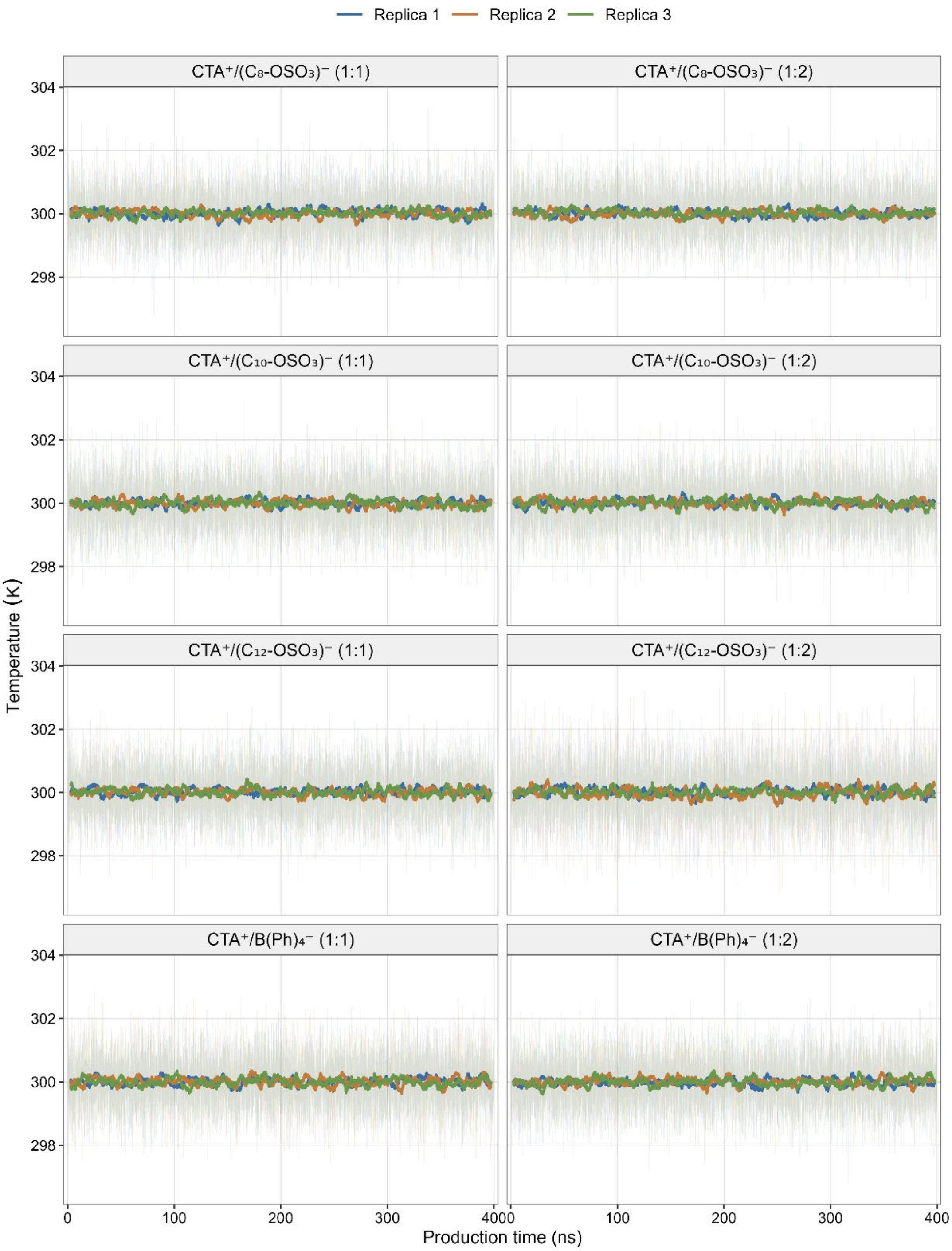

Figure S2. Temperature during the 400 ns production MD simulations. Thin lines show 100 ps data, and solid lines show 5 ns moving averages.

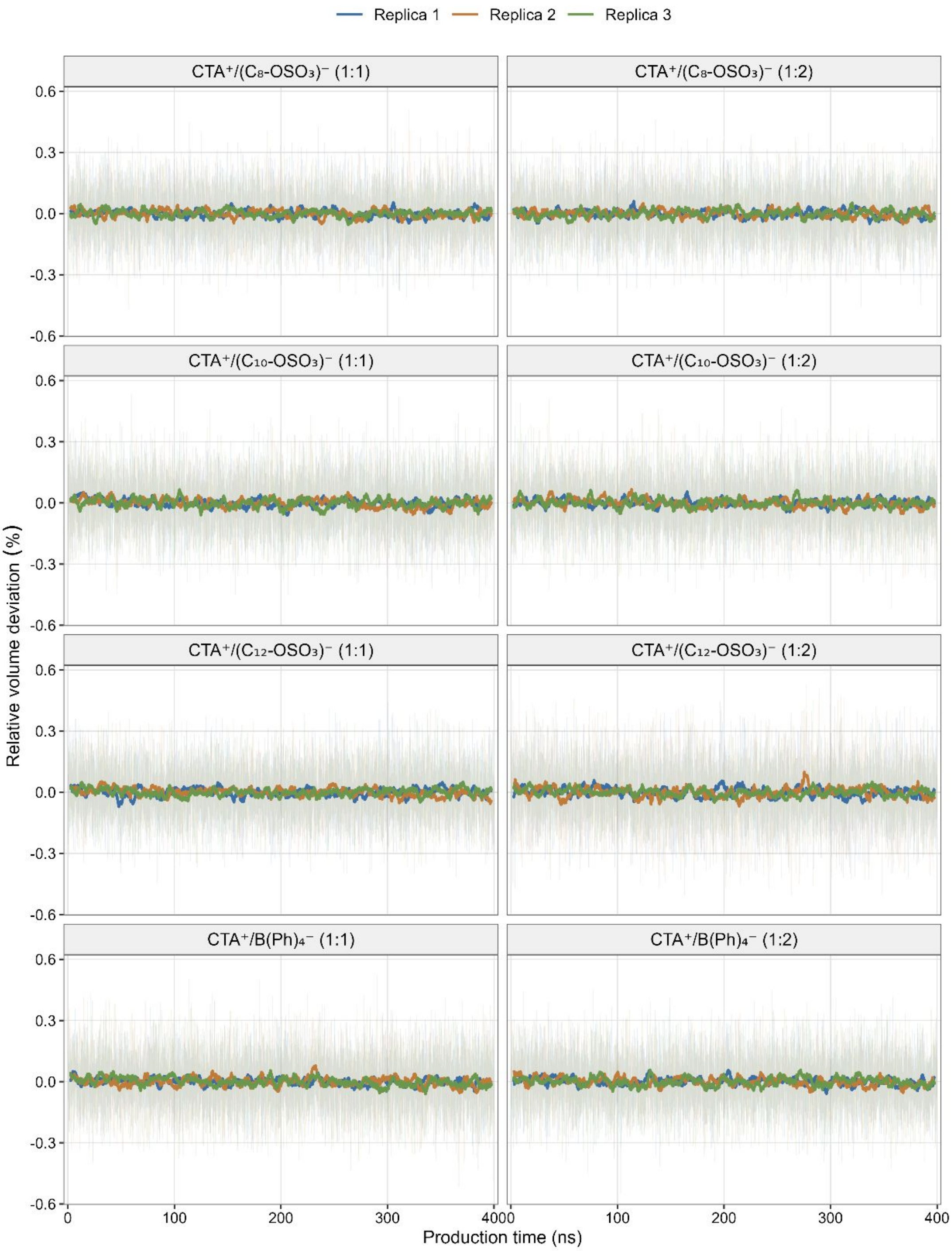

Figure S3. Relative simulation-cell volume deviation during the 400 ns NPT production MD simulations, reported as $100[V(t)/\langle V\rangle - 1]\%$. Thin lines show 100 ps data, and solid lines show 5 ns moving averages.

CTA^+-anion contact analysis

Distances for pair association and contact assignment were calculated using the nearest periodic image of each atom. The analysis included only locally associated CTA^+-anion pairs, which were identified from the first minima of the N···S and N···B radial distribution functions: N···S ≤ 6.5 Å for

alkyl sulfates and N···B ≤ 8.0 Å for $B(Ph)_4^-$. Contact matrices were created using a 4.5 Å cutoff. For alkyl sulfate systems, chain carbons were ordered from the headgroup to the terminal methyl group, and headgroup contacts were defined using sulfate oxygen atoms and the CTA^+ nitrogen atom with neighboring carbons.

For $B(Ph)_4^-$, phenyl carbons were grouped as ipso, ortho, meta, or para, and their contacts with the CTA^+ headgroup and cetyl chain were evaluated separately. A CTA^+ chain was considered aligned with the phenyl surface when at least three of its chain atoms contacted phenyl carbon atoms within 4.5 Å. Contact probabilities were first calculated for each simulation and then averaged equally over the three simulations. Thus, the maps show contacts only for locally associated CTA^+-anion pairs.

Selection of MD Representatives

The structures of ion-associated complexes were extracted from the final 200 ns of each trajectory and pooled separately for each CTA^+:anion composition[1,2]. Candidates from all three independent simulations were included. Each candidate was described by translation- and rotation-invariant geometric descriptors. For 1:1 complexes, these included CTA^+-anion distances, shortest polar and C···C contacts, the number of intermolecular C···C contacts below 4.5 Å and the number of CTA^+ ions in the anion coordination shell. For 2:1 complexes, the corresponding descriptors were evaluated for both CTA^+ ions, together with the N···N distance and the N-S-N or N-B-N angle.

Afterwards, the descriptors were robust-scaled using the median and interquartile range and clipped to the range -6 to +6. Each of the eight candidate pools was clustered independently by k-means in the scaled descriptor space using k=80 clusters, k-means++ initialization, a maximum of 100 iterations, and a fixed random seed of 20260611.[3,4] The resulting clusters served only as a basis for selecting structurally diverse representatives and were not assigned physical meaning. Up to three structures were selected from each cluster as the actual MD frames closest to the cluster centroid. When possible, selected structures were taken from different independent simulations, otherwise, the next closest frames were used. No averaged geometries were generated.

Selected complexes were reconstructed across periodic boundaries by making molecules whole, rebuilding anions around S or B atoms and CTA^+ ions around N atoms, and placing the cations relative to the anion using the minimum-image convention[2]. Each complex was translated so that the S or B atom was at the origin. Water molecules and free ions were removed. The final XYZ files contained CTA^+ followed by the anion for 1:1 complexes, and two CTA^+ ions followed by the anion for 2:1 complexes. The slightly smaller datasets for different stoichiometries arose from the fact that a few clusters contained fewer than three unique candidates; missing representatives were not replaced by duplicated frames.

Thus created XYZ files were checked for: the number of atoms, consistency between the header and coordinate count, finite coordinates, molecular continuity after periodic-boundary correction, intramolecular bond lengths not exceeding 2.5 Å, and the absence of nonphysical atomic overlaps below 0.55 Å. All 1914 structures passed these checks.

Cartesian coordinates

Table S1. Cartesian coordinates (in Å) of the five lowest energy isomers of the 1:1 and 2:1 CTA-anion complexes calculated at the ωB97X-3c level using approximation and CPCM solvation. The ΔG (in kcal/mol) stands for relative Gibbs free energies for presented isomers with respect to their corresponding global minima.

**1- $CTA^+/(C_8\text{-}OSO_3)^-$ 1:1 ΔG=0.0**			
7	0.660082000	-1.907253000	-3.883154000
6	-0.827023000	-1.907376000	-3.648401000
6	-1.347561000	-3.006612000	-2.752371000
6	-2.866872000	-3.048839000	-2.833082000
6	-3.500336000	-3.972676000	-1.804010000
6	-3.484198000	-3.420193000	-0.389204000
6	-4.130744000	-4.349025000	0.621280000
6	-4.179901000	-3.765893000	2.021115000
6	-4.816704000	-4.694465000	3.038429000
6	1.406533000	-1.944448000	-2.599134000
6	1.005087000	-0.652262000	-4.603489000
6	1.063385000	-3.064043000	-4.724169000
6	-4.920790000	-4.082973000	4.423165000
6	-5.553418000	-5.014988000	5.442045000
6	-5.769108000	-4.387135000	6.810708000
6	-4.491706000	-4.072382000	7.569079000
6	-4.752855000	-3.435388000	8.921399000
6	-3.486360000	-3.078501000	9.676062000
6	-3.752796000	-2.432419000	11.023157000
6	-2.482865000	-2.036647000	11.751967000
1	-1.277196000	-1.959906000	-4.638074000
1	-1.061770000	-0.938003000	-3.213931000
1	-0.954177000	-3.979896000	-3.046591000
1	-1.031617000	-2.816632000	-1.728555000
1	-3.152103000	-3.367874000	-3.837814000
1	-3.268158000	-2.040441000	-2.702929000
1	-4.537605000	-4.161252000	-2.091247000
1	-2.996333000	-4.943718000	-1.827000000
1	-2.458950000	-3.211924000	-0.074445000
1	-4.006386000	-2.458576000	-0.380901000
1	-5.148007000	-4.587279000	0.295541000
1	-3.586847000	-5.298515000	0.641077000
1	-4.734144000	-2.822470000	1.997498000
1	-3.165996000	-3.512987000	2.346494000
1	-4.239736000	-5.622929000	3.092384000
1	-5.816320000	-4.976495000	2.693435000

1	1.237182000	-2.900084000	-2.116751000
1	1.051561000	-1.139835000	-1.964204000
1	2.462608000	-1.824188000	-2.819068000
1	2.069730000	-0.655890000	-4.814205000
1	0.747389000	0.190789000	-3.970582000
1	0.437503000	-0.617953000	-5.528073000
1	0.521381000	-3.015881000	-5.663236000
1	2.131870000	-2.998071000	-4.903258000
1	0.834762000	-3.985199000	-4.201707000
1	-3.924227000	-3.782591000	4.755718000
1	-5.512102000	-3.163940000	4.364942000
1	-6.517655000	-5.352988000	5.052629000
1	-4.936035000	-5.912794000	5.546655000
1	-6.379729000	-5.059078000	7.419923000
1	-6.352512000	-3.468257000	6.693456000
1	-3.860219000	-3.403351000	6.979717000
1	-3.916707000	-4.993872000	7.704831000
1	-5.353890000	-2.531733000	8.779797000
1	-5.359776000	-4.112204000	9.530724000
1	-2.880941000	-2.401113000	9.065496000
1	-2.881971000	-3.979485000	9.820591000
1	-4.333037000	-3.121041000	11.643194000
1	-4.381058000	-1.549718000	10.878427000
1	-2.701676000	-1.579629000	12.717080000
1	-1.905101000	-1.319479000	11.166691000
1	-1.847416000	-2.905474000	11.930709000
16	-1.168308000	0.415563000	-0.260664000
8	-1.726375000	0.941718000	1.169260000
8	-0.266422000	1.497756000	-0.647402000
8	-0.496717000	-0.855739000	0.012942000
8	-2.340816000	0.275539000	-1.126298000
6	-4.333117000	0.535703000	5.299653000
6	-5.222727000	-0.342763000	6.161384000
6	-3.922609000	-0.132415000	3.999857000
6	-5.718316000	0.322629000	7.435909000
6	-3.012708000	0.742778000	3.158448000
6	-4.625023000	0.658294000	8.435672000
6	-2.621527000	0.070065000	1.868797000
6	-5.175089000	1.227553000	9.729724000
1	-4.856654000	1.470418000	5.075564000
1	-3.432902000	0.813426000	5.852719000
1	-6.084890000	-0.659144000	5.567994000
1	-4.681687000	-1.257095000	6.421574000
1	-4.816707000	-0.392768000	3.426239000
1	-3.415399000	-1.075719000	4.221214000
1	-6.441603000	-0.339292000	7.920478000
1	-6.265421000	1.235847000	7.180450000
1	-2.108118000	0.982510000	3.721397000
1	-3.509630000	1.688731000	2.932578000
1	-4.046827000	-0.244910000	8.649230000
1	-3.925355000	1.372180000	7.996895000

1	-2.117302000	-0.877716000	2.060431000
1	-3.496205000	-0.120072000	1.245236000
1	-4.378352000	1.451483000	10.439389000
1	-5.858307000	0.523369000	10.207433000
1	-5.727823000	2.150625000	9.546720000
**2- CTA$^+$/(C$_8$-OSO$_3$)$^-$ 1:1 ΔG=1.00**			
7	-3.801744000	-1.748617000	-1.699103000
6	-4.460965000	-0.412780000	-1.909683000
6	-4.828523000	-0.079285000	-3.335234000
6	-5.364458000	1.341283000	-3.405642000
6	-5.726151000	1.754330000	-4.819338000
6	-6.174202000	3.200140000	-4.917728000
6	-6.459756000	3.643743000	-6.340132000
6	-6.783532000	5.122005000	-6.452916000
6	-6.981176000	5.585528000	-7.887886000
6	-4.689731000	-2.857107000	-2.130270000
6	-3.525176000	-1.888887000	-0.243456000
6	-2.509715000	-1.827833000	-2.430804000
6	-6.866923000	7.091389000	-8.066534000
6	-7.916818000	7.895962000	-7.319077000
6	-7.790926000	9.400131000	-7.515584000
6	-6.529906000	9.992157000	-6.909803000
6	-6.447753000	11.502273000	-7.047691000
6	-5.207253000	12.112517000	-6.411681000
6	-3.905406000	11.767107000	-7.114848000
6	-2.695820000	12.384148000	-6.438457000
1	-3.765416000	0.323392000	-1.515367000
1	-5.347736000	-0.418426000	-1.278379000
1	-3.958358000	-0.154891000	-3.986923000
1	-5.582536000	-0.769562000	-3.714111000
1	-4.608975000	2.026873000	-3.013612000
1	-6.239737000	1.437324000	-2.757647000
1	-4.856558000	1.605370000	-5.466230000
1	-6.511619000	1.097360000	-5.204021000
1	-7.063812000	3.351852000	-4.299179000
1	-5.395237000	3.841513000	-4.494970000
1	-5.586100000	3.425765000	-6.961956000
1	-7.282499000	3.051456000	-6.752565000
1	-5.965824000	5.695108000	-6.007413000
1	-7.671477000	5.346331000	-5.856906000
1	-7.949795000	5.237715000	-8.259739000
1	-6.224596000	5.107564000	-8.516302000
1	-4.837170000	-2.801378000	-3.202413000
1	-5.638555000	-2.763100000	-1.611265000
1	-4.210827000	-3.796338000	-1.872331000
1	-2.855966000	-1.088542000	0.055983000
1	-3.060010000	-2.854609000	-0.073587000
1	-4.465244000	-1.823684000	0.294914000
1	-2.697440000	-1.795819000	-3.497275000
1	-1.889302000	-0.993458000	-2.119727000
1	-2.033214000	-2.767991000	-2.171262000

1	-5.868359000	7.396676000	-7.743438000
1	-6.931604000	7.334497000	-9.131086000
1	-8.908097000	7.570466000	-7.647449000
1	-7.861391000	7.675543000	-6.249784000
1	-8.660260000	9.892204000	-7.070881000
1	-7.823203000	9.631301000	-8.585149000
1	-5.653511000	9.535966000	-7.371305000
1	-6.487286000	9.728743000	-5.848107000
1	-6.482026000	11.773549000	-8.107724000
1	-7.336750000	11.946150000	-6.591441000
1	-5.146543000	11.793945000	-5.365902000
1	-5.312987000	13.200729000	-6.391440000
1	-3.960482000	12.107109000	-8.152802000
1	-3.777636000	10.684045000	-7.154940000
1	-2.777381000	13.471824000	-6.403718000
1	-1.773804000	12.135314000	-6.964118000
1	-2.600192000	12.027209000	-5.411551000
16	-1.367342000	1.813382000	-0.529224000
8	-1.844531000	2.014736000	-2.065029000
8	-0.772176000	0.476146000	-0.465132000
8	-0.409385000	2.885899000	-0.255277000
8	-2.631941000	1.938310000	0.190385000
6	-2.414060000	4.125356000	-5.850716000
6	-2.997804000	5.526898000	-5.935929000
6	-1.899815000	3.749452000	-4.471907000
6	-1.979348000	6.643672000	-5.778079000
6	-1.441131000	2.304880000	-4.399566000
6	-2.569555000	8.037225000	-5.944460000
6	-0.833365000	1.920386000	-3.075765000
6	-3.555660000	8.420817000	-4.855431000
1	-3.182798000	3.405663000	-6.143626000
1	-1.605091000	4.020945000	-6.580633000
1	-3.497881000	5.646481000	-6.901532000
1	-3.776677000	5.625928000	-5.176918000
1	-2.689308000	3.920187000	-3.735985000
1	-1.067602000	4.398963000	-4.189804000
1	-1.185354000	6.501193000	-6.516694000
1	-1.500927000	6.578341000	-4.797268000
1	-0.680459000	2.122192000	-5.162370000
1	-2.273575000	1.633252000	-4.622679000
1	-1.755811000	8.766135000	-5.959602000
1	-3.058927000	8.106448000	-6.920612000
1	-0.457155000	0.897488000	-3.100965000
1	-0.012409000	2.591414000	-2.818602000
1	-3.098814000	8.327621000	-3.868424000
1	-4.442673000	7.787409000	-4.871533000
1	-3.889190000	9.451741000	-4.972539000
**3-CTA$^+$/(C$_8$-OSO$_3$)$^-$ 1:1 ΔG=1.87**			
7	1.674273000	2.426348000	3.885634000
6	1.502087000	0.989751000	3.472510000
6	2.543261000	0.025295000	3.989347000

6	2.229098000	-1.373478000	3.477745000
6	3.165605000	-2.444763000	4.014447000
6	4.592981000	-2.334613000	3.508681000
6	5.485550000	-3.458184000	4.010341000
6	6.846073000	-3.523631000	3.332178000
6	7.683003000	-2.265937000	3.479091000
6	0.531152000	3.197112000	3.327522000
6	2.937442000	2.989605000	3.341922000
6	1.664390000	2.562293000	5.365121000
6	9.043540000	-2.368578000	2.814066000
6	9.820944000	-1.065906000	2.861953000
6	11.154781000	-1.129743000	2.139243000
6	11.901909000	0.195773000	2.099213000
6	11.236186000	1.256849000	1.238913000
6	12.015444000	2.557247000	1.177687000
6	11.340296000	3.615767000	0.324582000
6	12.118624000	4.916520000	0.266693000
1	1.495542000	0.997158000	2.384051000
1	0.510844000	0.702553000	3.818397000
1	3.537246000	0.325343000	3.663333000
1	2.542051000	0.006795000	5.079362000
1	2.263210000	-1.377050000	2.384424000
1	1.203270000	-1.626815000	3.755413000
1	3.159349000	-2.415788000	5.108347000
1	2.768525000	-3.423484000	3.733335000
1	4.581070000	-2.341653000	2.414969000
1	5.016173000	-1.373635000	3.802764000
1	5.620569000	-3.354598000	5.091526000
1	4.972946000	-4.412054000	3.857657000
1	6.703631000	-3.729245000	2.266787000
1	7.403391000	-4.374215000	3.734357000
1	7.810929000	-2.034315000	4.541617000
1	7.148666000	-1.418764000	3.044401000
1	-0.389296000	2.803537000	3.746554000
1	0.532788000	3.084901000	2.248088000
1	0.653808000	4.240580000	3.599061000
1	2.980642000	4.041533000	3.606738000
1	2.928695000	2.871469000	2.263162000
1	3.781499000	2.462832000	3.769452000
1	0.764239000	2.093135000	5.750128000
1	2.546914000	2.086114000	5.775374000
1	1.671756000	3.620015000	5.608674000
1	9.626108000	-3.167583000	3.283132000
1	8.907354000	-2.662501000	1.768469000
1	9.201163000	-0.281055000	2.425576000
1	9.990893000	-0.779460000	3.904783000
1	10.992804000	-1.479204000	1.114428000
1	11.783828000	-1.883263000	2.620932000
1	12.913599000	0.024149000	1.721593000
1	12.018404000	0.576663000	3.118913000
1	11.108656000	0.865995000	0.224568000

1	10.231198000	1.466597000	1.612372000
1	13.018860000	2.363324000	0.785946000
1	12.153506000	2.945915000	2.191518000
1	10.337542000	3.805745000	0.717212000
1	11.202227000	3.225774000	-0.687612000
1	13.113638000	4.758366000	-0.152070000
1	12.243446000	5.341568000	1.263829000
1	11.612097000	5.658663000	-0.350416000
16	5.893362000	1.599183000	1.573564000
8	7.203449000	0.827450000	1.021222000
8	6.023447000	2.991413000	1.134451000
8	4.741319000	0.916669000	0.980299000
8	6.020778000	1.412379000	3.016524000
6	6.092798000	-1.609437000	-1.736357000
6	5.276270000	-2.893823000	-1.737131000
6	7.233881000	-1.606273000	-0.731195000
6	4.529429000	-3.142482000	-0.437118000
6	8.129108000	-0.380341000	-0.820729000
6	3.584827000	-4.333876000	-0.473741000
6	7.469590000	0.905224000	-0.386031000
6	4.286779000	-5.669378000	-0.644738000
1	6.507031000	-1.451054000	-2.735946000
1	5.423724000	-0.766835000	-1.544529000
1	4.552726000	-2.853191000	-2.556487000
1	5.938773000	-3.735567000	-1.955006000
1	7.846432000	-2.497094000	-0.893059000
1	6.842706000	-1.686093000	0.284517000
1	3.959169000	-2.243560000	-0.184862000
1	5.244049000	-3.288754000	0.375331000
1	8.468591000	-0.240917000	-1.850010000
1	9.024029000	-0.529299000	-0.214679000
1	3.009056000	-4.352315000	0.454971000
1	2.859028000	-4.195792000	-1.280206000
1	8.125266000	1.758907000	-0.559414000
1	6.537306000	1.072362000	-0.923179000
1	5.023477000	-5.823941000	0.145769000
1	4.807404000	-5.732932000	-1.599924000
1	3.575665000	-6.494557000	-0.602423000
**4-CTA$^+$/(C$_8$-OSO$_3$)$^-$ 1:1 ΔG=2.00**			
7	1.493678000	-4.161118000	-1.099715000
6	2.993638000	-4.242600000	-0.997548000
6	3.524918000	-5.098693000	0.128288000
6	5.045729000	-5.174948000	0.082806000
6	5.750493000	-3.838196000	0.244820000
6	7.250248000	-3.999039000	0.423700000
6	8.017937000	-2.687846000	0.487889000
6	7.727712000	-1.850102000	1.721201000
6	8.540352000	-0.566780000	1.758804000
6	1.173361000	-3.228368000	-2.215020000
6	0.902005000	-3.625382000	0.154516000
6	0.899242000	-5.487783000	-1.403100000

6	8.415794000	0.219130000	3.054877000
6	7.008390000	0.694359000	3.367432000
6	6.932838000	1.552113000	4.617081000
6	5.516516000	1.990813000	4.946271000
6	5.401353000	2.870627000	6.181653000
6	5.715757000	2.158471000	7.485823000
6	5.534168000	3.042601000	8.706073000
6	5.845750000	2.325253000	10.005833000
1	3.329717000	-3.213310000	-0.902401000
1	3.332727000	-4.625485000	-1.959105000
1	3.225010000	-4.690137000	1.092772000
1	3.123909000	-6.109350000	0.063198000
1	5.360976000	-5.643716000	-0.853374000
1	5.366895000	-5.844767000	0.883469000
1	5.568553000	-3.206899000	-0.628306000
1	5.322922000	-3.311006000	1.100722000
1	7.444885000	-4.577772000	1.332236000
1	7.638257000	-4.596005000	-0.406182000
1	9.089283000	-2.903568000	0.456418000
1	7.800544000	-2.098699000	-0.408811000
1	7.944211000	-2.442872000	2.616597000
1	6.663723000	-1.611672000	1.763258000
1	8.243459000	0.069988000	0.919385000
1	9.593651000	-0.811547000	1.596115000
1	1.609251000	-3.622871000	-3.127513000
1	1.591563000	-2.254934000	-1.981329000
1	0.094207000	-3.165557000	-2.311752000
1	-0.160226000	-3.475130000	-0.010543000
1	1.384775000	-2.682307000	0.388444000
1	1.051983000	-4.340895000	0.954385000
1	-0.166837000	-5.354136000	-1.557317000
1	1.363971000	-5.877780000	-2.303328000
1	1.067567000	-6.157755000	-0.568533000
1	8.776592000	-0.396781000	3.884869000
1	9.080637000	1.086106000	3.008196000
1	6.614642000	1.255395000	2.513454000
1	6.351188000	-0.167780000	3.496373000
1	7.564762000	2.437179000	4.491974000
1	7.354648000	0.990758000	5.454302000
1	4.887368000	1.105067000	5.077306000
1	5.108090000	2.527946000	4.085652000
1	6.062300000	3.736421000	6.073030000
1	4.384942000	3.269758000	6.239370000
1	6.741806000	1.782379000	7.471513000
1	5.070010000	1.279194000	7.578620000
1	4.506663000	3.415666000	8.730495000
1	6.175702000	3.922815000	8.610251000
1	5.198781000	1.456434000	10.136743000
1	5.704977000	2.978014000	10.867404000
1	6.878327000	1.972976000	10.019865000
16	2.858681000	0.005253000	-0.270774000

8	3.178006000	-0.770611000	1.116185000
8	3.327970000	1.379368000	-0.084885000
8	3.638978000	-0.771269000	-1.229950000
8	1.410019000	-0.089858000	-0.461822000
6	3.524063000	-3.213220000	4.645961000
6	4.990815000	-3.448629000	4.322419000
6	2.776610000	-2.488751000	3.534636000
6	5.794533000	-4.069987000	5.455417000
6	3.092794000	-1.003927000	3.484911000
6	5.888018000	-3.229719000	6.722901000
6	2.515222000	-0.288005000	2.292234000
6	6.467034000	-1.843596000	6.500686000
1	3.049045000	-4.178677000	4.838619000
1	3.427376000	-2.637208000	5.569951000
1	5.050796000	-4.105615000	3.450502000
1	5.461724000	-2.511720000	4.019451000
1	1.699150000	-2.612093000	3.668971000
1	3.022916000	-2.949703000	2.576054000
1	6.807541000	-4.267785000	5.093259000
1	5.365499000	-5.043895000	5.707851000
1	4.170251000	-0.839306000	3.492130000
1	2.698664000	-0.518915000	4.380803000
1	4.902430000	-3.140430000	7.184730000
1	6.507437000	-3.766942000	7.444622000
1	1.444570000	-0.476330000	2.194831000
1	2.678823000	0.786538000	2.373511000
1	5.812432000	-1.226929000	5.884496000
1	7.435288000	-1.898539000	5.998835000
1	6.611083000	-1.322127000	7.447229000
**5-CTA$^+$/(C_8-OSO_3)$^-$ 1:1 ΔG=2.31**			
7	-3.200919000	-2.216168000	2.652912000
6	-3.801813000	-1.802541000	1.337130000
6	-3.975158000	-2.896817000	0.312152000
6	-4.602158000	-2.307173000	-0.941065000
6	-4.705967000	-3.314260000	-2.072193000
6	-5.302448000	-2.738788000	-3.348681000
6	-6.799134000	-2.483281000	-3.274639000
6	-7.357634000	-1.729436000	-4.472290000
6	-7.201762000	-2.444328000	-5.803394000
6	-3.061737000	-0.988508000	3.482343000
6	-1.852673000	-2.813553000	2.462698000
6	-4.079329000	-3.180690000	3.361929000
6	-7.867862000	-1.693077000	-6.943315000
6	-7.748048000	-2.359834000	-8.306086000
6	-6.326692000	-2.418575000	-8.839464000
6	-6.204179000	-3.012997000	-10.235208000
6	-6.937950000	-2.253368000	-11.334916000
6	-6.599594000	-0.772789000	-11.437701000
6	-5.128446000	-0.478426000	-11.673837000
6	-4.857704000	0.998098000	-11.895980000
1	-3.146790000	-1.025855000	0.950180000

1	-4.762141000	-1.352856000	1.582849000
1	-3.010851000	-3.333354000	0.054745000
1	-4.611614000	-3.695958000	0.692286000
1	-4.000297000	-1.459327000	-1.276752000
1	-5.588153000	-1.909246000	-0.692885000
1	-3.704710000	-3.696541000	-2.286482000
1	-5.298826000	-4.174257000	-1.746143000
1	-4.790425000	-1.802409000	-3.590377000
1	-5.093519000	-3.421765000	-4.173626000
1	-7.319596000	-3.440653000	-3.171376000
1	-7.028615000	-1.912852000	-2.372393000
1	-6.870477000	-0.750891000	-4.534790000
1	-8.419158000	-1.530407000	-4.300798000
1	-7.633155000	-3.448325000	-5.729888000
1	-6.141690000	-2.580670000	-6.022037000
1	-2.638595000	-1.266579000	4.442204000
1	-4.044582000	-0.548478000	3.617485000
1	-2.404759000	-0.298622000	2.962584000
1	-1.951376000	-3.758294000	1.941112000
1	-1.416355000	-2.979152000	3.442765000
1	-1.250141000	-2.120830000	1.884813000
1	-5.064191000	-2.737296000	3.470985000
1	-4.138003000	-4.098554000	2.789082000
1	-3.646899000	-3.381353000	4.336870000
1	-8.926572000	-1.561465000	-6.703632000
1	-7.441507000	-0.686919000	-7.001314000
1	-8.158916000	-3.373593000	-8.255346000
1	-8.375843000	-1.812639000	-9.012422000
1	-5.705576000	-3.007744000	-8.162254000
1	-5.905446000	-1.410130000	-8.832109000
1	-6.574923000	-4.041752000	-10.218906000
1	-5.144598000	-3.082073000	-10.491257000
1	-6.713632000	-2.733926000	-12.291271000
1	-8.016061000	-2.356694000	-11.195199000
1	-6.927056000	-0.252321000	-10.533267000
1	-7.180591000	-0.341189000	-12.257495000
1	-4.782042000	-1.049545000	-12.540061000
1	-4.539696000	-0.828064000	-10.822944000
1	-3.795708000	1.191780000	-12.047351000
1	-5.184924000	1.587480000	-11.038080000
1	-5.392665000	1.366573000	-12.772643000
16	-0.582396000	-0.272758000	-0.532299000
8	-1.806725000	0.351719000	-1.394210000
8	-0.671786000	-1.724231000	-0.696862000
8	0.642352000	0.299707000	-1.093635000
8	-0.899223000	0.196516000	0.814949000
6	-4.227788000	1.239663000	-5.568652000
6	-4.468706000	0.806748000	-7.003881000
6	-3.120672000	0.450029000	-4.894880000
6	-5.556430000	1.591691000	-7.719957000
6	-2.971948000	0.787639000	-3.423273000

6	-5.177617000	3.022853000	-8.061454000
6	-1.857651000	0.002972000	-2.782430000
6	-6.290521000	3.762613000	-8.780084000
1	-3.981020000	2.303074000	-5.527848000
1	-5.155918000	1.117212000	-5.001922000
1	-3.535645000	0.884764000	-7.571125000
1	-4.735285000	-0.253248000	-7.001328000
1	-2.175669000	0.639007000	-5.411998000
1	-3.320687000	-0.619449000	-5.004331000
1	-6.464529000	1.595492000	-7.108651000
1	-5.818002000	1.074345000	-8.647046000
1	-3.905879000	0.572930000	-2.899247000
1	-2.773967000	1.854795000	-3.302935000
1	-4.912050000	3.566840000	-7.152701000
1	-4.279712000	3.012799000	-8.685857000
1	-2.034162000	-1.070148000	-2.869921000
1	-0.899342000	0.239253000	-3.246711000
1	-6.561597000	3.256425000	-9.707914000
1	-5.997523000	4.782428000	-9.029834000
1	-7.186543000	3.816165000	-8.159696000
**1-CTA^{+}/ (C$_8$-OSO$_3$)$^{-}$ 2:1 ΔG=0.0**			
7	2.822408000	3.609184000	-0.743752000
6	2.894036000	3.095080000	0.669679000
6	4.274584000	3.087201000	1.284553000
6	4.273451000	2.335444000	2.611000000
6	4.010911000	0.838161000	2.515977000
6	4.979271000	0.093869000	1.612286000
6	4.854945000	-1.414336000	1.726903000
6	5.793659000	-2.162446000	0.796531000
6	5.760080000	-3.675587000	0.958920000
6	3.456097000	2.643214000	-1.680220000
6	3.473914000	4.936558000	-0.872697000
6	1.384856000	3.742112000	-1.104135000
6	6.371767000	-4.163336000	2.260946000
6	6.408344000	-5.677734000	2.383195000
6	7.136817000	-6.166632000	3.627214000
6	6.386088000	-5.934768000	4.928223000
6	7.244318000	-6.173535000	6.156613000
6	6.521749000	-5.898175000	7.461747000
6	7.412262000	-6.041790000	8.681985000
6	6.689333000	-5.745199000	9.982176000
1	2.461867000	2.099441000	0.628604000
1	2.228931000	3.729924000	1.252334000
1	5.001455000	2.632524000	0.613406000
1	4.604720000	4.109296000	1.465309000
1	3.538470000	2.789694000	3.280181000
1	5.248228000	2.490533000	3.077996000
1	4.078284000	0.422953000	3.524460000
1	2.987615000	0.646522000	2.184555000
1	4.818985000	0.385729000	0.570617000
1	6.004248000	0.389600000	1.859031000

1	5.041099000	-1.704722000	2.763632000
1	3.825772000	-1.709130000	1.505744000
1	6.817948000	-1.810824000	0.956855000
1	5.541458000	-1.904226000	-0.235434000
1	6.299660000	-4.134852000	0.126286000
1	4.726333000	-4.027094000	0.883250000
1	2.949809000	1.688796000	-1.576613000
1	4.508075000	2.545578000	-1.438903000
1	3.345210000	3.026913000	-2.689606000
1	3.291674000	5.307578000	-1.876269000
1	4.539519000	4.830461000	-0.706575000
1	3.040172000	5.608024000	-0.137799000
1	1.316358000	4.050880000	-2.142298000
1	0.901730000	2.782514000	-0.954603000
1	0.937026000	4.490934000	-0.458573000
1	7.394892000	-3.779800000	2.333651000
1	5.827594000	-3.738302000	3.105676000
1	5.387348000	-6.072603000	2.373790000
1	6.901994000	-6.087326000	1.497808000
1	8.109782000	-5.667102000	3.679945000
1	7.350782000	-7.234204000	3.530440000
1	5.507542000	-6.586234000	4.959190000
1	6.001600000	-4.912917000	4.962548000
1	7.612628000	-7.204248000	6.152757000
1	8.130699000	-5.534038000	6.097957000
1	5.664603000	-6.571942000	7.555479000
1	6.109026000	-4.884440000	7.436598000
1	8.269662000	-5.370887000	8.578160000
1	7.820981000	-7.055524000	8.712780000
1	7.352299000	-5.850386000	10.841102000
1	5.847431000	-6.424207000	10.125153000
1	6.296609000	-4.727058000	9.986496000
7	-2.988794000	0.150736000	3.476633000
6	-1.678465000	0.478450000	4.142665000
6	-1.295418000	-0.416089000	5.297104000
6	0.110281000	-0.084402000	5.772544000
6	0.494254000	-0.905786000	6.990282000
6	1.915899000	-0.687527000	7.483644000
6	2.986066000	-1.087260000	6.481711000
6	4.376674000	-1.170445000	7.083274000
6	5.428654000	-1.598520000	6.077272000
6	-2.911007000	-1.156689000	2.772177000
6	-3.262722000	1.213830000	2.470816000
6	-4.100345000	0.122218000	4.460158000
6	6.811296000	-1.775514000	6.675933000
6	7.835018000	-2.255426000	5.664387000
6	9.195546000	-2.552369000	6.264938000
6	10.178590000	-3.110801000	5.252907000
6	11.526373000	-3.459763000	5.858265000
6	12.540706000	-3.999566000	4.861541000
6	12.178509000	-5.349249000	4.264741000

6	13.255593000	-5.884780000	3.339847000
1	-1.766887000	1.510237000	4.477467000
1	-0.932128000	0.434464000	3.353169000
1	-1.993086000	-0.289850000	6.125118000
1	-1.324076000	-1.465627000	5.003163000
1	0.179058000	0.980363000	6.012910000
1	0.811927000	-0.266860000	4.956438000
1	-0.205187000	-0.680897000	7.799503000
1	0.360910000	-1.966391000	6.758169000
1	2.051941000	0.360054000	7.769066000
1	2.055336000	-1.272434000	8.396896000
1	2.729756000	-2.061291000	6.056616000
1	2.998527000	-0.385308000	5.644547000
1	4.363689000	-1.883320000	7.914208000
1	4.651901000	-0.202794000	7.514320000
1	5.473665000	-0.868039000	5.263733000
1	5.117337000	-2.542872000	5.618879000
1	-3.857385000	-1.326356000	2.268484000
1	-2.733758000	-1.944224000	3.494856000
1	-2.102438000	-1.111905000	2.050475000
1	-2.424779000	1.257300000	1.782004000
1	-3.373583000	2.159285000	2.992390000
1	-4.179865000	0.963906000	1.947022000
1	-3.961983000	-0.714382000	5.134992000
1	-4.100183000	1.058263000	5.010188000
1	-5.031839000	0.006609000	3.915220000
1	7.144896000	-0.831888000	7.118524000
1	6.754952000	-2.495402000	7.498541000
1	7.458875000	-3.160636000	5.180466000
1	7.940173000	-1.509735000	4.869921000
1	9.075598000	-3.271375000	7.081760000
1	9.607201000	-1.643920000	6.715451000
1	10.322222000	-2.385550000	4.445523000
1	9.737505000	-3.997596000	4.791348000
1	11.938645000	-2.567221000	6.336751000
1	11.381791000	-4.193923000	6.657384000
1	13.511800000	-4.087661000	5.356560000
1	12.676435000	-3.273604000	4.053395000
1	11.237984000	-5.274185000	3.715496000
1	12.003152000	-6.061918000	5.075764000
1	14.201303000	-6.007606000	3.870055000
1	13.430086000	-5.201123000	2.507518000
1	12.979096000	-6.853096000	2.922511000
16	0.217232000	-0.016485000	0.346321000
8	0.511387000	-0.853296000	1.703179000
8	1.473197000	-0.004533000	-0.405971000
8	-0.862448000	-0.728477000	-0.339516000
8	-0.168180000	1.285371000	0.883018000
6	2.599282000	-4.653097000	3.999117000
6	1.791097000	-4.716498000	5.282559000
6	1.814264000	-4.187478000	2.783165000

6	2.636205000	-5.064369000	6.493660000
6	1.362650000	-2.738779000	2.876383000
6	1.862079000	-5.035457000	7.798390000
6	0.853874000	-2.235349000	1.551227000
6	2.727368000	-5.350385000	9.003720000
1	3.450614000	-3.980236000	4.144065000
1	3.022938000	-5.639860000	3.795937000
1	1.300775000	-3.756837000	5.458902000
1	0.987415000	-5.450873000	5.170420000
1	0.946725000	-4.835762000	2.630821000
1	2.443676000	-4.304292000	1.897480000
1	3.472279000	-4.360580000	6.560502000
1	3.084510000	-6.053180000	6.356498000
1	2.201584000	-2.113868000	3.190308000
1	0.579450000	-2.621818000	3.626641000
1	1.409018000	-4.047816000	7.922048000
1	1.033055000	-5.746330000	7.743467000
1	1.621875000	-2.330040000	0.782425000
1	-0.031021000	-2.786609000	1.229264000
1	3.546379000	-4.634353000	9.092180000
1	3.168319000	-6.345000000	8.920537000
1	2.152825000	-5.316831000	9.929570000

2-CTA$^+$/ $(C_8\text{-}OSO_3)^-$ 2:1 ΔG=2.65

7	-2.725892000	2.544035000	-2.675438000
6	-3.104960000	3.028347000	-1.301114000
6	-4.567034000	2.894248000	-0.941051000
6	-4.824448000	3.405156000	0.472320000
6	-4.214927000	2.538394000	1.562217000
6	-4.455259000	3.058453000	2.970705000
6	-5.916151000	3.065523000	3.393521000
6	-6.124798000	3.317757000	4.879245000
6	-5.682195000	4.694603000	5.346961000
6	-3.439537000	3.304040000	-3.733017000
6	-3.007901000	1.091519000	-2.823072000
6	-1.262153000	2.759266000	-2.836998000
6	-5.872105000	4.921096000	6.838899000
6	-7.316721000	5.070402000	7.289215000
6	-7.455643000	5.157125000	8.803287000
6	-7.288279000	3.820432000	9.507643000
6	-7.204679000	3.942895000	11.017397000
6	-7.073615000	2.605582000	11.722493000
6	-6.898795000	2.733867000	13.224447000
6	-6.746076000	1.394969000	13.920167000
1	-2.802426000	4.073742000	-1.264151000
1	-2.475976000	2.465564000	-0.617757000
1	-5.180203000	3.465402000	-1.636768000
1	-4.885803000	1.853387000	-1.002719000
1	-5.904202000	3.464099000	0.611184000
1	-4.447412000	4.428081000	0.564735000
1	-4.617520000	1.523875000	1.477141000

1	-3.139444000	2.454173000	1.404428000
1	-3.894702000	2.435088000	3.672908000
1	-4.033833000	4.063455000	3.053910000
1	-6.470199000	3.815048000	2.822524000
1	-6.356997000	2.098207000	3.135572000
1	-5.578927000	2.558473000	5.449665000
1	-7.181396000	3.174740000	5.113524000
1	-6.223874000	5.461617000	4.783442000
1	-4.625096000	4.831078000	5.109179000
1	-3.057085000	2.984393000	-4.697219000
1	-3.250987000	4.362869000	-3.584585000
1	-4.500857000	3.095258000	-3.672052000
1	-4.079163000	0.930540000	-2.800566000
1	-2.523233000	0.565967000	-2.006252000
1	-2.608706000	0.765043000	-3.778229000
1	-0.972533000	2.426568000	-3.828456000
1	-1.051800000	3.817327000	-2.717569000
1	-0.743014000	2.182192000	-2.079336000
1	-5.402452000	4.094363000	7.374680000
1	-5.327921000	5.821204000	7.138042000
1	-7.734610000	5.969842000	6.829350000
1	-7.918394000	4.233660000	6.925237000
1	-8.434576000	5.568969000	9.061374000
1	-6.715962000	5.865991000	9.189376000
1	-6.386847000	3.319696000	9.147629000
1	-8.125821000	3.169343000	9.238969000
1	-6.345473000	4.569946000	11.275993000
1	-8.088988000	4.466842000	11.393463000
1	-6.218864000	2.062619000	11.306957000
1	-7.955670000	1.993469000	11.510201000
1	-7.755301000	3.269936000	13.642369000
1	-6.021513000	3.353381000	13.428468000
1	-5.880570000	0.853725000	13.534809000
1	-7.625654000	0.768743000	13.761907000
1	-6.611507000	1.515350000	14.995328000
7	3.904886000	0.942071000	2.801154000
6	2.882679000	2.010438000	3.081944000
6	3.382807000	3.434573000	2.994886000
6	2.219801000	4.417648000	3.051119000
6	1.430388000	4.381394000	4.348071000
6	0.335351000	5.433595000	4.393792000
6	-0.364914000	5.540182000	5.739765000
6	-1.143184000	4.301308000	6.145079000
6	-1.830203000	4.442500000	7.491471000
6	5.083897000	1.081748000	3.692136000
6	4.340711000	0.980826000	1.380137000
6	3.258127000	-0.374397000	3.056220000
6	-2.605281000	3.196659000	7.878191000
6	-3.292013000	3.263449000	9.232822000
6	-2.351247000	3.175626000	10.422011000
6	-3.087885000	3.136116000	11.748058000

6	-2.174925000	2.967492000	12.947860000
6	-2.926753000	2.898638000	14.263923000
6	-2.023137000	2.715447000	15.469135000
6	-2.791548000	2.640183000	16.775069000
1	2.505853000	1.792644000	4.077971000
1	2.080370000	1.840853000	2.368706000
1	4.072605000	3.645916000	3.812502000
1	3.924433000	3.594118000	2.063494000
1	1.548851000	4.228999000	2.209462000
1	2.621364000	5.422783000	2.907526000
1	0.988945000	3.392840000	4.485083000
1	2.114309000	4.534396000	5.189162000
1	0.770933000	6.404955000	4.145047000
1	-0.402491000	5.220062000	3.613943000
1	0.378338000	5.765469000	6.511076000
1	-1.048772000	6.393019000	5.718125000
1	-0.476271000	3.436237000	6.182428000
1	-1.890405000	4.077208000	5.375920000
1	-2.511715000	5.298970000	7.467036000
1	-1.078749000	4.669555000	8.252412000
1	5.737814000	0.231406000	3.526575000
1	4.739403000	1.097979000	4.721684000
1	5.606857000	2.000378000	3.452632000
1	3.461133000	0.883342000	0.751920000
1	5.020296000	0.151195000	1.212944000
1	4.852062000	1.915587000	1.184585000
1	2.382970000	-0.454265000	2.419844000
1	3.972204000	-1.158631000	2.825677000
1	2.971479000	-0.422431000	4.101967000
1	-3.353402000	3.003143000	7.104907000
1	-1.929963000	2.336502000	7.863217000
1	-3.880572000	4.183665000	9.301588000
1	-4.010612000	2.442578000	9.303490000
1	-1.656697000	4.019805000	10.418970000
1	-1.738554000	2.273904000	10.324693000
1	-3.810045000	2.314222000	11.731294000
1	-3.676381000	4.051644000	11.864523000
1	-1.583266000	2.054903000	12.823434000
1	-1.458422000	3.794075000	12.980691000
1	-3.645110000	2.074049000	14.225198000
1	-3.518042000	3.810683000	14.392475000
1	-1.307585000	3.541028000	15.511644000
1	-1.431375000	1.805271000	15.339295000
1	-2.123409000	2.505814000	17.625776000
1	-3.493598000	1.804873000	16.766695000
1	-3.366224000	3.552368000	16.942829000
16	-0.232470000	0.912297000	0.823589000
8	-0.197423000	0.299674000	2.323050000
8	1.078830000	0.517196000	0.315674000
8	-1.357830000	0.268771000	0.143397000
8	-0.402060000	2.357110000	0.979438000

6	-2.049495000	-0.481799000	6.801265000
6	-3.283056000	-0.454915000	7.685794000
6	-2.310743000	0.021212000	5.394047000
6	-2.978007000	-0.759919000	9.140210000
6	-1.087139000	-0.067184000	4.500374000
6	-4.203148000	-0.746960000	10.036005000
6	-1.354370000	0.517583000	3.138268000
6	-3.861708000	-0.959772000	11.498608000
1	-1.263530000	0.127798000	7.256654000
1	-1.655736000	-1.501646000	6.758407000
1	-3.754973000	0.529258000	7.619290000
1	-4.017271000	-1.171582000	7.305617000
1	-2.652890000	1.058806000	5.444432000
1	-3.128247000	-0.551511000	4.946828000
1	-2.255926000	-0.026570000	9.512474000
1	-2.486512000	-1.735065000	9.214392000
1	-0.784823000	-1.110624000	4.388996000
1	-0.249595000	0.463523000	4.959752000
1	-4.723497000	0.207532000	9.918610000
1	-4.902748000	-1.518470000	9.703323000
1	-2.214454000	0.038350000	2.668640000
1	-1.544345000	1.589014000	3.206593000
1	-3.191697000	-0.175457000	11.855458000
1	-3.360865000	-1.917529000	11.649015000
1	-4.753480000	-0.946771000	12.124909000

3-CTA$^+$/ (C_8-OSO_3)$^-$ 2:1 ΔG=2.68

7	-0.315075000	-2.641882000	-3.961936000
6	-0.774046000	-3.350786000	-2.715346000
6	-2.263453000	-3.584452000	-2.607090000
6	-2.590394000	-4.457722000	-1.402116000
6	-2.214167000	-3.855882000	-0.059146000
6	-2.687218000	-4.697924000	1.112055000
6	-2.259613000	-4.134690000	2.454048000
6	-2.757016000	-4.941390000	3.641369000
6	-2.345408000	-4.352537000	4.983344000
6	1.157822000	-2.457644000	-3.849996000
6	-0.953556000	-1.304736000	-4.081206000
6	-0.604747000	-3.441816000	-5.179108000
6	-0.877236000	-4.544468000	5.329483000
6	-0.440642000	-3.701546000	6.512985000
6	1.024356000	-3.856605000	6.875675000
6	1.463562000	-2.898797000	7.968243000
6	2.938206000	-2.997858000	8.311644000
6	3.370893000	-1.999392000	9.369292000
6	4.850411000	-2.064220000	9.699784000
6	5.255058000	-1.077925000	10.779251000
1	-0.419530000	-2.736032000	-1.892773000
1	-0.236624000	-4.297670000	-2.703913000
1	-2.790336000	-2.634881000	-2.512893000
1	-2.640527000	-4.078976000	-3.501065000

1	-2.099761000	-5.428347000	-1.515996000
1	-3.664444000	-4.654744000	-1.414001000
1	-1.130184000	-3.735067000	0.010354000
1	-2.639112000	-2.850316000	0.020169000
1	-3.777744000	-4.781755000	1.080264000
1	-2.299056000	-5.715512000	1.005395000
1	-1.169431000	-4.068872000	2.476898000
1	-2.627453000	-3.107960000	2.546805000
1	-2.395198000	-5.971593000	3.564297000
1	-3.847672000	-4.997014000	3.592141000
1	-2.953981000	-4.789232000	5.779364000
1	-2.574815000	-3.282799000	4.978899000
1	1.509499000	-1.950793000	-4.742879000
1	1.621018000	-3.435782000	-3.766818000
1	1.368927000	-1.861009000	-2.967807000
1	-2.018969000	-1.431063000	-4.236208000
1	-0.515308000	-0.797252000	-4.935015000
1	-0.761943000	-0.752629000	-3.166704000
1	-1.676755000	-3.512158000	-5.319035000
1	-0.157469000	-2.937721000	-6.029834000
1	-0.170977000	-4.429413000	-5.056835000
1	-0.688699000	-5.602482000	5.535596000
1	-0.255024000	-4.286214000	4.470409000
1	-0.634234000	-2.649977000	6.281989000
1	-1.060187000	-3.938196000	7.383903000
1	1.634136000	-3.685751000	5.982943000
1	1.221580000	-4.886719000	7.187889000
1	0.867284000	-3.076088000	8.869133000
1	1.238732000	-1.874369000	7.653306000
1	3.165613000	-4.012144000	8.654726000
1	3.530372000	-2.842429000	7.404124000
1	2.791544000	-2.166049000	10.281891000
1	3.122609000	-0.987816000	9.032303000
1	5.431184000	-1.875247000	8.792824000
1	5.104988000	-3.078384000	10.018185000
1	5.040287000	-0.052931000	10.472734000
1	6.320056000	-1.142570000	11.002479000
1	4.707517000	-1.267330000	11.704045000
7	-3.785271000	1.109041000	-0.309488000
6	-3.667110000	0.239991000	0.912544000
6	-3.844498000	0.940008000	2.239222000
6	-3.563229000	-0.037404000	3.368628000
6	-3.638275000	0.603087000	4.742003000
6	-3.224520000	-0.355149000	5.842303000
6	-3.087606000	0.287462000	7.209837000
6	-2.544919000	-0.682749000	8.241862000
6	-2.283982000	-0.075389000	9.606873000
6	-3.647639000	0.225338000	-1.499412000
6	-2.696785000	2.122309000	-0.347885000
6	-5.104404000	1.788042000	-0.360756000
6	-1.671933000	-1.078665000	10.567462000

6	-1.292064000	-0.490443000	11.914294000
6	-0.744978000	-1.511606000	12.901458000
6	0.532981000	-2.194848000	12.444813000
6	1.102261000	-3.143807000	13.484794000
6	2.369269000	-3.864631000	13.048065000
6	2.168848000	-4.861631000	11.918629000
6	3.432250000	-5.636824000	11.594674000
1	-2.680846000	-0.214510000	0.847229000
1	-4.412920000	-0.543843000	0.792897000
1	-3.159130000	1.782427000	2.327004000
1	-4.857805000	1.329227000	2.339239000
1	-2.563320000	-0.456841000	3.232981000
1	-4.260264000	-0.877997000	3.312217000
1	-2.980838000	1.476470000	4.762598000
1	-4.650416000	0.974133000	4.926388000
1	-3.937114000	-1.183562000	5.895785000
1	-2.262834000	-0.800734000	5.572184000
1	-2.412908000	1.145683000	7.134819000
1	-4.053845000	0.682214000	7.537668000
1	-1.608327000	-1.103076000	7.864665000
1	-3.233857000	-1.527012000	8.345529000
1	-3.214623000	0.320137000	10.024968000
1	-1.608101000	0.778576000	9.497188000
1	-2.680834000	-0.263924000	-1.448177000
1	-3.717328000	0.837342000	-2.393320000
1	-4.449435000	-0.506040000	-1.481231000
1	-2.775449000	2.663225000	-1.285781000
1	-1.743431000	1.606798000	-0.286822000
1	-2.816179000	2.809490000	0.481387000
1	-5.883514000	1.035883000	-0.285042000
1	-5.176548000	2.493096000	0.458939000
1	-5.179194000	2.314392000	-1.307061000
1	-2.371214000	-1.907310000	10.719640000
1	-0.787936000	-1.510689000	10.093602000
1	-0.549728000	0.299829000	11.763892000
1	-2.168978000	-0.005838000	12.352435000
1	-1.508612000	-2.272378000	13.092624000
1	-0.561167000	-1.017567000	13.859601000
1	1.282228000	-1.434050000	12.202521000
1	0.342980000	-2.740617000	11.520041000
1	1.308860000	-2.581480000	14.399529000
1	0.342189000	-3.886153000	13.749364000
1	2.788092000	-4.394849000	13.907823000
1	3.122341000	-3.128468000	12.748249000
1	1.827168000	-4.346221000	11.018972000
1	1.372351000	-5.558211000	12.195417000
1	4.237200000	-4.963528000	11.296774000
1	3.270669000	-6.341095000	10.778441000
1	3.778214000	-6.202854000	12.461081000
16	0.893577000	-0.086058000	-0.234290000
8	1.135462000	-0.963039000	1.105746000

8	0.866006000	1.313385000	0.193746000
8	2.052882000	-0.443942000	-1.045951000
8	-0.387296000	-0.538158000	-0.783438000
6	1.562965000	0.625513000	4.264029000
6	0.352355000	0.987372000	5.107926000
6	1.734207000	-0.866758000	4.020009000
6	0.334573000	2.442353000	5.557996000
6	0.556008000	-1.561644000	3.352505000
6	0.391121000	3.472126000	4.436747000
6	0.075179000	-0.934323000	2.070213000
6	-0.761700000	3.378937000	3.453491000
1	1.514045000	1.147401000	3.305737000
1	2.464711000	0.996492000	4.759227000
1	-0.563429000	0.762774000	4.557595000
1	0.329540000	0.347877000	5.995834000
1	1.920744000	-1.365395000	4.974743000
1	2.629559000	-1.022585000	3.415169000
1	1.180416000	2.612240000	6.229933000
1	-0.567482000	2.616655000	6.151224000
1	-0.303717000	-1.586096000	4.025424000
1	0.817626000	-2.602704000	3.157570000
1	0.399096000	4.468103000	4.885375000
1	1.336612000	3.379523000	3.897983000
1	-0.770699000	-1.492404000	1.670645000
1	-0.233535000	0.099436000	2.225528000
1	-0.764813000	2.424668000	2.926298000
1	-0.702563000	4.167964000	2.703272000
1	-1.720548000	3.476947000	3.965144000
**4-CTA$^+$/ (C_8-OSO_3)$^-$ 2:1 ΔG=2.75**			
7	-1.005192000	-1.039248000	-3.212834000
6	0.388128000	-1.531390000	-2.928673000
6	1.412807000	-1.274656000	-4.007986000
6	2.793341000	-1.634854000	-3.484934000
6	3.881377000	-1.483057000	-4.531986000
6	5.269659000	-1.579152000	-3.924863000
6	6.413728000	-1.393768000	-4.909034000
6	6.480309000	-0.002625000	-5.514828000
6	7.708419000	0.227411000	-6.376082000
6	-1.505057000	-1.555589000	-4.511792000
6	-1.049099000	0.446497000	-3.216163000
6	-1.887900000	-1.535737000	-2.121110000
6	7.701036000	1.592852000	-7.041136000
6	8.919540000	1.890440000	-7.901081000
6	10.212775000	2.026736000	-7.116433000
6	11.373646000	2.508246000	-7.967359000
6	12.684769000	2.651694000	-7.206156000
6	12.643614000	3.596774000	-6.012043000
6	12.217568000	5.015501000	-6.346072000
6	12.285274000	5.940037000	-5.144910000
1	0.294223000	-2.601093000	-2.749677000
1	0.676840000	-1.055078000	-1.994237000

1	1.191078000	-1.867701000	-4.895585000
1	1.416434000	-0.226284000	-4.307936000
1	2.790047000	-2.660485000	-3.106650000
1	3.022220000	-0.992637000	-2.630414000
1	3.760214000	-2.247691000	-5.304753000
1	3.759420000	-0.521578000	-5.033597000
1	5.357998000	-0.823877000	-3.137713000
1	5.371472000	-2.548299000	-3.428924000
1	7.355796000	-1.597876000	-4.392202000
1	6.336742000	-2.137046000	-5.708584000
1	6.466839000	0.737999000	-4.708807000
1	5.586939000	0.185430000	-6.115442000
1	7.766105000	-0.547483000	-7.147294000
1	8.602947000	0.112931000	-5.758605000
1	-1.406271000	-2.636784000	-4.515478000
1	-0.925699000	-1.118871000	-5.317002000
1	-2.548085000	-1.272374000	-4.611429000
1	-0.428813000	0.824281000	-4.020414000
1	-0.687945000	0.805047000	-2.257315000
1	-2.078542000	0.754563000	-3.370095000
1	-1.897508000	-2.620569000	-2.153480000
1	-1.487539000	-1.187984000	-1.174099000
1	-2.887711000	-1.146340000	-2.283540000
1	6.801073000	1.673712000	-7.656916000
1	7.612582000	2.366031000	-6.271312000
1	9.030522000	1.111745000	-8.662382000
1	8.744693000	2.823395000	-8.444322000
1	10.478959000	1.072438000	-6.654268000
1	10.046993000	2.727307000	-6.295334000
1	11.106418000	3.463435000	-8.426960000
1	11.524906000	1.810201000	-8.795388000
1	13.003132000	1.664647000	-6.859428000
1	13.457285000	2.994166000	-7.900269000
1	13.638714000	3.625201000	-5.559397000
1	11.977522000	3.197750000	-5.242143000
1	11.198737000	5.013179000	-6.740143000
1	12.853195000	5.404354000	-7.146895000
1	11.956664000	6.948613000	-5.396681000
1	13.303904000	6.006683000	-4.759204000
1	11.648750000	5.575614000	-4.337098000
7	2.895155000	-3.505389000	1.282059000
6	3.907774000	-2.392761000	1.296772000
6	5.049069000	-2.526394000	0.316532000
6	5.993150000	-1.344503000	0.471557000
6	7.193061000	-1.440640000	-0.454300000
6	8.200054000	-0.313256000	-0.288631000
6	7.667737000	1.065799000	-0.645374000
6	8.719040000	2.162940000	-0.525302000
6	9.751631000	2.182546000	-1.647372000
6	1.890084000	-3.213420000	2.340719000
6	3.531736000	-4.814318000	1.575921000

6	2.197884000	-3.573009000	-0.028628000
6	9.245611000	2.744953000	-2.969759000
6	9.006316000	4.245163000	-2.943473000
6	8.565599000	4.808400000	-4.281258000
6	8.358763000	6.311118000	-4.261360000
6	7.793885000	6.853246000	-5.560546000
6	7.603507000	8.358023000	-5.552993000
6	6.989903000	8.894044000	-6.833068000
6	6.803871000	10.399258000	-6.815052000
1	4.287811000	-2.357663000	2.316092000
1	3.345660000	-1.479991000	1.109393000
1	5.603298000	-3.448352000	0.492088000
1	4.679388000	-2.554665000	-0.709031000
1	6.340882000	-1.290241000	1.507135000
1	5.440382000	-0.423473000	0.279853000
1	7.697394000	-2.393563000	-0.274090000
1	6.847958000	-1.468220000	-1.490582000
1	8.556169000	-0.297333000	0.746376000
1	9.072402000	-0.538007000	-0.905767000
1	7.255954000	1.046387000	-1.659378000
1	6.832127000	1.311101000	0.012260000
1	9.233644000	2.041987000	0.431810000
1	8.223585000	3.134243000	-0.474680000
1	10.131310000	1.171260000	-1.807984000
1	10.612716000	2.776624000	-1.329258000
1	2.398655000	-3.179608000	3.299010000
1	1.149403000	-4.006614000	2.336503000
1	1.425336000	-2.258346000	2.120457000
1	4.193610000	-5.083615000	0.761688000
1	2.747795000	-5.558198000	1.674402000
1	4.086511000	-4.727824000	2.505035000
1	1.747371000	-2.606193000	-0.228133000
1	2.912314000	-3.834189000	-0.800220000
1	1.430815000	-4.338587000	0.036404000
1	9.979614000	2.523778000	-3.749086000
1	8.325115000	2.234994000	-3.269862000
1	8.250418000	4.491951000	-2.193168000
1	9.926117000	4.746668000	-2.625350000
1	7.637180000	4.319032000	-4.588553000
1	9.307200000	4.558397000	-5.045264000
1	9.310766000	6.804380000	-4.042959000
1	7.685531000	6.575218000	-3.439979000
1	8.454714000	6.575210000	-6.387897000
1	6.832869000	6.368869000	-5.761791000
1	8.569011000	8.844758000	-5.382937000
1	6.968335000	8.637153000	-4.706481000
1	6.024455000	8.407520000	-6.997620000
1	7.621458000	8.612452000	-7.679982000
1	7.759151000	10.909625000	-6.683138000
1	6.151486000	10.702037000	-5.994551000
1	6.359839000	10.758721000	-7.743398000

16	0.843950000	0.491316000	0.923287000
8	2.063304000	0.580788000	-0.139370000
8	0.170783000	-0.734058000	0.499283000
8	1.465822000	0.405166000	2.245717000
8	0.050162000	1.706529000	0.733785000
6	3.731975000	2.403733000	-3.687405000
6	4.989850000	3.262730000	-3.670750000
6	2.964307000	2.345356000	-2.375549000
6	4.778932000	4.722808000	-3.301819000
6	3.794163000	1.827879000	-1.214607000
6	3.904663000	5.490687000	-4.277698000
6	3.005697000	1.644805000	0.055331000
6	3.850642000	6.974678000	-3.966747000
1	3.059570000	2.752121000	-4.474363000
1	4.019264000	1.387966000	-3.965721000
1	5.720940000	2.826017000	-2.985532000
1	5.449106000	3.214885000	-4.661934000
1	2.095453000	1.697698000	-2.510332000
1	2.571552000	3.333710000	-2.121965000
1	5.754705000	5.211568000	-3.250278000
1	4.353101000	4.800183000	-2.297342000
1	4.604147000	2.522604000	-0.986999000
1	4.266598000	0.879753000	-1.484201000
1	4.289129000	5.343899000	-5.291343000
1	2.892120000	5.081190000	-4.272514000
1	3.656483000	1.380675000	0.889009000
1	2.465790000	2.558966000	0.306351000
1	4.847005000	7.417451000	-4.013825000
1	3.457457000	7.150917000	-2.964169000
1	3.214459000	7.509416000	-4.672258000
**5-CTA$^+$/ (C_8-OSO_3)$^-$ 2:1 ΔG=2.90**			
7	-1.180643000	-1.972393000	-4.369462000
6	-0.142397000	-3.022572000	-4.659886000
6	1.047619000	-3.044087000	-3.730752000
6	1.973031000	-4.186813000	-4.124224000
6	3.294546000	-4.191282000	-3.372204000
6	3.159880000	-4.375861000	-1.870603000
6	4.489653000	-4.363881000	-1.130372000
6	5.447449000	-5.494100000	-1.489342000
6	4.888387000	-6.896409000	-1.291966000
6	-0.580922000	-0.613111000	-4.361347000
6	-1.845111000	-2.225646000	-3.063961000
6	-2.203990000	-2.039845000	-5.446823000
6	4.463251000	-7.189229000	0.137750000
6	4.089136000	-8.641772000	0.391458000
6	2.836213000	-9.095892000	-0.336158000
6	2.421025000	-10.512681000	0.013820000
6	1.113759000	-10.931461000	-0.633493000
6	0.671504000	-12.328808000	-0.242806000
6	-0.632882000	-12.752625000	-0.892405000
6	-1.072635000	-14.144996000	-0.482216000

1	0.180067000	-2.841786000	-5.683606000
1	-0.670682000	-3.974217000	-4.631717000
1	1.598705000	-2.105292000	-3.796333000
1	0.726538000	-3.163594000	-2.697998000
1	2.179480000	-4.123043000	-5.195288000
1	1.459780000	-5.138234000	-3.965210000
1	3.916627000	-4.987783000	-3.784596000
1	3.824478000	-3.254588000	-3.571903000
1	2.536262000	-3.579598000	-1.461116000
1	2.625992000	-5.308835000	-1.666444000
1	4.989965000	-3.409983000	-1.320052000
1	4.293021000	-4.388622000	-0.056838000
1	6.348801000	-5.385370000	-0.879737000
1	5.772821000	-5.385448000	-2.526184000
1	5.653312000	-7.620288000	-1.587355000
1	4.047344000	-7.054802000	-1.970364000
1	-0.031202000	-0.470052000	-5.286506000
1	0.079272000	-0.518650000	-3.507304000
1	-1.385669000	0.111108000	-4.285796000
1	-2.277653000	-3.220816000	-3.088624000
1	-2.626105000	-1.482676000	-2.933471000
1	-1.117792000	-2.140364000	-2.264540000
1	-2.624458000	-3.040286000	-5.460425000
1	-2.977338000	-1.308596000	-5.235198000
1	-1.724111000	-1.818571000	-6.394780000
1	5.279829000	-6.909186000	0.809778000
1	3.614330000	-6.555548000	0.408458000
1	4.927859000	-9.287779000	0.112796000
1	3.938530000	-8.784577000	1.465114000
1	2.980818000	-9.022153000	-1.416774000
1	2.017555000	-8.413009000	-0.091242000
1	2.326635000	-10.602199000	1.100369000
1	3.212635000	-11.208777000	-0.280402000
1	0.331857000	-10.216691000	-0.357576000
1	1.209121000	-10.870217000	-1.722338000
1	0.566079000	-12.382028000	0.845524000
1	1.455053000	-13.045634000	-0.507821000
1	-0.523615000	-12.708295000	-1.978910000
1	-1.413925000	-12.030907000	-0.636447000
1	-1.223661000	-14.204978000	0.596854000
1	-0.320512000	-14.887934000	-0.752490000
1	-2.007647000	-14.427066000	-0.965447000
7	-2.369179000	-2.602425000	3.619180000
6	-2.071425000	-3.336188000	2.339887000
6	-3.270084000	-3.907312000	1.616692000
6	-2.880439000	-4.369238000	0.217947000
6	-1.786647000	-5.422694000	0.188379000
6	-1.555481000	-5.998314000	-1.196520000
6	-0.414257000	-6.999172000	-1.230821000
6	-0.157667000	-7.612731000	-2.597902000
6	-1.254306000	-8.543260000	-3.086353000

6	-1.065253000	-2.237043000	4.237569000
6	-3.127679000	-3.460518000	4.563348000
6	-3.129630000	-1.352921000	3.353541000
6	-0.923155000	-9.179875000	-4.425600000
6	-2.047231000	-10.014362000	-5.020078000
6	-2.390207000	-11.262308000	-4.225750000
6	-3.493893000	-12.081921000	-4.871123000
6	-3.818393000	-13.380913000	-4.149573000
6	-4.376514000	-13.200538000	-2.748542000
6	-4.840260000	-14.503636000	-2.122903000
6	-5.347981000	-14.329684000	-0.704207000
1	-1.383120000	-4.128794000	2.620574000
1	-1.541819000	-2.623068000	1.711333000
1	-3.679069000	-4.750592000	2.174162000
1	-4.060012000	-3.162873000	1.527092000
1	-2.566570000	-3.502672000	-0.369201000
1	-3.774773000	-4.764910000	-0.267835000
1	-0.848301000	-4.997567000	0.552606000
1	-2.045475000	-6.232606000	0.877650000
1	-2.479175000	-6.464762000	-1.546854000
1	-1.336556000	-5.185230000	-1.895875000
1	0.495828000	-6.498920000	-0.888868000
1	-0.608898000	-7.797812000	-0.507410000
1	0.782540000	-8.169732000	-2.564680000
1	-0.013798000	-6.812085000	-3.329655000
1	-2.196390000	-7.996671000	-3.180027000
1	-1.421508000	-9.317888000	-2.333269000
1	-1.261032000	-1.680797000	5.148690000
1	-0.505231000	-1.632017000	3.531764000
1	-0.522838000	-3.150089000	4.462999000
1	-4.110328000	-3.661887000	4.152824000
1	-3.223118000	-2.929125000	5.504994000
1	-2.579087000	-4.386538000	4.706824000
1	-3.245796000	-0.822601000	4.293366000
1	-2.565313000	-0.755323000	2.644856000
1	-4.105124000	-1.602014000	2.953474000
1	-0.661212000	-8.387540000	-5.132373000
1	-0.027411000	-9.800265000	-4.318833000
1	-2.942531000	-9.391885000	-5.117680000
1	-1.771347000	-10.310294000	-6.035988000
1	-2.683929000	-10.984470000	-3.212487000
1	-1.493277000	-11.881924000	-4.125924000
1	-3.205137000	-12.311704000	-5.900511000
1	-4.400835000	-11.472611000	-4.939080000
1	-4.543588000	-13.942125000	-4.745355000
1	-2.918751000	-14.002729000	-4.100702000
1	-5.216806000	-12.499386000	-2.781640000
1	-3.623846000	-12.743996000	-2.100919000
1	-4.015585000	-15.221600000	-2.130831000
1	-5.627228000	-14.939363000	-2.744313000
1	-6.186015000	-13.631650000	-0.672180000

1	-4.564418000	-13.935297000	-0.055213000
1	-5.685978000	-15.275738000	-0.281179000
16	0.503829000	-0.763086000	0.494995000
8	1.356077000	-1.303598000	1.760761000
8	1.336695000	0.247242000	-0.159771000
8	0.247033000	-1.931221000	-0.349194000
8	-0.688641000	-0.231131000	1.150797000
6	2.118184000	-5.417043000	2.971505000
6	1.153787000	-6.540922000	2.640939000
6	1.931845000	-4.213404000	2.068027000
6	1.351136000	-7.784942000	3.487124000
6	2.860879000	-3.059249000	2.389866000
6	0.423923000	-8.933926000	3.118159000
6	2.650603000	-1.866502000	1.492182000
6	-1.042661000	-8.663968000	3.406055000
1	1.983718000	-5.115418000	4.014983000
1	3.147327000	-5.779893000	2.887281000
1	0.131779000	-6.171404000	2.752309000
1	1.263442000	-6.808637000	1.585680000
1	0.899027000	-3.868404000	2.134242000
1	2.084817000	-4.517914000	1.028463000
1	1.213739000	-7.533954000	4.543828000
1	2.387244000	-8.118609000	3.384143000
1	3.901731000	-3.372793000	2.279548000
1	2.736018000	-2.750870000	3.430871000
1	0.551032000	-9.167504000	2.057499000
1	0.733676000	-9.827453000	3.665016000
1	3.395134000	-1.092018000	1.673531000
1	2.706143000	-2.164957000	0.446198000
1	-1.426920000	-7.833199000	2.814400000
1	-1.192866000	-8.418176000	4.458948000
1	-1.653222000	-9.538074000	3.178656000
**1-CTA$^+$/ $(C_{10}\text{-}OSO_3)^-$ 1:1 ΔG=0.0**			
7	4.502555000	-0.204200000	-0.068728000
6	4.032806000	1.005349000	0.690907000
6	4.419129000	2.344887000	0.109377000
6	3.687030000	3.440894000	0.865753000
6	4.077154000	4.838768000	0.425906000
6	3.261982000	5.906801000	1.129922000
6	3.648781000	7.326320000	0.761329000
6	2.785615000	8.361127000	1.458575000
6	3.136425000	9.795017000	1.109919000
6	5.981115000	-0.213294000	-0.198717000
6	3.878193000	-0.257260000	-1.417085000
6	4.074344000	-1.407812000	0.695262000
6	2.242131000	10.802742000	1.807756000
6	2.554316000	12.245271000	1.455768000
6	1.632832000	13.229309000	2.155266000
6	1.843432000	14.683513000	1.763971000
6	3.189699000	15.256314000	2.171209000
6	3.330971000	16.730229000	1.838621000

6	4.674275000	17.312534000	2.237842000
6	4.804003000	18.785749000	1.900126000
1	4.437130000	0.900045000	1.695962000
1	2.951517000	0.910778000	0.746151000
1	5.495357000	2.501726000	0.185657000
1	4.152125000	2.407257000	-0.945517000
1	3.875928000	3.334984000	1.937697000
1	2.612669000	3.304451000	0.725870000
1	5.141706000	4.998892000	0.619703000
1	3.942493000	4.932017000	-0.655725000
1	2.203636000	5.756226000	0.899045000
1	3.358544000	5.778690000	2.212563000
1	4.700053000	7.496182000	1.013092000
1	3.567983000	7.455897000	-0.322332000
1	2.864161000	8.224468000	2.541609000
1	1.736510000	8.179044000	1.206502000
1	3.061697000	9.933325000	0.026846000
1	4.180840000	9.989966000	1.372085000
1	6.416181000	-0.141610000	0.793350000
1	6.290904000	0.626016000	-0.810308000
1	6.275614000	-1.144303000	-0.672615000
1	2.801396000	-0.194329000	-1.297604000
1	4.159935000	-1.195998000	-1.883390000
1	4.241392000	0.570876000	-2.013402000
1	4.548763000	-1.383306000	1.671074000
1	2.994128000	-1.380278000	0.796535000
1	4.386019000	-2.291741000	0.148325000
1	1.198795000	10.588839000	1.556722000
1	2.326152000	10.670164000	2.890966000
1	3.595614000	12.457704000	1.709910000
1	2.469166000	12.380689000	0.372715000
1	1.750324000	13.126279000	3.238777000
1	0.598192000	12.951660000	1.936515000
1	1.054146000	15.288672000	2.218651000
1	1.720280000	14.787329000	0.681282000
1	3.330013000	15.112967000	3.247398000
1	3.994895000	14.703467000	1.681330000
1	2.532875000	17.291535000	2.334528000
1	3.181673000	16.873942000	0.763896000
1	5.470458000	16.751306000	1.741387000
1	4.823210000	17.168127000	3.311312000
1	4.688498000	18.951807000	0.827981000
1	5.776411000	19.179949000	2.195373000
1	4.037754000	19.371910000	2.409719000
16	0.068941000	0.892893000	0.085849000
8	0.167445000	1.953658000	1.310239000
8	0.816247000	1.486269000	-1.024332000
8	0.705538000	-0.287337000	0.664565000
8	-1.357885000	0.731704000	-0.197188000
6	-0.309682000	6.446884000	3.241761000
6	-0.906295000	7.833262000	3.084209000

6	-0.675686000	5.510101000	2.105913000
6	-0.450467000	8.807696000	4.153910000
6	-0.121541000	4.108926000	2.289640000
6	-1.079487000	10.183034000	4.023959000
6	-0.371939000	3.258393000	1.071962000
6	-0.572216000	11.166748000	5.063561000
1	0.779489000	6.528491000	3.300311000
1	-0.637433000	6.015409000	4.192152000
1	-0.638407000	8.230644000	2.100446000
1	-1.998047000	7.760972000	3.097262000
1	-0.302062000	5.928817000	1.166944000
1	-1.763811000	5.461062000	2.008016000
1	0.638821000	8.902641000	4.110047000
1	-0.682078000	8.397620000	5.141761000
1	-0.579192000	3.637323000	3.161637000
1	0.952883000	4.156129000	2.476870000
1	-0.875594000	10.581513000	3.025113000
1	-2.165232000	10.084441000	4.098291000
1	-1.440058000	3.169593000	0.869084000
1	0.115874000	3.689631000	0.196633000
1	0.515230000	11.238813000	4.974055000
1	-0.770383000	10.769388000	6.064253000
6	-1.169083000	12.562035000	4.954055000
1	-0.635175000	13.226618000	5.637199000
1	-0.988358000	12.951169000	3.948565000
6	-2.654086000	12.625438000	5.264992000
1	-2.858271000	12.233194000	6.262889000
1	-3.242094000	12.045539000	4.554241000
1	-3.017477000	13.652709000	5.231048000
**2-CTA$^+$/ (C$_{10}$-OSO$_3$)$^-$ 1:1 ΔG=0.34**			
7	2.181780000	-1.996203000	-3.274079000
6	1.300309000	-0.811675000	-3.564678000
6	1.621247000	-0.043294000	-4.823837000
6	0.586759000	1.053057000	-5.019922000
6	0.895695000	1.949575000	-6.203900000
6	-0.158737000	3.023039000	-6.408392000
6	0.066237000	3.904834000	-7.626234000
6	1.319058000	4.760334000	-7.556797000
6	1.430573000	5.740778000	-8.709292000
6	3.614189000	-1.599958000	-3.238251000
6	1.984396000	-3.069853000	-4.280568000
6	1.799965000	-2.519501000	-1.934556000
6	2.651149000	6.637047000	-8.619560000
6	2.727121000	7.661932000	-9.735938000
6	3.859264000	8.656773000	-9.563739000
6	3.933781000	9.681238000	-10.680145000
6	5.044891000	10.696852000	-10.491266000
6	5.127545000	11.714658000	-11.613403000
6	6.237288000	12.732119000	-11.423037000
6	6.315604000	13.740772000	-12.553258000
1	1.388912000	-0.162394000	-2.696964000

1	0.285400000	-1.203939000	-3.599027000
1	2.609373000	0.409590000	-4.748197000
1	1.622183000	-0.696199000	-5.696897000
1	0.529721000	1.660779000	-4.113466000
1	-0.399520000	0.600170000	-5.151707000
1	1.875606000	2.408841000	-6.054336000
1	0.973759000	1.341948000	-7.110811000
1	-1.135940000	2.540520000	-6.495596000
1	-0.208590000	3.653694000	-5.515576000
1	0.100838000	3.281616000	-8.525394000
1	-0.798761000	4.563236000	-7.745476000
1	2.208062000	4.125298000	-7.538585000
1	1.319188000	5.315749000	-6.613703000
1	0.532428000	6.365688000	-8.735441000
1	1.447602000	5.191850000	-9.656038000
1	4.189864000	-2.450736000	-2.887912000
1	3.721483000	-0.756288000	-2.564587000
1	3.932716000	-1.326849000	-4.237653000
1	0.934621000	-3.346168000	-4.291017000
1	2.593842000	-3.921110000	-3.993813000
1	2.291205000	-2.710488000	-5.255623000
1	1.970371000	-1.737519000	-1.201834000
1	0.750617000	-2.796038000	-1.958380000
1	2.412710000	-3.388208000	-1.716324000
1	2.639737000	7.155362000	-7.656569000
1	3.557682000	6.024138000	-8.624329000
1	2.836030000	7.147386000	-10.695624000
1	1.779492000	8.206025000	-9.788132000
1	4.810632000	8.119502000	-9.500750000
1	3.736613000	9.174513000	-8.607305000
1	2.975507000	10.205191000	-10.753377000
1	4.072888000	9.165818000	-11.635422000
1	4.897964000	11.218960000	-9.540716000
1	6.002533000	10.173912000	-10.407388000
1	4.170032000	12.237904000	-11.698764000
1	5.276086000	11.193614000	-12.564333000
1	7.192669000	12.208192000	-11.332881000
1	6.085845000	13.255114000	-10.474932000
1	5.382047000	14.298059000	-12.644797000
1	6.498765000	13.244404000	-13.507455000
1	7.118584000	14.460173000	-12.392624000
16	2.995185000	1.922235000	-1.134168000
8	1.626554000	2.658922000	-1.598946000
8	3.628849000	2.817426000	-0.164747000
8	2.483984000	0.680741000	-0.558021000
8	3.785264000	1.734919000	-2.352269000
6	-0.872302000	6.409944000	-3.542077000
6	-0.839205000	7.829943000	-4.085152000
6	0.495817000	5.834408000	-3.219966000
6	-0.109395000	7.985067000	-5.407892000
6	0.410657000	4.500747000	-2.500235000

6	-0.174642000	9.399036000	-5.958246000
6	1.776284000	3.898935000	-2.298993000
6	0.601090000	9.558748000	-7.253853000
1	-1.482946000	6.394711000	-2.635388000
1	-1.375638000	5.756154000	-4.261036000
1	-1.866479000	8.184383000	-4.205968000
1	-0.376453000	8.486717000	-3.341818000
1	1.051342000	6.544972000	-2.600715000
1	1.072280000	5.711024000	-4.139468000
1	-0.534816000	7.290499000	-6.139889000
1	0.938898000	7.699137000	-5.291666000
1	-0.206621000	3.806439000	-3.073719000
1	-0.071863000	4.632223000	-1.529659000
1	-1.221496000	9.675726000	-6.106481000
1	0.223640000	10.095085000	-5.212846000
1	2.261733000	3.706656000	-3.256838000
1	2.410317000	4.565657000	-1.712746000
1	0.192707000	8.874122000	-8.003606000
1	1.631744000	9.239387000	-7.082337000
6	0.614954000	10.969626000	-7.820763000
1	1.008432000	11.656312000	-7.065838000
1	1.317931000	11.002906000	-8.656992000
6	-0.741717000	11.459152000	-8.294917000
1	-0.667832000	12.451644000	-8.739829000
1	-1.458092000	11.518531000	-7.475970000
1	-1.154675000	10.786095000	-9.048383000
**3-CTA$^+$/ $(C_{10}\text{-}OSO_3)^-$ 1:1 ΔG=0.52**			
7	-2.670857000	3.443364000	-2.865644000
6	-2.412619000	2.310927000	-3.823637000
6	-3.619448000	1.486601000	-4.199782000
6	-3.203668000	0.336775000	-5.102896000
6	-4.389989000	-0.493895000	-5.553877000
6	-3.999532000	-1.678109000	-6.417628000
6	-5.194364000	-2.489520000	-6.881280000
6	-4.823960000	-3.667109000	-7.762879000
6	-6.027345000	-4.473526000	-8.212978000
6	-3.608297000	4.434605000	-3.450819000
6	-1.367179000	4.109925000	-2.596394000
6	-3.214021000	2.940100000	-1.575703000
6	-5.676977000	-5.617496000	-9.145690000
6	-6.875357000	-6.470906000	-9.516194000
6	-6.547942000	-7.587722000	-10.489772000
6	-7.731577000	-8.497958000	-10.761394000
6	-7.458870000	-9.595503000	-11.780362000
6	-6.317541000	-10.541145000	-11.430824000
6	-6.480145000	-11.259826000	-10.102958000
6	-5.391473000	-12.289167000	-9.864019000
1	-1.655924000	1.693538000	-3.346587000
1	-1.972060000	2.768953000	-4.707616000
1	-4.100119000	1.078089000	-3.311310000
1	-4.357905000	2.099004000	-4.717734000

1	-2.492813000	-0.298873000	-4.570065000
1	-2.679880000	0.728958000	-5.978798000
1	-4.934974000	-0.851882000	-4.675229000
1	-5.086611000	0.143499000	-6.106339000
1	-3.442254000	-1.322696000	-7.289680000
1	-3.315490000	-2.323679000	-5.858301000
1	-5.745271000	-2.851324000	-6.007654000
1	-5.882816000	-1.836088000	-7.425914000
1	-4.132185000	-4.321041000	-7.222740000
1	-4.280784000	-3.304575000	-8.641048000
1	-6.741679000	-3.810509000	-8.710823000
1	-6.544199000	-4.870445000	-7.333640000
1	-4.577279000	3.971573000	-3.594292000
1	-3.205573000	4.773980000	-4.400165000
1	-3.697026000	5.267717000	-2.760672000
1	-0.703694000	3.385405000	-2.134790000
1	-1.540386000	4.946149000	-1.926455000
1	-0.956471000	4.461341000	-3.537579000
1	-2.538949000	2.179664000	-1.195642000
1	-3.268824000	3.775748000	-0.885191000
1	-4.205057000	2.532931000	-1.735739000
1	-5.219420000	-5.216104000	-10.055148000
1	-4.917110000	-6.248524000	-8.674588000
1	-7.300471000	-6.902800000	-8.604810000
1	-7.656659000	-5.835167000	-9.944158000
1	-5.717069000	-8.173972000	-10.091071000
1	-6.193607000	-7.158246000	-11.432246000
1	-8.570194000	-7.893543000	-11.118007000
1	-8.062303000	-8.941633000	-9.818630000
1	-8.371968000	-10.181944000	-11.915375000
1	-7.247338000	-9.134341000	-12.749091000
1	-5.368790000	-9.997759000	-11.427939000
1	-6.231763000	-11.286714000	-12.226263000
1	-7.459388000	-11.746344000	-10.073268000
1	-6.474820000	-10.534581000	-9.286133000
1	-4.404759000	-11.823898000	-9.886696000
1	-5.409315000	-13.062982000	-10.633181000
1	-5.506737000	-12.777002000	-8.895885000
16	0.132391000	0.075958000	-1.638368000
8	-1.222325000	-0.657035000	-2.141165000
8	-0.304352000	1.206258000	-0.814056000
8	0.886338000	-0.929039000	-0.886900000
8	0.744742000	0.466660000	-2.905290000
6	-3.970900000	-3.895254000	-3.162433000
6	-3.543442000	-5.104201000	-3.973457000
6	-2.798491000	-3.102947000	-2.615545000
6	-4.707521000	-5.925622000	-4.494551000
6	-3.230431000	-1.901690000	-1.795952000
6	-4.271648000	-7.183494000	-5.223584000
6	-2.092677000	-1.174392000	-1.127776000
6	-5.436113000	-7.990100000	-5.771772000

1	-4.593587000	-3.243497000	-3.782023000
1	-4.603078000	-4.223449000	-2.331806000
1	-2.925434000	-4.774641000	-4.814505000
1	-2.903132000	-5.741092000	-3.355154000
1	-2.165818000	-2.774089000	-3.442951000
1	-2.179312000	-3.756811000	-1.993849000
1	-5.318440000	-5.309230000	-5.160639000
1	-5.355286000	-6.203999000	-3.657586000
1	-3.907947000	-2.223437000	-1.001445000
1	-3.791180000	-1.202188000	-2.419010000
1	-3.592602000	-6.910343000	-6.035603000
1	-3.691108000	-7.809545000	-4.537973000
1	-2.461269000	-0.344895000	-0.523865000
1	-1.524481000	-1.851605000	-0.488329000
1	-5.926087000	-7.420196000	-6.567009000
1	-6.181950000	-8.113109000	-4.981529000
6	-5.053256000	-9.365919000	-6.296749000
1	-4.645373000	-9.959553000	-5.473650000
1	-5.959358000	-9.881524000	-6.624245000
6	-4.055427000	-9.340889000	-7.440345000
1	-3.867560000	-10.345929000	-7.817836000
1	-3.098426000	-8.921388000	-7.130722000
1	-4.428826000	-8.738015000	-8.269033000
**4-CTA$^+$/ (C_{10}-OSO_3)$^-$ 1:1 ΔG=0.60**			
7	0.374694000	-3.380914000	2.466717000
6	-0.733710000	-3.561560000	1.464660000
6	-2.123348000	-3.711339000	2.035090000
6	-3.115319000	-3.944086000	0.907115000
6	-4.541582000	-4.080502000	1.405123000
6	-5.536715000	-4.334231000	0.288510000
6	-6.970585000	-4.429175000	0.775396000
6	-7.961005000	-4.708957000	-0.342705000
6	-9.418924000	-4.553973000	0.061528000
6	1.630379000	-3.139168000	1.705003000
6	0.543917000	-4.599067000	3.299338000
6	0.123981000	-2.208277000	3.345215000
6	-9.878242000	-5.505211000	1.151961000
6	-11.333945000	-5.307881000	1.531938000
6	-11.808915000	-6.254958000	2.618304000
6	-13.273599000	-6.062752000	2.971202000
6	-13.749252000	-6.889524000	4.155582000
6	-13.715006000	-8.390813000	3.929578000
6	-14.280065000	-9.178705000	5.097496000
6	-14.241605000	-10.678482000	4.873620000
1	-0.461116000	-4.442256000	0.885728000
1	-0.685547000	-2.695968000	0.808085000
1	-2.174297000	-4.547799000	2.732467000
1	-2.413136000	-2.811695000	2.576646000
1	-2.834376000	-4.845459000	0.355706000
1	-3.057320000	-3.114875000	0.196712000
1	-4.595244000	-4.896465000	2.131693000

1	-4.824220000	-3.170701000	1.942776000
1	-5.461022000	-3.531589000	-0.451435000
1	-5.267062000	-5.257466000	-0.233617000
1	-7.037497000	-5.207807000	1.538861000
1	-7.242612000	-3.492664000	1.270762000
1	-7.793205000	-5.718419000	-0.730918000
1	-7.755763000	-4.026636000	-1.172184000
1	-10.050042000	-4.697523000	-0.819830000
1	-9.588223000	-3.524351000	0.391252000
1	2.447343000	-3.035727000	2.411967000
1	1.509880000	-2.227018000	1.129360000
1	1.805521000	-3.985749000	1.048771000
1	0.709565000	-5.447174000	2.642384000
1	-0.346189000	-4.750917000	3.898163000
1	1.402100000	-4.451760000	3.947327000
1	-0.025634000	-1.336442000	2.717135000
1	-0.749827000	-2.399179000	3.956998000
1	0.994154000	-2.072026000	3.979929000
1	-9.723430000	-6.537063000	0.820738000
1	-9.259969000	-5.374700000	2.043196000
1	-11.479409000	-4.275109000	1.865283000
1	-11.961090000	-5.432812000	0.643625000
1	-11.201149000	-6.108506000	3.516801000
1	-11.633926000	-7.283859000	2.294777000
1	-13.889193000	-6.296139000	2.096710000
1	-13.445093000	-5.005024000	3.188462000
1	-13.142035000	-6.644002000	5.032663000
1	-14.773326000	-6.596308000	4.402653000
1	-12.689010000	-8.718742000	3.744984000
1	-14.281118000	-8.632035000	3.023952000
1	-15.310380000	-8.861637000	5.279955000
1	-13.719693000	-8.929153000	6.002630000
1	-14.818993000	-10.956415000	3.990527000
1	-14.653532000	-11.220732000	5.724784000
1	-13.218157000	-11.024635000	4.721505000
16	-0.707018000	0.231316000	0.058413000
8	-1.867534000	-0.122565000	1.133525000
8	-0.845575000	-0.863034000	-0.898423000
8	-1.037135000	1.551511000	-0.479756000
8	0.551365000	0.209490000	0.808584000
6	-5.498853000	-0.485437000	3.471203000
6	-6.870495000	-0.674332000	2.851622000
6	-4.473148000	0.060127000	2.495966000
6	-7.884192000	-1.271784000	3.810125000
6	-3.112439000	0.267837000	3.132884000
6	-9.246163000	-1.489456000	3.174421000
6	-2.073733000	0.821122000	2.192536000
6	-10.326097000	-1.958690000	4.136759000
1	-5.578854000	0.187010000	4.330803000
1	-5.145058000	-1.443312000	3.865143000
1	-7.239016000	0.289414000	2.487416000

1	-6.781303000	-1.317744000	1.971382000
1	-4.826745000	1.010786000	2.085809000
1	-4.381090000	-0.626202000	1.651073000
1	-7.991476000	-0.614573000	4.679208000
1	-7.495168000	-2.219905000	4.189998000
1	-2.749112000	-0.669933000	3.558898000
1	-3.198161000	0.973079000	3.962828000
1	-9.571054000	-0.555104000	2.708481000
1	-9.150478000	-2.218163000	2.363900000
1	-2.403254000	1.766089000	1.758605000
1	-1.129006000	0.983418000	2.712421000
1	-11.276530000	-2.015113000	3.598165000
1	-10.465377000	-1.208046000	4.921487000
6	-10.053565000	-3.307335000	4.780374000
1	-9.824477000	-4.037047000	3.998925000
1	-9.163753000	-3.246030000	5.409950000
6	-11.222749000	-3.800953000	5.611565000
1	-11.015324000	-4.774087000	6.057035000
1	-12.120452000	-3.899171000	4.999440000
1	-11.448394000	-3.104444000	6.420938000
**5-CTA$^+$/ (C$_{10}$-OSO$_3$)$^-$ 1:1 ΔG=2.20**			
7	-2.699415000	-2.315710000	2.514320000
6	-1.236930000	-2.544291000	2.242061000
6	-0.395544000	-2.895203000	3.446571000
6	0.975264000	-3.368643000	2.991450000
6	1.937404000	-3.563683000	4.147793000
6	3.249841000	-4.201375000	3.732144000
6	4.237186000	-4.334236000	4.876516000
6	5.522877000	-5.039023000	4.486403000
6	6.500597000	-5.185125000	5.637616000
6	-2.891484000	-1.325138000	3.606210000
6	-3.376282000	-3.589277000	2.869802000
6	-3.313697000	-1.782348000	1.269076000
6	7.811908000	-5.835043000	5.235371000
6	8.785729000	-5.971239000	6.392828000
6	10.094747000	-6.661178000	6.039278000
6	10.977038000	-5.876148000	5.083871000
6	12.310156000	-6.555026000	4.821487000
6	13.205976000	-5.822353000	3.833909000
6	13.713239000	-4.476085000	4.322649000
6	14.704247000	-3.843543000	3.363607000
1	-0.871427000	-1.624528000	1.791678000
1	-1.200269000	-3.335650000	1.495886000
1	-0.284703000	-2.021356000	4.087959000
1	-0.860356000	-3.682484000	4.040216000
1	1.396297000	-2.646840000	2.286546000
1	0.864660000	-4.307972000	2.443053000
1	2.133262000	-2.595945000	4.618630000
1	1.463521000	-4.186707000	4.912106000
1	3.049231000	-5.191018000	3.310776000
1	3.702800000	-3.614040000	2.928103000

1	4.474388000	-3.338793000	5.265463000
1	3.763386000	-4.878193000	5.699602000
1	6.002918000	-4.489805000	3.670781000
1	5.284975000	-6.029661000	4.086880000
1	6.034340000	-5.770980000	6.435708000
1	6.704464000	-4.198484000	6.065020000
1	-3.951480000	-1.101831000	3.675114000
1	-2.320253000	-0.434438000	3.365636000
1	-2.543575000	-1.754784000	4.538586000
1	-2.949123000	-3.981647000	3.784957000
1	-3.238890000	-4.293080000	2.054914000
1	-4.432066000	-3.384011000	3.015443000
1	-2.851026000	-0.828380000	1.037579000
1	-3.140074000	-2.492098000	0.466280000
1	-4.378336000	-1.654639000	1.436170000
1	7.611953000	-6.825875000	4.815167000
1	8.265072000	-5.248082000	4.432687000
1	9.001783000	-4.978202000	6.800025000
1	8.296694000	-6.528972000	7.196303000
1	9.880798000	-7.645398000	5.610378000
1	10.655706000	-6.846562000	6.959486000
1	11.140092000	-4.877450000	5.496647000
1	10.461857000	-5.732058000	4.131198000
1	12.845563000	-6.678852000	5.768249000
1	12.120450000	-7.564381000	4.445985000
1	12.667943000	-5.682560000	2.890635000
1	14.067308000	-6.455019000	3.601790000
1	14.184197000	-4.606187000	5.301221000
1	12.873850000	-3.795043000	4.475220000
1	14.254622000	-3.688420000	2.381629000
1	15.579025000	-4.481935000	3.228856000
1	15.049991000	-2.875425000	3.726440000
16	0.394540000	1.124733000	3.758241000
8	1.764337000	0.292487000	3.519809000
8	-0.446255000	0.857890000	2.588269000
8	-0.101561000	0.536093000	4.998415000
8	0.787515000	2.530756000	3.874476000
6	5.937342000	-0.961764000	3.337903000
6	6.887318000	-0.850608000	4.517154000
6	4.697296000	-0.099834000	3.485702000
6	8.074258000	-1.798492000	4.445147000
6	3.751547000	-0.241657000	2.308166000
6	9.066740000	-1.498959000	3.334657000
6	2.542485000	0.653860000	2.372462000
6	10.183562000	-2.526621000	3.277853000
1	6.451746000	-0.687740000	2.413775000
1	5.636153000	-2.007224000	3.222472000
1	7.244820000	0.180457000	4.602363000
1	6.328093000	-1.055778000	5.434379000
1	4.990844000	0.949216000	3.589216000
1	4.176925000	-0.368524000	4.407988000

1	7.703466000	-2.819821000	4.323229000
1	8.605004000	-1.781471000	5.401109000
1	3.424574000	-1.279191000	2.215726000
1	4.278334000	0.003305000	1.382871000
1	8.556755000	-1.482794000	2.368105000
1	9.477489000	-0.496844000	3.483579000
1	2.837619000	1.700135000	2.464262000
1	1.927800000	0.537848000	1.479601000
1	9.735847000	-3.514136000	3.140737000
1	10.695190000	-2.559663000	4.244997000
6	11.208326000	-2.293063000	2.179102000
1	11.841644000	-3.178943000	2.095195000
1	10.691573000	-2.197009000	1.219930000
6	12.089592000	-1.078333000	2.407766000
1	12.621167000	-1.160521000	3.357593000
1	11.509129000	-0.156097000	2.431565000
1	12.834807000	-0.978348000	1.618279000
**1-CTA$^+$/ (C_{10}-OSO_3)$^-$ 2:1 ΔG=0.0**			
7	2.104027000	1.973618000	3.313584000
6	2.650809000	2.299217000	1.949789000
6	4.005811000	1.716402000	1.627421000
6	4.397248000	2.072897000	0.202399000
6	5.794155000	1.592218000	-0.144180000
6	6.165630000	1.854993000	-1.592740000
6	7.620481000	1.563838000	-1.928032000
6	8.033677000	0.115200000	-1.734436000
6	9.467254000	-0.147242000	-2.162799000
6	3.010813000	2.461374000	4.383223000
6	1.888106000	0.510837000	3.469797000
6	0.789569000	2.659126000	3.446374000
6	9.907823000	-1.596949000	-2.027772000
6	9.975634000	-2.106719000	-0.598900000
6	10.517771000	-3.521144000	-0.502586000
6	10.542841000	-4.062047000	0.914822000
6	11.123785000	-5.462400000	1.000572000
6	11.114209000	-6.068554000	2.395691000
6	9.726258000	-6.385844000	2.925834000
6	9.755661000	-7.066131000	4.281308000
1	2.685894000	3.386132000	1.900564000
1	1.903158000	1.950613000	1.242171000
1	4.762575000	2.102499000	2.310577000
1	3.998605000	0.631065000	1.727064000
1	4.341888000	3.156323000	0.066195000
1	3.674456000	1.635368000	-0.491114000
1	6.516701000	2.087165000	0.512112000
1	5.865748000	0.523844000	0.071040000
1	5.517352000	1.259409000	-2.241992000
1	5.953771000	2.901822000	-1.827204000
1	7.806246000	1.848522000	-2.967467000
1	8.264816000	2.204589000	-1.317579000
1	7.363260000	-0.532290000	-2.308161000

1	7.906549000	-0.164511000	-0.687721000
1	10.140272000	0.490026000	-1.580055000
1	9.583568000	0.162328000	-3.205251000
1	3.941303000	1.907402000	4.345239000
1	2.524995000	2.299744000	5.340048000
1	3.192022000	3.520365000	4.227571000
1	1.410111000	0.341621000	4.429655000
1	2.844060000	0.001943000	3.442764000
1	1.246265000	0.171593000	2.663024000
1	0.128860000	2.283755000	2.671337000
1	0.385616000	2.438101000	4.429141000
1	0.943961000	3.727629000	3.335046000
1	9.229749000	-2.234520000	-2.602696000
1	10.894028000	-1.710345000	-2.486582000
1	10.606385000	-1.436859000	-0.005595000
1	8.982959000	-2.075757000	-0.143338000
1	11.528175000	-3.550448000	-0.922441000
1	9.909972000	-4.185171000	-1.126352000
1	9.525063000	-4.053381000	1.311219000
1	11.126219000	-3.390148000	1.552397000
1	10.571018000	-6.119345000	0.323122000
1	12.151637000	-5.438010000	0.628156000
1	11.623836000	-5.393649000	3.091108000
1	11.700655000	-6.991682000	2.384332000
1	9.212250000	-7.027881000	2.205820000
1	9.133897000	-5.471300000	2.994689000
1	10.240142000	-6.433407000	5.026904000
1	10.309161000	-8.005470000	4.235613000
1	8.749471000	-7.289094000	4.637372000
7	-0.775318000	0.690390000	-4.885922000
6	0.575232000	0.186280000	-4.451701000
6	1.728929000	1.138406000	-4.666735000
6	3.018184000	0.568225000	-4.087842000
6	3.477459000	-0.730783000	-4.728538000
6	4.843502000	-1.178124000	-4.241971000
6	5.264638000	-2.522540000	-4.806747000
6	6.562342000	-3.043382000	-4.218177000
6	7.003122000	-4.368936000	-4.811297000
6	-0.787696000	1.005294000	-6.337020000
6	-1.176538000	1.892646000	-4.108688000
6	-1.761634000	-0.392360000	-4.619846000
6	8.222664000	-4.957071000	-4.125727000
6	8.703798000	-6.247640000	-4.766927000
6	9.859246000	-6.914859000	-4.034626000
6	9.459659000	-7.557224000	-2.717774000
6	10.617044000	-8.208455000	-1.983889000
6	10.189189000	-8.897248000	-0.701057000
6	11.345460000	-9.455308000	0.106791000
6	10.894756000	-10.122523000	1.392506000
1	0.724993000	-0.736527000	-5.006197000
1	0.469365000	-0.059340000	-3.397099000

1	1.872984000	1.331801000	-5.730098000
1	1.527388000	2.093995000	-4.184507000
1	2.898641000	0.421137000	-3.010729000
1	3.799310000	1.321382000	-4.208725000
1	2.758441000	-1.526486000	-4.518170000
1	3.496245000	-0.612689000	-5.816487000
1	5.590749000	-0.421523000	-4.499274000
1	4.831818000	-1.235623000	-3.150950000
1	4.471713000	-3.253423000	-4.619549000
1	5.358747000	-2.447299000	-5.894545000
1	6.442066000	-3.155826000	-3.136328000
1	7.353377000	-2.301102000	-4.360568000
1	7.209775000	-4.237935000	-5.878049000
1	6.178151000	-5.085429000	-4.746924000
1	-0.132059000	1.846797000	-6.527204000
1	-1.804224000	1.258586000	-6.620877000
1	-0.450856000	0.130178000	-6.884081000
1	-1.116836000	1.654401000	-3.051868000
1	-2.194145000	2.148895000	-4.386505000
1	-0.514038000	2.714625000	-4.352314000
1	-1.742762000	-0.616276000	-3.558540000
1	-2.744552000	-0.043481000	-4.919602000
1	-1.480584000	-1.265923000	-5.199524000
1	9.037652000	-4.227063000	-4.141304000
1	7.985206000	-5.125348000	-3.072165000
1	7.869119000	-6.952812000	-4.834404000
1	9.003393000	-6.033361000	-5.796182000
1	10.649377000	-6.178193000	-3.856590000
1	10.297774000	-7.681802000	-4.678522000
1	8.685193000	-8.306818000	-2.909740000
1	9.005278000	-6.811361000	-2.062006000
1	11.107545000	-8.933716000	-2.640473000
1	11.369295000	-7.447686000	-1.752884000
1	9.489762000	-9.704832000	-0.939420000
1	9.631794000	-8.188993000	-0.081211000
1	12.041463000	-8.644779000	0.340075000
1	11.903234000	-10.170657000	-0.503816000
1	10.356191000	-9.417573000	2.028072000
1	11.740535000	-10.506838000	1.962952000
1	10.224946000	-10.958909000	1.185918000
16	-0.030687000	0.473086000	-0.380084000
8	1.378125000	-0.311507000	-0.253913000
8	-0.580938000	0.533799000	0.975974000
8	0.396316000	1.765563000	-0.907608000
8	-0.855718000	-0.311371000	-1.300711000
6	5.128099000	-2.454361000	-0.276452000
6	5.260032000	-3.966397000	-0.233615000
6	3.738818000	-1.953315000	-0.637503000
6	6.682191000	-4.415191000	0.054662000
6	2.709215000	-2.162047000	0.458639000
6	6.881274000	-5.922471000	0.060742000

6	1.323574000	-1.704089000	0.082896000
6	6.203216000	-6.641590000	1.213873000
1	5.421744000	-2.034997000	0.691279000
1	5.844400000	-2.062672000	-1.002523000
1	4.581182000	-4.377314000	0.515699000
1	4.942542000	-4.379824000	-1.196852000
1	3.398703000	-2.447926000	-1.552910000
1	3.794112000	-0.888338000	-0.868107000
1	7.001602000	-4.005546000	1.018321000
1	7.343764000	-3.973223000	-0.695761000
1	3.025192000	-1.641898000	1.365531000
1	2.630331000	-3.219894000	0.714981000
1	6.523984000	-6.337775000	-0.887257000
1	7.952671000	-6.136969000	0.100193000
1	0.949064000	-2.253064000	-0.782178000
1	0.633362000	-1.844735000	0.915160000
1	5.127398000	-6.449519000	1.198641000
1	6.574107000	-6.230111000	2.158273000
6	6.432514000	-8.142199000	1.198439000
1	6.037091000	-8.556476000	0.266915000
1	7.506558000	-8.343827000	1.188239000
6	5.797710000	-8.848646000	2.381185000
1	5.972011000	-9.924115000	2.345518000
1	4.718866000	-8.686198000	2.402204000
1	6.206219000	-8.475832000	3.322042000

2-CTA$^+$/ (C_{10}-OSO_3)$^-$ 2:1 ΔG=1.72

7	-1.424580000	-3.569097000	2.442632000
6	-1.260437000	-4.129109000	1.054512000
6	-0.541669000	-5.457015000	0.975254000
6	-0.317237000	-5.876194000	-0.471894000
6	-1.589658000	-6.161785000	-1.249157000
6	-1.322332000	-6.698790000	-2.643248000
6	-2.595217000	-6.950736000	-3.427715000
6	-2.355666000	-7.453482000	-4.838484000
6	-3.637808000	-7.821158000	-5.561639000
6	-2.157253000	-4.518847000	3.318366000
6	-2.215957000	-2.312802000	2.334482000
6	-0.105356000	-3.242481000	3.045642000
6	-3.420092000	-8.237056000	-7.004557000
6	-4.693715000	-8.720692000	-7.674920000
6	-4.526042000	-9.138775000	-9.127122000
6	-4.197386000	-7.996626000	-10.073119000
6	-4.103874000	-8.435524000	-11.522830000
6	-3.766576000	-7.302396000	-12.473777000
6	-3.671260000	-7.740560000	-13.923593000
6	-3.330329000	-6.600969000	-14.865020000
1	-0.726092000	-3.366220000	0.491048000
1	-2.268358000	-4.207081000	0.654481000
1	0.426450000	-5.394145000	1.470002000
1	-1.117152000	-6.231990000	1.482040000

1	0.302844000	-6.774892000	-0.466001000
1	0.263713000	-5.106706000	-0.986780000
1	-2.197660000	-6.882751000	-0.693644000
1	-2.194724000	-5.255822000	-1.334401000
1	-0.692559000	-5.991242000	-3.190101000
1	-0.747794000	-7.627113000	-2.570752000
1	-3.215440000	-7.671556000	-2.885450000
1	-3.175312000	-6.024513000	-3.474353000
1	-1.697878000	-8.327608000	-4.808520000
1	-1.819930000	-6.687652000	-5.408303000
1	-4.329373000	-6.972990000	-5.532489000
1	-4.133040000	-8.632962000	-5.020011000
1	-3.089432000	-4.788769000	2.831439000
1	-2.356224000	-4.023788000	4.263432000
1	-1.547783000	-5.398827000	3.485395000
1	-1.690701000	-1.624071000	1.681300000
1	-2.321129000	-1.887163000	3.327293000
1	-3.191130000	-2.555745000	1.925248000
1	0.418476000	-2.571038000	2.372589000
1	-0.281842000	-2.760513000	4.001909000
1	0.457741000	-4.155516000	3.196744000
1	-2.999188000	-7.392267000	-7.555697000
1	-2.670011000	-9.033512000	-7.044918000
1	-5.086832000	-9.565358000	-7.103615000
1	-5.453005000	-7.935663000	-7.613875000
1	-5.449429000	-9.616924000	-9.466101000
1	-3.744959000	-9.902564000	-9.197101000
1	-3.253191000	-7.529640000	-9.782844000
1	-4.964407000	-7.221151000	-9.978497000
1	-3.346248000	-9.220030000	-11.613652000
1	-5.051968000	-8.890954000	-11.825010000
1	-2.817944000	-6.847507000	-12.172072000
1	-4.523266000	-6.516773000	-12.383847000
1	-4.619711000	-8.193320000	-14.224963000
1	-2.916583000	-8.526730000	-14.011608000
1	-3.267550000	-6.941363000	-15.898626000
1	-2.371071000	-6.151897000	-14.603154000
1	-4.086592000	-5.815983000	-14.817895000
7	-2.542443000	2.359070000	-3.092230000
6	-3.274020000	1.138202000	-2.599598000
6	-4.016580000	0.360921000	-3.663484000
6	-4.416821000	-1.016330000	-3.153656000
6	-5.377710000	-1.002980000	-1.976469000
6	-5.899355000	-2.387201000	-1.620124000
6	-4.822731000	-3.337318000	-1.120935000
6	-5.350330000	-4.681970000	-0.645577000
6	-5.964890000	-5.527303000	-1.748439000
6	-2.060549000	3.111182000	-1.901744000
6	-3.436678000	3.236825000	-3.888033000
6	-1.362367000	1.971151000	-3.909806000
6	-6.328192000	-6.928687000	-1.288476000

6	-6.785416000	-7.853620000	-2.406636000
6	-8.043992000	-7.390139000	-3.121561000
6	-8.616024000	-8.410895000	-4.094242000
6	-7.669084000	-8.784348000	-5.224904000
6	-8.332652000	-9.549154000	-6.358831000
6	-8.850741000	-10.922727000	-5.969555000
6	-9.434959000	-11.680417000	-7.147046000
1	-3.957631000	1.496041000	-1.833418000
1	-2.517903000	0.521534000	-2.119147000
1	-4.907443000	0.906254000	-3.976706000
1	-3.391996000	0.229847000	-4.546356000
1	-3.513576000	-1.569329000	-2.891831000
1	-4.881204000	-1.559862000	-3.979209000
1	-4.892687000	-0.569115000	-1.097593000
1	-6.222513000	-0.351690000	-2.216620000
1	-6.667010000	-2.290696000	-0.847312000
1	-6.396666000	-2.812047000	-2.495683000
1	-4.083269000	-3.511682000	-1.907489000
1	-4.286328000	-2.851692000	-0.300825000
1	-6.087571000	-4.525886000	0.148458000
1	-4.527868000	-5.242029000	-0.191759000
1	-5.256734000	-5.596233000	-2.580097000
1	-6.851213000	-5.028827000	-2.143616000
1	-1.450507000	2.446771000	-1.299001000
1	-2.922844000	3.449953000	-1.336294000
1	-1.478159000	3.960481000	-2.244230000
1	-3.713178000	2.728438000	-4.804396000
1	-2.897758000	4.150046000	-4.119182000
1	-4.319705000	3.460149000	-3.297049000
1	-0.704640000	1.364738000	-3.294607000
1	-1.691630000	1.416179000	-4.780220000
1	-0.854619000	2.878323000	-4.222275000
1	-5.457482000	-7.373905000	-0.798451000
1	-7.110061000	-6.867673000	-0.524687000
1	-5.971491000	-7.962834000	-3.128297000
1	-6.962309000	-8.850613000	-1.992325000
1	-7.839472000	-6.463559000	-3.663782000
1	-8.805099000	-7.146216000	-2.374362000
1	-9.535941000	-8.006179000	-4.526771000
1	-8.908065000	-9.307416000	-3.541080000
1	-7.225701000	-7.870956000	-5.628705000
1	-6.836920000	-9.375893000	-4.834380000
1	-7.616690000	-9.665031000	-7.177497000
1	-9.159120000	-8.953047000	-6.759614000
1	-9.609334000	-10.826602000	-5.190083000
1	-8.032524000	-11.500840000	-5.530607000
1	-9.801601000	-12.662807000	-6.849153000
1	-8.686965000	-11.825795000	-7.928122000
1	-10.269815000	-11.133083000	-7.587601000
16	-0.026717000	-0.105730000	-0.309769000
8	-0.570781000	-1.203244000	-1.370637000

8	-1.274036000	0.427394000	0.230458000
8	0.777618000	-0.847583000	0.663269000
8	0.749296000	0.859889000	-1.089602000
6	-1.766243000	-3.211683000	-5.005850000
6	-2.315036000	-2.595523000	-6.279921000
6	-0.885202000	-2.264725000	-4.212033000
6	-3.233369000	-3.512619000	-7.073920000
6	-0.294404000	-2.931260000	-2.983905000
6	-4.550358000	-3.823344000	-6.382850000
6	0.417793000	-1.995163000	-2.043426000
6	-5.519841000	-4.592202000	-7.261188000
1	-2.587956000	-3.541821000	-4.365153000
1	-1.198176000	-4.112411000	-5.259604000
1	-2.857475000	-1.677403000	-6.033906000
1	-1.476118000	-2.293205000	-6.912486000
1	-1.469212000	-1.391207000	-3.914082000
1	-0.074759000	-1.891547000	-4.844788000
1	-2.711331000	-4.448541000	-7.298674000
1	-3.448649000	-3.045298000	-8.038690000
1	0.421681000	-3.695915000	-3.291521000
1	-1.078095000	-3.447847000	-2.425524000
1	-4.369627000	-4.393695000	-5.467694000
1	-5.019690000	-2.885068000	-6.071315000
1	0.983185000	-2.552126000	-1.296340000
1	1.099463000	-1.333391000	-2.580679000
1	-5.048590000	-5.520009000	-7.599771000
1	-5.729759000	-4.009506000	-8.163648000
6	-6.825878000	-4.918727000	-6.559880000
1	-6.615460000	-5.537495000	-5.683078000
1	-7.268928000	-3.994247000	-6.179068000
6	-7.825948000	-5.625783000	-7.454760000
1	-7.418879000	-6.562623000	-7.837817000
1	-8.089519000	-5.004882000	-8.312523000
1	-8.745226000	-5.860934000	-6.917564000
**3-CTA$^+$/ $(C_{10}$-$OSO_3)^-$ 2:1 ΔG=2.95**			
7	1.521672000	-4.838146000	0.451240000
6	0.255066000	-4.759424000	1.260376000
6	-1.026628000	-4.960572000	0.487665000
6	-2.215441000	-4.880287000	1.431946000
6	-3.539340000	-4.953963000	0.692232000
6	-4.754152000	-4.705821000	1.572254000
6	-4.969260000	-5.751492000	2.654778000
6	-6.293332000	-5.606094000	3.390817000
6	-6.405058000	-4.348573000	4.235224000
6	1.719727000	-6.203701000	-0.096721000
6	2.655864000	-4.514562000	1.358534000
6	1.509906000	-3.848825000	-0.658571000
6	-7.740767000	-4.223658000	4.944838000
6	-7.848801000	-2.977202000	5.804421000
6	-9.211112000	-2.824203000	6.459023000
6	-9.320602000	-1.648707000	7.418099000

6	-9.153526000	-0.287486000	6.766230000
6	-9.332615000	0.863203000	7.739431000
6	-9.058743000	2.219891000	7.116574000
6	-9.192550000	3.366527000	8.100076000
1	0.267928000	-3.775050000	1.723521000
1	0.358770000	-5.510025000	2.042069000
1	-1.133648000	-4.190439000	-0.276454000
1	-1.035083000	-5.926309000	-0.018196000
1	-2.169450000	-3.942331000	1.990255000
1	-2.146361000	-5.682385000	2.169182000
1	-3.530933000	-4.211832000	-0.110498000
1	-3.630624000	-5.930506000	0.207179000
1	-4.663900000	-3.714165000	2.021842000
1	-5.644696000	-4.673251000	0.938129000
1	-4.926208000	-6.743054000	2.195025000
1	-4.151346000	-5.714842000	3.379466000
1	-6.440539000	-6.476278000	4.036284000
1	-7.110780000	-5.625043000	2.662987000
1	-6.254304000	-3.464821000	3.611184000
1	-5.597741000	-4.340914000	4.974175000
1	0.926846000	-6.427318000	-0.800498000
1	1.707909000	-6.911766000	0.725970000
1	2.679409000	-6.231420000	-0.603003000
1	2.517035000	-3.504636000	1.730025000
1	3.579700000	-4.589677000	0.793703000
1	2.656334000	-5.225887000	2.178366000
1	2.488463000	-3.860112000	-1.128250000
1	0.753989000	-4.132338000	-1.381308000
1	1.299288000	-2.867389000	-0.245397000
1	-8.543084000	-4.218563000	4.200342000
1	-7.908409000	-5.108704000	5.566484000
1	-7.076185000	-3.003999000	6.579319000
1	-7.630442000	-2.101834000	5.187458000
1	-9.444571000	-3.744621000	7.001350000
1	-9.974917000	-2.728514000	5.680811000
1	-8.571717000	-1.759574000	8.208967000
1	-10.294351000	-1.685571000	7.914308000
1	-8.162248000	-0.214212000	6.313881000
1	-9.871659000	-0.186826000	5.945904000
1	-8.662576000	0.718211000	8.592313000
1	-10.349050000	0.845411000	8.144988000
1	-9.739252000	2.374791000	6.274799000
1	-8.050412000	2.218112000	6.695523000
1	-8.994358000	4.326452000	7.622596000
1	-8.488590000	3.254220000	8.926451000
1	-10.197396000	3.406024000	8.523815000
7	-0.396587000	3.238146000	4.274429000
6	-1.154377000	4.150949000	5.199676000
6	-2.611136000	4.353042000	4.846415000
6	-3.379952000	4.989131000	5.996588000
6	-3.457621000	4.106132000	7.234142000

6	-4.646205000	4.430301000	8.121983000
6	-4.669219000	3.650251000	9.432199000
6	-4.466135000	2.147805000	9.285268000
6	-5.498638000	1.463723000	8.405110000
6	1.040192000	3.277507000	4.658159000
6	-0.525494000	3.674873000	2.859421000
6	-0.875374000	1.836519000	4.407797000
6	-5.218552000	-0.009068000	8.144561000
6	-3.964043000	-0.258790000	7.323743000
6	-3.770724000	-1.712730000	6.934643000
6	-2.460537000	-1.955915000	6.206969000
6	-2.321754000	-3.371160000	5.674646000
6	-1.015901000	-3.644974000	4.944271000
6	0.221930000	-3.600070000	5.823834000
6	1.483952000	-3.971370000	5.067382000
1	-0.625128000	5.101786000	5.187591000
1	-1.041267000	3.711167000	6.187241000
1	-2.693513000	4.983357000	3.961713000
1	-3.086619000	3.400903000	4.610051000
1	-4.390812000	5.192218000	5.640437000
1	-2.945503000	5.959391000	6.250045000
1	-3.523415000	3.063658000	6.912478000
1	-2.537533000	4.186345000	7.818635000
1	-4.648430000	5.499482000	8.350107000
1	-5.563807000	4.243105000	7.557474000
1	-3.886813000	4.040839000	10.088410000
1	-5.618139000	3.842438000	9.940643000
1	-4.487606000	1.689491000	10.277889000
1	-3.464388000	1.964358000	8.894849000
1	-5.563865000	1.976775000	7.442536000
1	-6.481426000	1.570879000	8.870287000
1	1.403912000	4.292650000	4.537105000
1	1.584952000	2.598642000	4.010125000
1	1.128737000	2.966287000	5.694294000
1	-0.204011000	4.709523000	2.788038000
1	-1.560121000	3.578936000	2.550640000
1	0.104222000	3.033415000	2.251565000
1	-0.792416000	1.540644000	5.449213000
1	-1.904577000	1.773998000	4.078139000
1	-0.253679000	1.208088000	3.779695000
1	-5.143026000	-0.544795000	9.096235000
1	-6.073183000	-0.442787000	7.617696000
1	-4.004037000	0.350849000	6.414208000
1	-3.082801000	0.077640000	7.874963000
1	-4.606599000	-2.033182000	6.305336000
1	-3.808416000	-2.339882000	7.830866000
1	-1.635405000	-1.724910000	6.884922000
1	-2.374220000	-1.252830000	5.372967000
1	-2.427751000	-4.082218000	6.500472000
1	-3.155406000	-3.569551000	4.994938000
1	-0.901371000	-2.928275000	4.124723000

1	-1.073645000	-4.630876000	4.474959000
1	0.085431000	-4.281699000	6.668323000
1	0.339662000	-2.602839000	6.252287000
1	2.363287000	-3.930666000	5.710560000
1	1.650019000	-3.292577000	4.229934000
1	1.413564000	-4.981856000	4.661226000
16	0.871235000	-0.181784000	1.224104000
8	-0.720469000	0.112433000	1.275699000
8	1.221128000	-0.284384000	-0.193460000
8	1.414379000	0.996337000	1.895545000
8	1.075286000	-1.431916000	1.958879000
6	-4.606973000	-0.727658000	3.083013000
6	-5.127862000	0.699660000	3.074446000
6	-3.180132000	-0.893831000	2.588970000
6	-6.496288000	0.803407000	3.727216000
6	-2.990013000	-0.613415000	1.109402000
6	-7.166365000	2.161387000	3.596041000
6	-1.576210000	-0.848590000	0.641338000
6	-6.415759000	3.293669000	4.278395000
1	-4.667731000	-1.111805000	4.104505000
1	-5.274415000	-1.355439000	2.484925000
1	-4.416179000	1.346445000	3.596002000
1	-5.188909000	1.073296000	2.048534000
1	-2.850721000	-1.915257000	2.792504000
1	-2.520469000	-0.244181000	3.165355000
1	-6.402953000	0.547481000	4.787799000
1	-7.151350000	0.044679000	3.289456000
1	-3.278595000	0.410153000	0.863987000
1	-3.636408000	-1.271782000	0.523320000
1	-7.301952000	2.389716000	2.535716000
1	-8.170448000	2.098931000	4.025407000
1	-1.246157000	-1.851323000	0.914314000
1	-1.491407000	-0.730257000	-0.438634000
1	-5.475078000	3.485775000	3.755833000
1	-6.142987000	2.975446000	5.288965000
6	-7.207576000	4.589609000	4.365806000
1	-8.143793000	4.402022000	4.899195000
1	-6.649729000	5.305305000	4.973677000
6	-7.509230000	5.218196000	3.016813000
1	-8.143963000	4.577122000	2.405443000
1	-6.588481000	5.400716000	2.459604000
1	-8.022257000	6.172887000	3.134146000
**4-CTA$^+$/ (C_{10}-OSO_3)$^-$ 2:1 ΔG=3.30**			
7	-0.500697000	-1.369878000	-4.321593000
6	0.878692000	-0.947320000	-3.894663000
6	1.520905000	0.145546000	-4.714687000
6	2.918024000	0.422853000	-4.184037000
6	3.665759000	1.454597000	-5.008625000
6	4.997652000	1.831316000	-4.383570000
6	5.910170000	2.650907000	-5.281467000
6	5.344858000	4.001159000	-5.694247000

6	6.362703000	4.903316000	-6.377784000
6	-1.457358000	-0.235702000	-4.235950000
6	-0.947520000	-2.439255000	-3.387216000
6	-0.493848000	-1.912600000	-5.703479000
6	6.838995000	4.392786000	-7.728202000
6	7.917148000	5.250286000	-8.374508000
6	7.464423000	6.635149000	-8.805026000
6	8.570596000	7.412728000	-9.498968000
6	8.173873000	8.806311000	-9.966219000
6	7.948582000	9.791389000	-8.831498000
6	7.519652000	11.180482000	-9.279191000
6	8.577825000	11.935085000	-10.064601000
1	1.483603000	-1.851561000	-3.927753000
1	0.779994000	-0.637766000	-2.857343000
1	1.589408000	-0.144350000	-5.763253000
1	0.934186000	1.063265000	-4.663459000
1	3.493022000	-0.506832000	-4.167900000
1	2.848613000	0.759998000	-3.146968000
1	3.829984000	1.059571000	-6.015785000
1	3.044348000	2.346145000	-5.125640000
1	4.810529000	2.380871000	-3.457117000
1	5.522419000	0.916100000	-4.095633000
1	6.856853000	2.816272000	-4.758539000
1	6.151845000	2.061382000	-6.169209000
1	4.962642000	4.509383000	-4.804371000
1	4.485983000	3.861746000	-6.356795000
1	7.227628000	5.029990000	-5.717721000
1	5.927455000	5.895631000	-6.500928000
1	-1.429724000	0.158015000	-3.225211000
1	-2.448604000	-0.611417000	-4.469676000
1	-1.174312000	0.525166000	-4.953820000
1	-0.254743000	-3.271581000	-3.460408000
1	-1.945451000	-2.753876000	-3.675634000
1	-0.951912000	-2.032566000	-2.381102000
1	-0.251999000	-1.119228000	-6.400635000
1	0.243389000	-2.707257000	-5.760685000
1	-1.483834000	-2.301719000	-5.919073000
1	7.229606000	3.379936000	-7.614572000
1	5.981292000	4.314972000	-8.404623000
1	8.758859000	5.347425000	-7.681042000
1	8.306698000	4.724585000	-9.250750000
1	7.103865000	7.193089000	-7.939672000
1	6.611047000	6.540250000	-9.484170000
1	8.913506000	6.832648000	-10.360080000
1	9.430780000	7.492890000	-8.826101000
1	8.954485000	9.186637000	-10.628028000
1	7.264986000	8.739073000	-10.573118000
1	8.867541000	9.873134000	-8.241898000
1	7.190510000	9.396174000	-8.154261000
1	7.251465000	11.763695000	-8.394792000
1	6.607545000	11.099466000	-9.877507000

1	8.244197000	12.945483000	-10.302127000
1	8.814983000	11.439009000	-11.005387000
1	9.502378000	12.016002000	-9.490201000
7	4.453832000	-0.159613000	2.089698000
6	4.570583000	0.631676000	0.814354000
6	4.668612000	2.129488000	0.978492000
6	4.858851000	2.781991000	-0.381052000
6	4.932640000	4.294442000	-0.290954000
6	5.083643000	4.970151000	-1.640856000
6	5.215321000	6.478512000	-1.538470000
6	5.240044000	7.174668000	-2.886633000
6	5.513211000	8.665356000	-2.779134000
6	5.682411000	-0.029498000	2.913696000
6	4.276911000	-1.590016000	1.716257000
6	3.273195000	0.273507000	2.882711000
6	5.279679000	9.435465000	-4.071660000
6	6.028875000	8.895162000	-5.282690000
6	7.542561000	8.849036000	-5.141971000
6	8.192794000	10.218465000	-5.061597000
6	9.709396000	10.148907000	-5.024893000
6	10.397477000	11.505389000	-4.996832000
6	10.269727000	12.294966000	-6.288975000
6	11.001405000	13.623016000	-6.238901000
1	3.693305000	0.373218000	0.225552000
1	5.453058000	0.242003000	0.310012000
1	3.760910000	2.524011000	1.435620000
1	5.505131000	2.400101000	1.623023000
1	4.034796000	2.494443000	-1.038524000
1	5.772352000	2.399686000	-0.844427000
1	4.030841000	4.671775000	0.200554000
1	5.771640000	4.576032000	0.352401000
1	5.959953000	4.565447000	-2.156433000
1	4.221817000	4.722668000	-2.267178000
1	4.385345000	6.875802000	-0.945755000
1	6.127840000	6.722779000	-0.985553000
1	4.281610000	7.015620000	-3.391018000
1	5.996850000	6.703768000	-3.519367000
1	6.539099000	8.817054000	-2.432219000
1	4.869730000	9.089973000	-2.003580000
1	6.534721000	-0.335734000	2.314952000
1	5.578656000	-0.676167000	3.779218000
1	5.794025000	0.999155000	3.235017000
1	4.209404000	-2.177229000	2.626571000
1	5.135592000	-1.902253000	1.130451000
1	3.364998000	-1.679045000	1.135780000
1	3.428911000	1.289083000	3.226976000
1	2.391732000	0.213264000	2.252790000
1	3.178032000	-0.392048000	3.734946000
1	4.210005000	9.419747000	-4.297226000
1	5.537922000	10.484768000	-3.912455000
1	5.658563000	7.893419000	-5.507219000

1	5.774697000	9.507872000	-6.152077000
1	7.957769000	8.317290000	-6.002133000
1	7.820842000	8.259435000	-4.264092000
1	7.841065000	10.750173000	-4.173166000
1	7.866854000	10.808832000	-5.921520000
1	10.012482000	9.574834000	-4.144993000
1	10.065053000	9.585089000	-5.893387000
1	11.460052000	11.362383000	-4.781542000
1	9.996828000	12.098744000	-4.168578000
1	9.216768000	12.472701000	-6.516231000
1	10.661061000	11.691683000	-7.112793000
1	10.902830000	14.168654000	-7.177509000
1	12.065846000	13.477177000	-6.048169000
1	10.607181000	14.256379000	-5.442636000
16	0.408739000	0.316230000	-0.061299000
8	1.157881000	1.401822000	-1.003046000
8	1.427789000	-0.722742000	0.062439000
8	0.106169000	1.007445000	1.192557000
8	-0.786100000	-0.101812000	-0.797279000
6	1.334101000	5.552984000	-3.626852000
6	0.510226000	6.604877000	-4.348045000
6	0.465855000	4.559265000	-2.877150000
6	1.319814000	7.653335000	-5.094154000
6	1.265155000	3.464102000	-2.196335000
6	2.152627000	7.109792000	-6.243570000
6	0.361888000	2.489310000	-1.487697000
6	2.713267000	8.207369000	-7.129562000
1	1.959613000	5.011223000	-4.341306000
1	2.018369000	6.041450000	-2.926903000
1	-0.162770000	6.107179000	-5.053411000
1	-0.131772000	7.106598000	-3.618772000
1	-0.241224000	4.104179000	-3.576898000
1	-0.136099000	5.091542000	-2.135042000
1	1.972986000	8.179831000	-4.391552000
1	0.630480000	8.404670000	-5.488975000
1	1.965598000	3.897989000	-1.480611000
1	1.854723000	2.927036000	-2.941705000
1	2.975723000	6.501740000	-5.857419000
1	1.534966000	6.439555000	-6.850400000
1	-0.145551000	2.967179000	-0.648220000
1	-0.390962000	2.093584000	-2.169586000
1	3.311920000	8.893510000	-6.523901000
1	1.885424000	8.799515000	-7.531971000
6	3.560958000	7.692281000	-8.277662000
1	4.395424000	7.111458000	-7.876552000
1	2.968542000	6.998793000	-8.880954000
6	4.093510000	8.806580000	-9.158456000
1	3.276162000	9.375361000	-9.605081000
1	4.711552000	8.418710000	-9.968142000
1	4.703221000	9.502559000	-8.580262000

5-CTA$^+$/ $(C_{10}$-$OSO_3)^-$ 2:1 ΔG=5.73

7	-2.590022000	-3.142264000	-2.032210000
6	-1.508828000	-2.474231000	-2.839585000
6	-1.861420000	-2.200920000	-4.284905000
6	-0.818491000	-1.299296000	-4.935426000
6	-0.972719000	0.170526000	-4.586015000
6	0.061092000	1.050095000	-5.266200000
6	-0.164976000	2.531010000	-5.015360000
6	0.751583000	3.451999000	-5.806056000
6	2.220865000	3.350442000	-5.436668000
6	-3.705751000	-2.193902000	-1.771294000
6	-1.999658000	-3.547780000	-0.727405000
6	-3.110877000	-4.351171000	-2.718872000
6	3.084559000	4.372974000	-6.152883000
6	4.524158000	4.374815000	-5.674073000
6	5.386881000	5.419431000	-6.362824000
6	6.741924000	5.640861000	-5.706866000
6	7.613463000	4.399383000	-5.631857000
6	8.987408000	4.679584000	-5.048743000
6	9.903998000	3.466271000	-4.992719000
6	9.436483000	2.377657000	-4.042583000
1	-0.646150000	-3.135923000	-2.789040000
1	-1.269611000	-1.561482000	-2.299022000
1	-1.912627000	-3.142487000	-4.829452000
1	-2.839553000	-1.726702000	-4.369967000
1	-0.897986000	-1.410371000	-6.018316000
1	0.184558000	-1.643841000	-4.667896000
1	-1.973530000	0.497838000	-4.882583000
1	-0.913382000	0.313216000	-3.504315000
1	1.058755000	0.758167000	-4.928808000
1	0.035046000	0.863613000	-6.344659000
1	-1.202226000	2.770750000	-5.264461000
1	-0.052320000	2.740602000	-3.947142000
1	0.427138000	4.484946000	-5.651977000
1	0.629871000	3.251353000	-6.875283000
1	2.597082000	2.346885000	-5.650188000
1	2.325061000	3.493698000	-4.357457000
1	-3.301902000	-1.327813000	-1.256473000
1	-4.440513000	-2.698497000	-1.151642000
1	-4.157496000	-1.904272000	-2.712730000
1	-1.228727000	-4.288817000	-0.914263000
1	-2.786164000	-3.970982000	-0.110883000
1	-1.568827000	-2.670889000	-0.256949000
1	-2.274500000	-4.999284000	-2.962262000
1	-3.793402000	-4.855542000	-2.042396000
1	-3.636053000	-4.054405000	-3.619251000
1	3.053472000	4.189361000	-7.231501000
1	2.658441000	5.369234000	-6.000697000
1	4.535049000	4.553424000	-4.595084000
1	4.953505000	3.380440000	-5.818771000
1	4.846686000	6.370331000	-6.375732000
1	5.529949000	5.136835000	-7.410443000

1	6.589161000	6.032153000	-4.695676000
1	7.279536000	6.420176000	-6.254451000
1	7.111041000	3.637859000	-5.033767000
1	7.727996000	3.976485000	-6.635694000
1	8.874139000	5.092909000	-4.040565000
1	9.469532000	5.458379000	-5.645061000
1	10.902374000	3.793110000	-4.693042000
1	10.009652000	3.051717000	-5.999317000
1	8.473283000	1.965157000	-4.342167000
1	9.329063000	2.766707000	-3.028791000
1	10.150489000	1.554432000	-4.009164000
7	2.256569000	-1.866855000	3.736012000
6	2.759575000	-0.844460000	2.753914000
6	3.641168000	-1.365340000	1.643908000
6	3.937148000	-0.229612000	0.678062000
6	4.789473000	-0.663662000	-0.501987000
6	4.880120000	0.387180000	-1.597786000
6	5.595953000	1.663447000	-1.192298000
6	5.511991000	2.736896000	-2.262049000
6	6.278481000	4.009838000	-1.942166000
6	3.382405000	-2.531479000	4.438853000
6	1.414368000	-2.886669000	3.057018000
6	1.416065000	-1.150706000	4.733932000
6	5.881634000	4.657896000	-0.623959000
6	6.320628000	6.107310000	-0.493674000
6	7.811920000	6.342936000	-0.659301000
6	8.194833000	7.786722000	-0.387108000
6	9.668249000	8.103464000	-0.599651000
6	10.181706000	7.912869000	-2.021169000
6	9.433798000	8.721195000	-3.067449000
6	10.091800000	8.661917000	-4.433181000
1	3.290281000	-0.102017000	3.347083000
1	1.867946000	-0.381943000	2.339106000
1	4.575653000	-1.763487000	2.040190000
1	3.144116000	-2.168144000	1.100032000
1	4.424870000	0.582986000	1.219650000
1	2.990475000	0.168858000	0.306652000
1	5.793051000	-0.926464000	-0.154660000
1	4.362242000	-1.576440000	-0.925213000
1	5.384982000	-0.041057000	-2.467664000
1	3.866591000	0.636747000	-1.927932000
1	5.165796000	2.048977000	-0.266832000
1	6.645313000	1.439632000	-0.973195000
1	4.458936000	2.987035000	-2.422856000
1	5.876211000	2.328534000	-3.209110000
1	6.118741000	4.725808000	-2.752944000
1	7.349827000	3.795370000	-1.937367000
1	3.961796000	-3.103450000	3.723625000
1	2.971210000	-3.193535000	5.194149000
1	3.999702000	-1.768276000	4.902587000
1	0.643949000	-2.373054000	2.490965000

1	0.973355000	-3.521894000	3.818685000
1	2.034305000	-3.482642000	2.397924000
1	1.039686000	-1.873878000	5.450105000
1	2.030730000	-0.409670000	5.234882000
1	0.595929000	-0.672622000	4.207751000
1	4.793727000	4.611699000	-0.514442000
1	6.293304000	4.081537000	0.208577000
1	5.791538000	6.707801000	-1.237950000
1	6.005623000	6.487047000	0.482691000
1	8.110987000	6.066720000	-1.672627000
1	8.369452000	5.684850000	0.015699000
1	7.932607000	8.030389000	0.646092000
1	7.581749000	8.443016000	-1.010613000
1	9.846857000	9.140693000	-0.302664000
1	10.265815000	7.486487000	0.077412000
1	10.149197000	6.855307000	-2.296837000
1	11.237881000	8.195444000	-2.045342000
1	9.366937000	9.762418000	-2.738529000
1	8.405845000	8.360471000	-3.145967000
1	9.524620000	9.224460000	-5.175101000
1	10.169777000	7.632517000	-4.785150000
1	11.100703000	9.076226000	-4.399386000
16	-0.487877000	0.006337000	0.602042000
8	0.395693000	1.332673000	0.314637000
8	0.314225000	-1.125685000	0.136324000
8	-0.648426000	0.063504000	2.052799000
8	-1.734095000	0.172913000	-0.148853000
6	1.540833000	5.390854000	-0.820505000
6	2.090823000	5.748430000	-2.190769000
6	0.743744000	4.095569000	-0.779868000
6	2.716220000	7.131587000	-2.242753000
6	1.553083000	2.856203000	-1.118834000
6	3.274290000	7.467931000	-3.615069000
6	0.754971000	1.579944000	-1.051284000
6	3.864829000	8.863538000	-3.745263000
1	2.365832000	5.332002000	-0.103943000
1	0.897196000	6.204858000	-0.476559000
1	2.838088000	5.011855000	-2.496935000
1	1.284449000	5.689443000	-2.929486000
1	-0.097388000	4.168787000	-1.476396000
1	0.310211000	3.976790000	0.214807000
1	3.504289000	7.193374000	-1.488714000
1	1.966383000	7.879137000	-1.964936000
1	2.420212000	2.772915000	-0.458479000
1	1.939379000	2.922171000	-2.136525000
1	4.038854000	6.732739000	-3.882339000
1	2.474341000	7.352881000	-4.351497000
1	-0.156502000	1.662462000	-1.644048000
1	1.338447000	0.735892000	-1.419217000
1	3.119396000	9.605945000	-3.442338000
1	4.076031000	9.058654000	-4.800647000

6	5.141675000	9.079457000	-2.951005000
1	4.944153000	8.947612000	-1.885560000
1	5.864530000	8.305867000	-3.226403000
6	5.754172000	10.447991000	-3.181216000
1	6.667541000	10.577543000	-2.600385000
1	5.061029000	11.240743000	-2.894893000
1	6.005094000	10.592454000	-4.233435000
**1- $CTA^+/(C_{12}\text{-}OSO_3)^-$ 1:1 ΔG=0.0**			
7	2.285864000	3.403069000	1.654424000
6	2.772591000	2.289650000	0.768379000
6	3.782403000	2.671858000	-0.287492000
6	3.978419000	1.492189000	-1.226218000
6	5.101112000	1.715267000	-2.222531000
6	5.201684000	0.629567000	-3.282412000
6	5.437002000	-0.764956000	-2.729559000
6	5.650527000	-1.803775000	-3.813886000
6	5.781499000	-3.215235000	-3.273807000
6	1.406211000	2.797505000	2.690805000
6	1.485473000	4.383322000	0.874820000
6	3.418460000	4.092464000	2.321009000
6	6.050249000	-4.245935000	-4.353456000
6	6.103904000	-5.669197000	-3.831386000
6	6.443017000	-6.685321000	-4.905482000
6	6.450026000	-8.117086000	-4.404689000
6	6.830573000	-9.122806000	-5.475029000
6	6.795185000	-10.560196000	-4.991768000
6	7.167166000	-11.566427000	-6.065123000
6	7.071780000	-13.002296000	-5.585728000
1	1.877250000	1.874164000	0.314156000
1	3.195228000	1.542466000	1.437214000
1	3.448677000	3.534145000	-0.864705000
1	4.736012000	2.934950000	0.171301000
1	3.044343000	1.303985000	-1.762691000
1	4.180071000	0.596274000	-0.635681000
1	4.954239000	2.679938000	-2.715232000
1	6.050033000	1.787445000	-1.682900000
1	4.285254000	0.625881000	-3.880749000
1	6.013726000	0.881315000	-3.969727000
1	6.305777000	-0.750535000	-2.063643000
1	4.586315000	-1.068454000	-2.114401000
1	6.546072000	-1.549394000	-4.389085000
1	4.814947000	-1.765351000	-4.519859000
1	4.865295000	-3.481893000	-2.737341000
1	6.587462000	-3.248141000	-2.534301000
1	0.601828000	2.268162000	2.190316000
1	1.012879000	3.592679000	3.315901000
1	1.997341000	2.108365000	3.285477000
1	0.677642000	3.849192000	0.384597000
1	2.121639000	4.867355000	0.143684000
1	1.088802000	5.122713000	1.563296000
1	3.013540000	4.810099000	3.027476000

1	4.017228000	3.350018000	2.839752000
1	4.014162000	4.604600000	1.574260000
1	5.275418000	-4.173438000	-5.123004000
1	6.995391000	-4.007161000	-4.850849000
1	6.843769000	-5.733436000	-3.027612000
1	5.139929000	-5.924679000	-3.380295000
1	7.423200000	-6.445710000	-5.329279000
1	5.726179000	-6.594510000	-5.727425000
1	5.459957000	-8.366515000	-4.011269000
1	7.144262000	-8.204249000	-3.563240000
1	6.154185000	-9.012703000	-6.328255000
1	7.832397000	-8.890548000	-5.849422000
1	5.793463000	-10.789555000	-4.616520000
1	7.472843000	-10.674684000	-4.139963000
1	8.182629000	-11.361299000	-6.414552000
1	6.513061000	-11.426733000	-6.930070000
1	7.351470000	-13.705954000	-6.369817000
1	6.054119000	-13.239950000	-5.271614000
1	7.730002000	-13.174859000	-4.732766000
16	-0.321744000	-0.030919000	0.008135000
8	0.730415000	-0.624840000	-1.073332000
8	-0.393357000	1.410825000	-0.247351000
8	-1.589840000	-0.720832000	-0.235423000
8	0.320063000	-0.381485000	1.271345000
6	1.835064000	-3.647289000	-4.069568000
6	1.805756000	-5.123729000	-3.720132000
6	1.170980000	-2.778013000	-3.016928000
6	2.297641000	-6.025861000	-4.836591000
6	1.313613000	-1.292764000	-3.296433000
6	2.307593000	-7.493915000	-4.453134000
6	0.412351000	-0.425224000	-2.456261000
6	2.720235000	-8.415165000	-5.586222000
1	2.871849000	-3.329301000	-4.210716000
1	1.334085000	-3.489618000	-5.029472000
1	2.409926000	-5.291591000	-2.822989000
1	0.783116000	-5.411005000	-3.456227000
1	1.591675000	-3.006438000	-2.035121000
1	0.108755000	-3.034876000	-2.962188000
1	3.305217000	-5.723727000	-5.136217000
1	1.662393000	-5.885767000	-5.716538000
1	1.058479000	-1.086902000	-4.338879000
1	2.352077000	-0.982614000	-3.162316000
1	2.981922000	-7.639408000	-3.603299000
1	1.310997000	-7.780942000	-4.103140000
1	0.546032000	0.629738000	-2.697285000
1	-0.631968000	-0.694457000	-2.619796000
1	3.715618000	-8.131994000	-5.941722000
1	2.040204000	-8.266438000	-6.428483000
6	2.736445000	-9.878620000	-5.180476000
1	3.392846000	-9.991055000	-4.313278000
1	1.736695000	-10.171619000	-4.844209000

6	3.197546000	-10.830030000	-6.273679000
1	3.339349000	-11.825504000	-5.842876000
1	4.180623000	-10.514477000	-6.635058000
6	2.248105000	-10.944496000	-7.453709000
1	1.258982000	-11.237282000	-7.090249000
1	2.123599000	-9.968869000	-7.927587000
6	2.728617000	-11.945645000	-8.487698000
1	2.036461000	-12.019136000	-9.326651000
1	2.830688000	-12.940386000	-8.050904000
1	3.703706000	-11.658172000	-8.884378000
**2- $CTA^+/(C_{12}\text{-}OSO_3)^-$ 1:1 ΔG=0.51**			
7	4.562020000	1.124780000	3.255208000
6	3.477973000	0.094559000	3.094941000
6	2.501075000	-0.017783000	4.240440000
6	1.413108000	-1.018371000	3.886840000
6	0.383396000	-1.172519000	4.990048000
6	-0.771864000	-2.073583000	4.598200000
6	-1.842645000	-2.173748000	5.667998000
6	-3.006598000	-3.061154000	5.269835000
6	-4.080058000	-3.157430000	6.337926000
6	5.420503000	1.063121000	2.040319000
6	5.396161000	0.842325000	4.450307000
6	3.987778000	2.492800000	3.357883000
6	-5.220682000	-4.083106000	5.951593000
6	-6.265806000	-4.276691000	7.039665000
6	-7.078913000	-3.033763000	7.354067000
6	-8.114891000	-3.256191000	8.440688000
6	-8.952580000	-2.020036000	8.711873000
6	-10.008077000	-2.196451000	9.793383000
6	-9.471360000	-2.541839000	11.176478000
6	-8.490633000	-1.522718000	11.728045000
1	3.992394000	-0.851786000	2.938520000
1	2.958944000	0.357366000	2.176179000
1	3.005825000	-0.341104000	5.150929000
1	2.036012000	0.945939000	4.447784000
1	1.863967000	-1.989867000	3.667594000
1	0.914538000	-0.695707000	2.969469000
1	0.867706000	-1.565363000	5.888630000
1	-0.007098000	-0.187302000	5.260056000
1	-1.220541000	-1.697783000	3.673966000
1	-0.392401000	-3.073864000	4.368426000
1	-1.397306000	-2.554686000	6.592138000
1	-2.216321000	-1.171642000	5.899388000
1	-2.633712000	-4.064105000	5.040197000
1	-3.450500000	-2.681140000	4.344251000
1	-4.462765000	-2.155193000	6.547120000
1	-3.630674000	-3.514946000	7.269899000
1	5.842389000	0.065864000	1.967187000
1	6.211206000	1.799520000	2.141748000
1	4.802898000	1.283585000	1.175679000
1	4.792163000	0.954485000	5.342939000

1	6.215924000	1.553421000	4.468799000
1	5.778442000	-0.170896000	4.375073000
1	3.342281000	2.657519000	2.500975000
1	3.430562000	2.579391000	4.283007000
1	4.808180000	3.203436000	3.356961000
1	-5.705325000	-3.702944000	5.046701000
1	-4.802201000	-5.057581000	5.685586000
1	-6.949688000	-5.075185000	6.739264000
1	-5.772793000	-4.626524000	7.952324000
1	-7.575903000	-2.690211000	6.440868000
1	-6.416641000	-2.222035000	7.663231000
1	-7.604482000	-3.570894000	9.354698000
1	-8.772459000	-4.084491000	8.157613000
1	-8.291798000	-1.188970000	8.971731000
1	-9.450215000	-1.723076000	7.785058000
1	-10.708364000	-2.976921000	9.482568000
1	-10.590980000	-1.274164000	9.865491000
1	-8.998492000	-3.526203000	11.156909000
1	-10.318509000	-2.631539000	11.860264000
1	-7.575061000	-1.484889000	11.137869000
1	-8.210613000	-1.764430000	12.753494000
1	-8.924262000	-0.521014000	11.727990000
16	1.466644000	1.576447000	-0.107311000
8	0.772364000	1.955961000	1.308760000
8	2.794761000	2.164515000	0.050125000
8	1.466070000	0.113920000	-0.177048000
8	0.647858000	2.206183000	-1.144023000
6	-2.824184000	1.728662000	4.633049000
6	-4.220329000	1.235803000	4.965275000
6	-2.399216000	1.407153000	3.212741000
6	-4.621206000	1.528885000	6.400292000
6	-1.001075000	1.902051000	2.892749000
6	-6.026449000	1.078710000	6.773448000
6	-0.573639000	1.507517000	1.504096000
6	-7.129065000	1.845465000	6.061084000
1	-2.777404000	2.810658000	4.788654000
1	-2.106432000	1.288340000	5.331301000
1	-4.929426000	1.695972000	4.273411000
1	-4.276707000	0.156279000	4.790566000
1	-3.113116000	1.847527000	2.510933000
1	-2.446026000	0.325222000	3.061119000
1	-4.530047000	2.603763000	6.586654000
1	-3.903015000	1.043723000	7.067349000
1	-0.290709000	1.489390000	3.610702000
1	-0.957959000	2.988948000	2.988718000
1	-6.135511000	0.010785000	6.559594000
1	-6.149026000	1.186817000	7.852990000
1	-0.607791000	0.424320000	1.381071000
1	-1.214843000	1.965483000	0.749864000
1	-7.034477000	1.698598000	4.983662000
1	-6.989677000	2.917389000	6.236048000

6	-8.538679000	1.453104000	6.479295000
1	-9.250344000	1.936615000	5.804356000
1	-8.671842000	0.375613000	6.340012000
6	-8.902853000	1.824187000	7.906732000
1	-8.674270000	2.882109000	8.072153000
1	-8.285045000	1.263696000	8.612545000
6	-10.366562000	1.576914000	8.224845000
1	-10.983824000	2.166768000	7.541790000
1	-10.606978000	0.529596000	8.024969000
6	-10.730234000	1.915472000	9.657808000
1	-10.492775000	2.955620000	9.887057000
1	-10.183429000	1.286281000	10.361768000
1	-11.794494000	1.768822000	9.843613000
**3- $CTA^+/(C_{12}\text{-}OSO_3)^-$ 1:1 ΔG=1.18**			
7	-2.747303000	-2.626099000	-1.512085000
6	-1.855231000	-1.791395000	-2.390164000
6	-2.562647000	-0.822714000	-3.311226000
6	-1.565107000	-0.174360000	-4.264518000
6	-1.075910000	-1.098840000	-5.365526000
6	-0.011980000	-0.470337000	-6.245187000
6	0.475109000	-1.402711000	-7.339352000
6	1.692300000	-0.881490000	-8.079601000
6	2.260560000	-1.879131000	-9.070688000
6	-1.869957000	-3.425429000	-0.613818000
6	-3.572052000	-3.557285000	-2.322894000
6	-3.631407000	-1.770898000	-0.678617000
6	3.506447000	-1.381833000	-9.779635000
6	4.100635000	-2.415469000	-10.719950000
6	5.402385000	-1.992911000	-11.382349000
6	5.270930000	-0.826160000	-12.345457000
6	6.575794000	-0.486653000	-13.042921000
6	6.445897000	0.674819000	-14.012347000
6	7.750952000	1.090286000	-14.674480000
6	8.343107000	0.034587000	-15.591233000
1	-1.256911000	-2.507129000	-2.948024000
1	-1.197790000	-1.247494000	-1.716057000
1	-3.336139000	-1.322843000	-3.894607000
1	-3.049512000	-0.046791000	-2.722230000
1	-0.712789000	0.204321000	-3.693419000
1	-2.039345000	0.697976000	-4.717749000
1	-0.664452000	-2.015288000	-4.935539000
1	-1.928054000	-1.406020000	-5.979002000
1	-0.394723000	0.453026000	-6.689938000
1	0.836669000	-0.178117000	-5.619261000
1	0.720263000	-2.373176000	-6.898012000
1	-0.336003000	-1.591024000	-8.048937000
1	2.467292000	-0.624012000	-7.350982000
1	1.439176000	0.048638000	-8.597450000
1	1.497365000	-2.133888000	-9.812514000
1	2.495394000	-2.809311000	-8.543096000
1	-1.294992000	-2.741514000	0.001981000

1	-1.214317000	-4.036116000	-1.226710000
1	-2.498055000	-4.055640000	0.007889000
1	-4.255481000	-2.988992000	-2.942220000
1	-4.132710000	-4.192098000	-1.644112000
1	-2.909814000	-4.156804000	-2.939674000
1	-4.331629000	-1.250094000	-1.321261000
1	-4.171925000	-2.414276000	0.008582000
1	-3.012574000	-1.066607000	-0.132597000
1	3.265196000	-0.467498000	-10.327009000
1	4.257695000	-1.103758000	-9.033386000
1	4.274240000	-3.336194000	-10.155803000
1	3.366878000	-2.666133000	-11.492603000
1	5.816134000	-2.848243000	-11.923785000
1	6.134870000	-1.741355000	-10.608648000
1	4.910198000	0.058608000	-11.815454000
1	4.511008000	-1.064507000	-13.096587000
1	7.334344000	-0.243930000	-12.291732000
1	6.938294000	-1.373912000	-13.567737000
1	6.032448000	1.533755000	-13.476741000
1	5.715954000	0.417862000	-14.786622000
1	8.478292000	1.351604000	-13.900683000
1	7.577767000	2.002845000	-15.249299000
1	7.626282000	-0.256836000	-16.360993000
1	8.625772000	-0.864416000	-15.044359000
1	9.236051000	0.407822000	-16.093000000
16	-0.424088000	0.317188000	0.853303000
8	0.757211000	-0.421953000	0.023566000
8	-1.049030000	-0.807841000	1.543929000
8	0.238834000	1.275589000	1.739298000
8	-1.282098000	0.953218000	-0.148541000
6	3.139160000	-2.155520000	-3.232724000
6	2.563718000	-3.008041000	-4.349567000
6	2.070129000	-1.371204000	-2.493608000
6	3.578725000	-3.870278000	-5.083641000
6	2.643800000	-0.439580000	-1.443017000
6	4.626638000	-3.090005000	-5.858643000
6	1.616766000	0.420878000	-0.754690000
6	5.564748000	-3.984520000	-6.649007000
1	3.679705000	-2.793278000	-2.526272000
1	3.873731000	-1.456203000	-3.639372000
1	1.784680000	-3.653164000	-3.933611000
1	2.064969000	-2.353337000	-5.068880000
1	1.365043000	-2.064558000	-2.029395000
1	1.493854000	-0.783205000	-3.213786000
1	3.045715000	-4.524525000	-5.779404000
1	4.078827000	-4.530904000	-4.368239000
1	3.361684000	0.238229000	-1.910674000
1	3.196231000	-1.008407000	-0.691524000
1	4.127878000	-2.395544000	-6.542229000
1	5.216736000	-2.475254000	-5.174658000
1	2.095918000	1.139140000	-0.089928000

1	1.012364000	0.962911000	-1.484010000
1	4.977484000	-4.588548000	-7.345457000
1	6.050227000	-4.689530000	-5.966236000
6	6.631512000	-3.208755000	-7.402485000
1	6.152900000	-2.559520000	-8.142043000
1	7.140088000	-2.543435000	-6.699331000
6	7.673375000	-4.075933000	-8.092491000
1	8.163455000	-4.711565000	-7.347814000
1	8.455615000	-3.430059000	-8.501540000
6	7.131515000	-4.947081000	-9.212479000
1	6.381828000	-5.636162000	-8.818851000
1	6.616138000	-4.313126000	-9.938145000
6	8.220517000	-5.737727000	-9.913313000
1	7.814306000	-6.353961000	-10.715527000
1	8.735299000	-6.398659000	-9.214034000
1	8.967467000	-5.072446000	-10.349418000

4- $CTA^+/(C_{12}\text{-}OSO_3)^-$ 1:1 ΔG=2.03

7	-0.805762000	-4.447893000	-2.197044000
6	0.289419000	-3.724179000	-2.934831000
6	-0.166047000	-2.851893000	-4.084617000
6	0.946211000	-1.900337000	-4.506831000
6	2.186821000	-2.586280000	-5.055273000
6	3.299182000	-1.617793000	-5.429896000
6	3.918027000	-0.913931000	-4.234723000
6	5.102510000	-0.033781000	-4.587923000
6	5.689959000	0.657414000	-3.372044000
6	-1.646824000	-5.239301000	-3.129669000
6	-1.659777000	-3.495680000	-1.440181000
6	-0.156817000	-5.370288000	-1.225556000
6	6.885720000	1.540825000	-3.673123000
6	7.447055000	2.197991000	-2.426167000
6	8.653485000	3.081223000	-2.680290000
6	9.219752000	3.683558000	-1.407305000
6	10.426779000	4.571497000	-1.650284000
6	11.014415000	5.190890000	-0.391176000
6	11.622925000	4.186297000	0.573311000
6	12.342354000	4.846762000	1.734545000
1	0.962871000	-4.502995000	-3.283113000
1	0.807206000	-3.133206000	-2.182120000
1	-0.468119000	-3.468185000	-4.932246000
1	-1.029887000	-2.255321000	-3.794591000
1	1.206639000	-1.278783000	-3.649529000
1	0.550069000	-1.227669000	-5.270834000
1	2.582165000	-3.295313000	-4.323269000
1	1.904531000	-3.175690000	-5.931730000
1	4.082008000	-2.166761000	-5.959711000
1	2.915605000	-0.873901000	-6.135036000
1	3.166436000	-0.302062000	-3.730771000
1	4.235602000	-1.665650000	-3.504928000
1	4.793770000	0.717266000	-5.321577000

1	5.874652000	-0.637843000	-5.074231000
1	5.981535000	-0.098930000	-2.636833000
1	4.911996000	1.259406000	-2.892652000
1	-2.347737000	-5.825256000	-2.543473000
1	-1.000930000	-5.892542000	-3.708282000
1	-2.186801000	-4.564622000	-3.783956000
1	-2.400179000	-4.067678000	-0.889947000
1	-2.156686000	-2.825263000	-2.130872000
1	-1.030202000	-2.938153000	-0.754744000
1	0.485838000	-4.784746000	-0.575603000
1	-0.931356000	-5.867720000	-0.650881000
1	0.426302000	-6.098734000	-1.779878000
1	7.667433000	0.944896000	-4.154329000
1	6.599089000	2.311060000	-4.395810000
1	6.661599000	2.791833000	-1.949770000
1	7.716956000	1.420906000	-1.704369000
1	8.379702000	3.882362000	-3.373884000
1	9.432431000	2.497043000	-3.180233000
1	9.484048000	2.873243000	-0.723425000
1	8.441700000	4.265055000	-0.903031000
1	11.203690000	3.992015000	-2.159227000
1	10.141713000	5.370101000	-2.340621000
1	10.241331000	5.767698000	0.126997000
1	11.787233000	5.909376000	-0.678680000
1	12.323068000	3.548087000	0.026355000
1	10.845909000	3.524488000	0.961182000
1	13.152637000	5.486006000	1.380296000
1	12.771822000	4.106448000	2.409757000
1	11.661187000	5.470144000	2.314642000
16	1.535851000	-1.381698000	0.278705000
8	2.919574000	-0.716922000	0.796541000
8	1.247262000	-0.766632000	-1.017936000
8	1.787294000	-2.822192000	0.183344000
8	0.607056000	-1.007419000	1.341564000
6	6.271481000	2.006245000	1.331697000
6	6.170769000	3.520067000	1.317250000
6	5.089532000	1.325742000	0.666575000
6	7.297160000	4.211648000	2.062662000
6	5.236630000	-0.182708000	0.601774000
6	7.230702000	5.724465000	1.966539000
6	4.070060000	-0.885753000	-0.042732000
6	8.287716000	6.433335000	2.792825000
1	7.196230000	1.702668000	0.831899000
1	6.352980000	1.656686000	2.365428000
1	6.155882000	3.867704000	0.279970000
1	5.214046000	3.823076000	1.753703000
1	4.967613000	1.714020000	-0.348404000
1	4.175985000	1.583541000	1.206959000
1	8.259932000	3.866735000	1.672962000
1	7.268554000	3.912865000	3.115024000
1	5.390130000	-0.591790000	1.603258000

1	6.126164000	-0.437785000	0.021053000
1	7.331810000	6.022170000	0.917874000
1	6.239791000	6.063051000	2.284763000
1	3.854449000	-0.463640000	-1.024663000
1	4.270868000	-1.951475000	-0.152460000
1	9.274512000	6.067647000	2.497998000
1	8.163508000	6.164049000	3.846661000
6	8.231266000	7.943980000	2.649710000
1	8.443841000	8.213583000	1.611791000
1	7.207825000	8.277963000	2.841402000
6	9.163880000	8.700404000	3.585578000
1	8.859773000	8.504922000	4.617710000
1	9.027015000	9.773580000	3.424953000
6	10.643011000	8.369214000	3.441532000
1	11.199737000	8.954441000	4.176939000
1	10.813321000	7.322304000	3.701526000
6	11.202316000	8.645196000	2.058106000
1	10.732034000	8.016097000	1.302558000
1	12.274836000	8.453187000	2.023012000
1	11.038997000	9.685886000	1.771845000

5- $CTA^+/(C_{12}\text{-}OSO_3)^-$ 1:1 ΔG=2.15

7	-2.040556000	-3.167365000	2.283592000
6	-0.918540000	-3.869657000	1.568976000
6	0.308663000	-4.166577000	2.395908000
6	1.381328000	-4.765263000	1.501180000
6	2.679305000	-5.033142000	2.237680000
6	3.774371000	-5.548285000	1.323149000
6	5.083008000	-5.822853000	2.039101000
6	6.173685000	-6.324326000	1.110495000
6	7.495231000	-6.566140000	1.818421000
6	-3.136261000	-2.959943000	1.298289000
6	-1.603544000	-1.838223000	2.787465000
6	-2.553582000	-3.988004000	3.408961000
6	8.581746000	-7.171803000	0.941526000
6	9.032456000	-6.272851000	-0.197089000
6	10.106601000	-6.886220000	-1.086652000
6	11.403354000	-7.265742000	-0.383630000
6	12.076841000	-6.100920000	0.321016000
6	13.459382000	-6.414980000	0.871226000
6	13.478762000	-7.471033000	1.963371000
6	14.855960000	-7.645478000	2.575594000
1	-0.663080000	-3.225426000	0.731820000
1	-1.348905000	-4.790309000	1.178825000
1	0.699438000	-3.254979000	2.846183000
1	0.077919000	-4.861282000	3.203765000
1	1.571772000	-4.083036000	0.669356000
1	1.012973000	-5.695781000	1.060416000
1	3.015377000	-4.111013000	2.720026000
1	2.502865000	-5.753954000	3.041390000
1	3.432655000	-6.463044000	0.829058000

1	3.946585000	-4.817644000	0.526601000
1	5.422067000	-4.907770000	2.533011000
1	4.916398000	-6.555924000	2.834290000
1	6.308446000	-5.599302000	0.304198000
1	5.844228000	-7.253451000	0.634142000
1	7.320192000	-7.228768000	2.670514000
1	7.856772000	-5.621775000	2.235997000
1	-2.753619000	-2.351806000	0.485007000
1	-3.957747000	-2.455384000	1.796944000
1	-3.457627000	-3.929151000	0.930417000
1	-2.473289000	-1.329613000	3.191435000
1	-1.182961000	-1.279595000	1.957790000
1	-0.864354000	-1.974948000	3.567834000
1	-2.842524000	-4.960671000	3.022763000
1	-1.777734000	-4.093012000	4.158144000
1	-3.413226000	-3.481662000	3.836572000
1	9.439826000	-7.413313000	1.572621000
1	8.227718000	-8.123307000	0.531009000
1	8.176496000	-6.023227000	-0.826456000
1	9.387626000	-5.324854000	0.216636000
1	10.336998000	-6.180614000	-1.889881000
1	9.695436000	-7.775907000	-1.572004000
1	12.092688000	-7.677458000	-1.126492000
1	11.214594000	-8.071248000	0.328367000
1	12.156156000	-5.264222000	-0.379387000
1	11.441982000	-5.752059000	1.139563000
1	13.896525000	-5.495018000	1.269921000
1	14.113733000	-6.732640000	0.053054000
1	13.136955000	-8.427327000	1.562664000
1	12.763914000	-7.192499000	2.742660000
1	15.205929000	-6.711745000	3.018941000
1	15.584188000	-7.945964000	1.820385000
1	14.853828000	-8.405794000	3.356785000
16	0.788268000	-1.035292000	-0.713147000
8	1.234448000	-1.056029000	0.844734000
8	-0.663150000	-1.167524000	-0.603467000
8	1.232239000	0.253946000	-1.245967000
8	1.450812000	-2.179087000	-1.341702000
6	5.162737000	-1.667184000	2.876846000
6	6.582321000	-1.386560000	3.335147000
6	4.275832000	-0.435798000	2.949684000
6	7.486250000	-2.604200000	3.289293000
6	2.821104000	-0.670263000	2.573409000
6	8.895652000	-2.321264000	3.775602000
6	2.640859000	-1.007391000	1.114452000
6	9.801806000	-3.537666000	3.741694000
1	5.186921000	-2.059108000	1.856591000
1	4.731386000	-2.457277000	3.498310000
1	7.011526000	-0.593484000	2.715136000
1	6.557972000	-0.994548000	4.356634000
1	4.691055000	0.349485000	2.311049000

1	4.305798000	-0.044043000	3.968998000
1	7.524875000	-2.989778000	2.265769000
1	7.048902000	-3.401552000	3.897757000
1	2.244502000	0.231608000	2.785371000
1	2.401071000	-1.470552000	3.186633000
1	9.335633000	-1.524899000	3.167468000
1	8.851324000	-1.933983000	4.798303000
1	3.099232000	-0.239823000	0.489328000
1	3.076469000	-1.973496000	0.861449000
1	9.867448000	-3.913477000	2.715728000
1	9.349946000	-4.341995000	4.330589000
6	11.201031000	-3.263527000	4.263158000
1	11.644031000	-2.450345000	3.682780000
1	11.139360000	-2.905726000	5.295945000
6	12.094621000	-4.490095000	4.208778000
1	12.183913000	-4.818448000	3.169836000
1	11.601784000	-5.306034000	4.744898000
6	13.487108000	-4.291518000	4.786870000
1	13.402416000	-3.956077000	5.824426000
1	13.992408000	-5.259499000	4.820610000
6	14.346178000	-3.314017000	4.004850000
1	14.431583000	-3.621914000	2.961119000
1	15.353197000	-3.258479000	4.418730000
1	13.929159000	-2.307323000	4.019601000
**1- $CTA^+/(C_{12}\text{-}OSO_3)^-$ 2:1 ΔG=0.0**			
7	2.083955000	1.913424000	3.327582000
6	2.633349000	2.262674000	1.970397000
6	3.985563000	1.678583000	1.638906000
6	4.383863000	2.057483000	0.221724000
6	5.782869000	1.581047000	-0.122725000
6	6.163888000	1.860060000	-1.565737000
6	7.621241000	1.572338000	-1.893451000
6	8.031578000	0.121094000	-1.713375000
6	9.468820000	-0.137779000	-2.131568000
6	2.989984000	2.379810000	4.407316000
6	1.864586000	0.448594000	3.457216000
6	0.770606000	2.598771000	3.471697000
6	9.907031000	-1.589314000	-2.008729000
6	9.959259000	-2.115415000	-0.585149000
6	10.495417000	-3.532915000	-0.498890000
6	10.502450000	-4.088061000	0.913188000
6	11.074793000	-5.492389000	0.993589000
6	11.038784000	-6.111133000	2.382931000
6	9.639568000	-6.419448000	2.888617000
6	9.639487000	-7.113223000	4.237506000
1	2.674499000	3.350147000	1.942601000
1	1.883891000	1.932122000	1.256071000
1	4.742755000	2.048041000	2.330735000
1	3.971479000	0.591845000	1.718991000
1	4.329403000	3.142804000	0.101391000
1	3.665581000	1.629668000	-0.482374000

1	6.501579000	2.067403000	0.544060000
1	5.852195000	0.510013000	0.079262000
1	5.521109000	1.270259000	-2.225536000
1	5.953060000	2.909165000	-1.790663000
1	7.815088000	1.868616000	-2.928155000
1	8.261146000	2.205747000	-1.270800000
1	7.365584000	-0.518274000	-2.301270000
1	7.894500000	-0.170921000	-0.671336000
1	10.136970000	0.492158000	-1.535415000
1	9.594940000	0.183470000	-3.169329000
1	3.919958000	1.825694000	4.360559000
1	2.502520000	2.201010000	5.360231000
1	3.173086000	3.441254000	4.272015000
1	1.389438000	0.263031000	4.415484000
1	2.819003000	-0.062246000	3.417837000
1	1.218415000	0.126019000	2.647092000
1	0.108016000	2.235577000	2.692500000
1	0.367360000	2.364264000	4.451648000
1	0.926252000	3.668588000	3.375820000
1	9.234735000	-2.219887000	-2.597937000
1	10.897958000	-1.698215000	-2.458381000
1	10.586136000	-1.454306000	0.021905000
1	8.962110000	-2.086281000	-0.139323000
1	11.510314000	-3.561466000	-0.907826000
1	9.891690000	-4.186821000	-1.137150000
1	9.480553000	-4.079195000	1.298505000
1	11.081615000	-3.424835000	1.563540000
1	10.528340000	-6.139191000	0.301340000
1	12.108357000	-5.471022000	0.637256000
1	11.544114000	-5.447447000	3.092133000
1	11.615851000	-7.040173000	2.372165000
1	9.130709000	-7.049062000	2.154480000
1	9.055034000	-5.499841000	2.956410000
1	10.115112000	-6.491882000	4.998251000
1	10.186158000	-8.056639000	4.192839000
1	8.625264000	-7.331938000	4.572880000
7	-0.741288000	0.745072000	-4.912930000
6	0.605454000	0.231188000	-4.478467000
6	1.763605000	1.180438000	-4.681924000
6	3.047322000	0.601811000	-4.099659000
6	3.509062000	-0.691506000	-4.749916000
6	4.871820000	-1.144037000	-4.258883000
6	5.295009000	-2.484577000	-4.831129000
6	6.590808000	-3.010330000	-4.242627000
6	7.033466000	-4.330561000	-4.846474000
6	-0.749607000	1.064318000	-6.363137000
6	-1.135465000	1.947778000	-4.133005000
6	-1.734607000	-0.332595000	-4.651621000
6	8.244428000	-4.930457000	-4.155719000
6	8.727875000	-6.215321000	-4.806621000
6	9.870203000	-6.897295000	-4.067139000

6	9.449166000	-7.548493000	-2.761509000
6	10.589900000	-8.217272000	-2.017130000
6	10.133035000	-8.908616000	-0.745793000
6	11.265631000	-9.489874000	0.078798000
6	10.778972000	-10.156747000	1.351789000
1	0.753049000	-0.687736000	-5.039941000
1	0.495115000	-0.022701000	-3.426127000
1	1.913368000	1.379962000	-5.743365000
1	1.563261000	2.133543000	-4.194430000
1	2.917947000	0.443805000	-3.025232000
1	3.830843000	1.354592000	-4.206080000
1	2.787848000	-1.488374000	-4.551754000
1	3.535072000	-0.563087000	-5.836535000
1	5.620629000	-0.385827000	-4.506783000
1	4.854093000	-1.208429000	-3.168373000
1	4.501481000	-3.216722000	-4.651582000
1	5.392686000	-2.401867000	-5.918084000
1	6.467546000	-3.133066000	-3.162251000
1	7.382367000	-2.266843000	-4.375369000
1	7.250349000	-4.188407000	-5.909749000
1	6.205826000	-5.045200000	-4.797556000
1	-0.086056000	1.900239000	-6.550477000
1	-1.763540000	1.327647000	-6.647109000
1	-0.420240000	0.187812000	-6.912490000
1	-1.072715000	1.709413000	-3.076397000
1	-2.153474000	2.207264000	-4.406432000
1	-0.471867000	2.768220000	-4.378801000
1	-1.720595000	-0.557698000	-3.590511000
1	-2.714451000	0.022076000	-4.954629000
1	-1.456116000	-1.206814000	-5.231547000
1	9.062006000	-4.203198000	-4.154138000
1	7.994888000	-5.110181000	-3.106849000
1	7.890641000	-6.915348000	-4.893356000
1	9.041934000	-5.990207000	-5.829217000
1	10.662966000	-6.167712000	-3.872506000
1	10.311172000	-7.661198000	-4.712963000
1	8.670605000	-8.289389000	-2.969972000
1	8.992840000	-6.804548000	-2.104996000
1	11.081668000	-8.943547000	-2.671600000
1	11.346841000	-7.466409000	-1.769692000
1	9.424914000	-9.703559000	-1.000502000
1	9.576406000	-8.195734000	-0.131049000
1	11.970655000	-8.692553000	0.330099000
1	11.822575000	-10.211094000	-0.525606000
1	10.242331000	-9.445608000	1.982238000
1	11.606439000	-10.561168000	1.935019000
1	10.096585000	-10.978193000	1.126866000
16	-0.063871000	0.470353000	-0.386497000
8	1.352185000	-0.304805000	-0.271623000
8	-0.620722000	0.492451000	0.967997000
8	0.352012000	1.779900000	-0.879627000

8	-0.875824000	-0.298619000	-1.331490000
6	5.107609000	-2.457305000	-0.309526000
6	5.234515000	-3.970189000	-0.279549000
6	3.722378000	-1.948849000	-0.675767000
6	6.654182000	-4.424802000	0.010625000
6	2.685459000	-2.168768000	0.411036000
6	6.851375000	-5.931964000	-0.001825000
6	1.302027000	-1.704815000	0.035341000
6	6.167121000	-6.667617000	1.137453000
1	5.396390000	-2.048168000	0.664081000
1	5.830948000	-2.062752000	-1.027056000
1	4.551843000	-4.386607000	0.463297000
1	4.920125000	-4.373916000	-1.247915000
1	3.388430000	-2.433999000	-1.598483000
1	3.782099000	-0.881841000	-0.895935000
1	6.968664000	-4.028468000	0.981480000
1	7.321286000	-3.974221000	-0.729712000
1	2.998966000	-1.664137000	1.327444000
1	2.602516000	-3.230095000	0.651362000
1	6.501539000	-6.334619000	-0.958017000
1	7.922234000	-6.147942000	0.042740000
1	0.934949000	-2.236875000	-0.843402000
1	0.606578000	-1.865523000	0.859546000
1	5.088396000	-6.495325000	1.105710000
1	6.515500000	-6.252733000	2.088826000
6	6.430843000	-8.162272000	1.117320000
1	6.043986000	-8.586524000	0.185553000
1	7.510047000	-8.337787000	1.103681000
6	5.824140000	-8.899254000	2.296003000
1	4.738982000	-8.756082000	2.296941000
1	6.189849000	-8.455651000	3.227682000
6	6.134805000	-10.385002000	2.297818000
1	5.777925000	-10.829094000	1.364616000
1	7.219732000	-10.522870000	2.304260000
6	5.522196000	-11.116052000	3.477350000
1	4.435337000	-11.020139000	3.476294000
1	5.888664000	-10.708855000	4.420955000
1	5.762255000	-12.179189000	3.457520000
**2- $CTA^+/(C_{12}\text{-}OSO_3)^-$ 2:1 ΔG=1.60**			
7	-4.246163000	1.268230000	0.243283000
6	-3.249484000	2.350669000	-0.072569000
6	-2.789609000	2.423858000	-1.508795000
6	-1.835907000	3.600286000	-1.654235000
6	-1.166287000	3.687015000	-3.014015000
6	-2.119288000	3.873884000	-4.181477000
6	-1.384845000	4.139733000	-5.481698000
6	-2.291899000	4.296470000	-6.687637000
6	-1.518622000	4.617799000	-7.953082000
6	-3.727917000	-0.065778000	-0.157388000
6	-5.544381000	1.522525000	-0.430851000
6	-4.463307000	1.274071000	1.714570000

6	-2.394547000	4.797808000	-9.179862000
6	-1.636585000	5.260125000	-10.414837000
6	-0.607628000	4.271323000	-10.934113000
6	0.058649000	4.733281000	-12.217090000
6	1.101180000	3.761315000	-12.737595000
6	1.748796000	4.222067000	-14.031649000
6	2.861547000	3.315117000	-14.534905000
6	2.395075000	1.926153000	-14.933532000
1	-2.399329000	2.163995000	0.579137000
1	-3.724265000	3.282683000	0.229944000
1	-2.267872000	1.508418000	-1.786427000
1	-3.634270000	2.546923000	-2.185022000
1	-1.062916000	3.526373000	-0.885570000
1	-2.379083000	4.527614000	-1.457248000
1	-0.463344000	4.524296000	-2.997919000
1	-0.563146000	2.788918000	-3.178998000
1	-2.751114000	2.989604000	-4.297659000
1	-2.793350000	4.709173000	-3.967614000
1	-0.784016000	5.046592000	-5.368808000
1	-0.675585000	3.327154000	-5.668518000
1	-3.020150000	5.089921000	-6.493842000
1	-2.869915000	3.378619000	-6.833005000
1	-0.788126000	3.824589000	-8.130912000
1	-0.940316000	5.532448000	-7.794251000
1	-4.411708000	-0.820458000	0.218007000
1	-2.738588000	-0.194462000	0.269007000
1	-3.679220000	-0.118541000	-1.238679000
1	-5.904806000	2.501306000	-0.129807000
1	-6.244289000	0.752321000	-0.122597000
1	-5.404121000	1.484511000	-1.504526000
1	-5.202626000	0.517782000	1.958195000
1	-3.520242000	1.052052000	2.203294000
1	-4.818744000	2.256863000	2.007525000
1	-2.912081000	3.858740000	-9.400073000
1	-3.173942000	5.530277000	-8.952662000
1	-2.353314000	5.471546000	-11.212887000
1	-1.139806000	6.210235000	-10.192103000
1	-1.090785000	3.303787000	-11.104519000
1	0.161639000	4.099890000	-10.177120000
1	0.525633000	5.708820000	-12.049324000
1	-0.705413000	4.889235000	-12.984779000
1	1.876944000	3.621693000	-11.977817000
1	0.633750000	2.783976000	-12.881173000
1	2.152942000	5.227170000	-13.883067000
1	0.980646000	4.313546000	-14.806026000
1	3.633651000	3.233945000	-13.764570000
1	3.339600000	3.789711000	-15.394884000
1	1.611266000	1.981942000	-15.690979000
1	1.995154000	1.374320000	-14.083578000
1	3.216315000	1.340723000	-15.347756000
7	2.569231000	2.832868000	4.222366000

6	2.305236000	3.955370000	3.256314000
6	0.856216000	4.187260000	2.899996000
6	0.763538000	5.235044000	1.802322000
6	-0.664893000	5.660788000	1.521554000
6	-0.775256000	6.639174000	0.366716000
6	-2.171139000	7.217480000	0.214534000
6	-2.321548000	8.195792000	-0.940832000
6	-2.329869000	7.553077000	-2.317537000
6	2.234062000	1.520334000	3.608927000
6	1.792950000	3.011628000	5.475959000
6	4.019678000	2.846573000	4.550876000
6	-2.502679000	8.565767000	-3.435126000
6	-2.680576000	7.938860000	-4.805269000
6	-2.859193000	8.964124000	-5.909483000
6	-3.128618000	8.349205000	-7.270405000
6	-3.307066000	9.387496000	-8.364404000
6	-3.573312000	8.805595000	-9.744219000
6	-4.922548000	8.122069000	-9.886260000
6	-5.167876000	7.600059000	-11.289523000
1	2.889108000	3.720170000	2.369015000
1	2.727084000	4.847740000	3.714902000
1	0.383665000	3.267934000	2.555311000
1	0.305470000	4.535581000	3.774038000
1	1.220038000	4.841881000	0.890706000
1	1.348019000	6.114827000	2.085624000
1	-1.276688000	4.778521000	1.311355000
1	-1.083174000	6.116043000	2.424125000
1	-0.068246000	7.460526000	0.520062000
1	-0.470206000	6.140516000	-0.556393000
1	-2.892537000	6.402870000	0.096025000
1	-2.436486000	7.723732000	1.146400000
1	-1.519949000	8.939683000	-0.889897000
1	-3.256020000	8.749604000	-0.817146000
1	-3.145891000	6.825402000	-2.362747000
1	-1.408024000	6.988140000	-2.481303000
1	2.815721000	1.407007000	2.700282000
1	1.176345000	1.486670000	3.379355000
1	2.486633000	0.740046000	4.320216000
1	1.998193000	4.001207000	5.873053000
1	2.109314000	2.249607000	6.181070000
1	0.736747000	2.899536000	5.261762000
1	4.583014000	2.738680000	3.629484000
1	4.258616000	3.789705000	5.031528000
1	4.229333000	2.018375000	5.219923000
1	-3.372972000	9.192836000	-3.217164000
1	-1.640029000	9.239297000	-3.451146000
1	-1.819045000	7.304619000	-5.034522000
1	-3.550261000	7.274882000	-4.785423000
1	-1.966219000	9.594281000	-5.965209000
1	-3.686565000	9.631691000	-5.648970000
1	-4.019078000	7.719474000	-7.202133000

1	-2.304302000	7.681668000	-7.539852000
1	-4.127134000	10.060234000	-8.093554000
1	-2.407808000	10.007410000	-8.407737000
1	-2.780282000	8.094802000	-9.996125000
1	-3.510371000	9.606430000	-10.486360000
1	-5.710099000	8.831179000	-9.615714000
1	-4.998941000	7.295530000	-9.177205000
1	-4.414182000	6.862137000	-11.567995000
1	-5.125798000	8.408382000	-12.021274000
1	-6.145100000	7.124511000	-11.374180000
16	0.349885000	0.345632000	0.596492000
8	1.336726000	1.587130000	0.266642000
8	-0.593496000	0.955153000	1.531075000
8	-0.243900000	-0.049873000	-0.681559000
8	1.189785000	-0.696645000	1.190385000
6	3.182206000	4.952873000	-1.701283000
6	2.464322000	6.010727000	-2.525645000
6	2.453678000	3.622732000	-1.619579000
6	2.440828000	5.730428000	-4.018003000
6	3.213726000	2.597325000	-0.799124000
6	1.757749000	6.824212000	-4.819171000
6	2.461624000	1.310719000	-0.576730000
6	1.784814000	6.565796000	-6.315922000
1	4.184803000	4.794737000	-2.111218000
1	3.325472000	5.328901000	-0.684596000
1	1.438790000	6.118692000	-2.158530000
1	2.949117000	6.976738000	-2.361295000
1	2.284940000	3.217439000	-2.619732000
1	1.465910000	3.784260000	-1.182744000
1	1.936461000	4.780970000	-4.215137000
1	3.469082000	5.610428000	-4.373827000
1	3.488234000	3.014577000	0.172458000
1	4.149475000	2.344001000	-1.302287000
1	0.717083000	6.914131000	-4.489990000
1	2.232867000	7.782410000	-4.594202000
1	2.104202000	0.897345000	-1.521644000
1	3.092796000	0.570784000	-0.084882000
1	1.364033000	5.574722000	-6.504469000
1	2.823820000	6.523153000	-6.657822000
6	1.022718000	7.589588000	-7.144781000
1	0.887545000	7.192746000	-8.152615000
1	0.017521000	7.711021000	-6.728675000
6	1.694794000	8.949765000	-7.230384000
1	2.661752000	8.837967000	-7.731636000
1	1.912403000	9.315524000	-6.224725000
6	0.869544000	10.001507000	-7.958099000
1	-0.066599000	10.157534000	-7.415247000
1	1.404112000	10.953446000	-7.920871000
6	0.560624000	9.663737000	-9.405941000
1	-0.074473000	8.781823000	-9.491422000
1	1.477376000	9.468426000	-9.965746000

1	0.040923000	10.487464000	-9.896006000
**3- CTA⁺/(C_{12}-OSO_3)⁻ 2:1 ΔG=1.64**			
7	0.165796000	-4.154016000	0.892032000
6	1.515497000	-3.507637000	1.048057000
6	2.549424000	-3.891860000	0.013780000
6	3.927114000	-3.460855000	0.487876000
6	5.014347000	-3.691076000	-0.544367000
6	6.399730000	-3.411872000	0.007069000
6	7.509352000	-3.534071000	-1.020694000
6	8.885260000	-3.351583000	-0.408672000
6	10.027503000	-3.396806000	-1.406445000
6	-0.374834000	-3.944164000	-0.475973000
6	-0.753969000	-3.517201000	1.873859000
6	0.239242000	-5.608915000	1.182621000
6	11.382490000	-3.269098000	-0.734508000
6	12.557185000	-3.290229000	-1.695675000
6	13.909977000	-3.319290000	-0.998991000
6	14.274571000	-2.041822000	-0.261049000
6	15.564260000	-2.177109000	0.531282000
6	16.034582000	-0.884154000	1.181211000
6	16.702135000	0.083028000	0.218291000
6	17.084093000	1.393556000	0.879239000
1	1.850106000	-3.776829000	2.048724000
1	1.335457000	-2.435068000	1.024173000
1	2.558704000	-4.968013000	-0.158881000
1	2.322506000	-3.412796000	-0.939466000
1	4.172915000	-4.007428000	1.402424000
1	3.907110000	-2.402802000	0.758945000
1	4.968256000	-4.724433000	-0.900721000
1	4.828035000	-3.056540000	-1.415840000
1	6.421493000	-2.407833000	0.441464000
1	6.597563000	-4.101669000	0.833220000
1	7.453198000	-4.513752000	-1.505321000
1	7.356676000	-2.792400000	-1.810962000
1	9.041067000	-4.125051000	0.349528000
1	8.916737000	-2.397068000	0.124621000
1	9.903206000	-2.593438000	-2.139621000
1	9.986422000	-4.334339000	-1.969767000
1	-0.373526000	-2.880595000	-0.695274000
1	-1.386156000	-4.337944000	-0.501309000
1	0.244482000	-4.478412000	-1.187656000
1	-0.345578000	-3.660673000	2.869534000
1	-1.726035000	-3.993147000	1.793689000
1	-0.823313000	-2.459605000	1.640528000
1	-0.763916000	-6.018607000	1.122498000
1	0.876352000	-6.092546000	0.451976000
1	0.640043000	-5.741457000	2.182571000
1	11.399174000	-2.347821000	-0.148908000
1	11.499060000	-4.086087000	-0.015389000
1	12.471882000	-4.171469000	-2.337407000
1	12.503886000	-2.422014000	-2.360616000

1	14.691169000	-3.529757000	-1.734353000
1	13.923857000	-4.157066000	-0.294053000
1	13.473998000	-1.761468000	0.428590000
1	14.355769000	-1.224649000	-0.983310000
1	15.413420000	-2.937091000	1.302416000
1	16.356130000	-2.560347000	-0.120313000
1	16.741278000	-1.115605000	1.982488000
1	15.183332000	-0.390908000	1.661889000
1	16.042122000	0.280675000	-0.628056000
1	17.595088000	-0.393017000	-0.196052000
1	17.596883000	2.059558000	0.185459000
1	17.747319000	1.223522000	1.729229000
1	16.200191000	1.915409000	1.249939000
7	0.866532000	4.841431000	0.867949000
6	1.177615000	3.854065000	1.961269000
6	2.561939000	3.957530000	2.559751000
6	2.771650000	2.893638000	3.631268000
6	2.811329000	1.473647000	3.096140000
6	3.171419000	0.441326000	4.148438000
6	3.135195000	-0.971914000	3.594447000
6	3.528474000	-2.055108000	4.587697000
6	4.960523000	-1.978882000	5.100507000
6	1.053786000	6.240256000	1.329914000
6	-0.559204000	4.652767000	0.482679000
6	1.715376000	4.588866000	-0.326541000
6	6.017540000	-2.103207000	4.017153000
6	7.431896000	-2.133272000	4.567554000
6	8.490585000	-2.294202000	3.492689000
6	9.905459000	-2.358775000	4.039591000
6	10.945199000	-2.566124000	2.951675000
6	12.367129000	-2.747877000	3.458629000
6	12.991549000	-1.493629000	4.044944000
6	14.392749000	-1.734474000	4.574325000
1	1.009396000	2.876984000	1.515930000
1	0.424377000	4.016053000	2.730576000
1	3.326766000	3.839578000	1.791537000
1	2.700360000	4.938719000	3.011326000
1	1.990847000	2.981397000	4.391811000
1	3.715657000	3.109158000	4.135837000
1	1.848019000	1.207067000	2.655966000
1	3.543617000	1.420902000	2.286250000
1	4.165645000	0.668249000	4.541202000
1	2.480978000	0.519056000	4.994169000
1	2.124827000	-1.179632000	3.232075000
1	3.782329000	-1.025529000	2.715864000
1	3.378870000	-3.030797000	4.116198000
1	2.844164000	-2.020439000	5.440056000
1	5.108959000	-2.781156000	5.828466000
1	5.113883000	-1.046338000	5.649678000
1	2.106462000	6.422032000	1.510468000
1	0.480227000	6.383943000	2.240556000

1	0.694391000	6.904745000	0.550725000
1	-0.788884000	5.339844000	-0.325514000
1	-1.180443000	4.869045000	1.346027000
1	-0.699316000	3.625666000	0.159667000
1	1.401141000	5.266682000	-1.113908000
1	2.753003000	4.774186000	-0.075073000
1	1.576600000	3.557154000	-0.633460000
1	5.834518000	-3.016037000	3.441165000
1	5.930106000	-1.274630000	3.309145000
1	7.620790000	-1.214391000	5.131346000
1	7.521360000	-2.954662000	5.285297000
1	8.414896000	-1.467895000	2.778338000
1	8.285604000	-3.205126000	2.922237000
1	9.974025000	-3.179628000	4.760664000
1	10.116968000	-1.443029000	4.597526000
1	10.664082000	-3.445848000	2.368286000
1	10.914361000	-1.721888000	2.255688000
1	12.998591000	-3.095662000	2.635566000
1	12.381911000	-3.543738000	4.210326000
1	12.364346000	-1.105697000	4.850243000
1	13.016718000	-0.717686000	3.274246000
1	14.382385000	-2.464369000	5.385361000
1	14.839458000	-0.816834000	4.957695000
1	15.046636000	-2.120637000	3.791263000
16	-0.091156000	0.303600000	-0.470710000
8	1.340728000	1.048885000	-0.612762000
8	-0.115546000	-0.276665000	0.873570000
8	-1.023088000	1.410965000	-0.662680000
8	-0.127347000	-0.704897000	-1.531511000
6	6.241427000	1.207033000	-0.593557000
6	7.534074000	0.453843000	-0.342889000
6	5.004748000	0.351236000	-0.396251000
6	8.772596000	1.301913000	-0.564229000
6	3.717368000	1.108530000	-0.661383000
6	10.065092000	0.535613000	-0.342328000
6	2.508531000	0.236580000	-0.445692000
6	11.327904000	1.359630000	-0.548249000
1	6.190011000	2.074767000	0.071270000
1	6.247036000	1.603203000	-1.613461000
1	7.536072000	0.064292000	0.680007000
1	7.574501000	-0.419290000	-1.000551000
1	4.992352000	-0.036437000	0.626728000
1	5.059035000	-0.521197000	-1.053438000
1	8.746712000	2.165628000	0.107819000
1	8.744371000	1.704212000	-1.579530000
1	3.709751000	1.489959000	-1.684714000
1	3.653539000	1.973443000	0.002199000
1	10.062653000	0.132909000	0.674229000
1	10.090079000	-0.330622000	-1.010320000
1	2.481277000	-0.582609000	-1.165699000
1	2.505533000	-0.182548000	0.560505000

1	12.181841000	0.793948000	-0.169161000
1	11.269652000	2.268768000	0.059570000
6	11.578576000	1.741935000	-1.998317000
1	11.720225000	0.829516000	-2.587067000
1	10.690719000	2.230269000	-2.403822000
6	12.768576000	2.665162000	-2.208841000
1	12.620237000	3.582923000	-1.630693000
1	12.796451000	2.969533000	-3.258899000
6	14.112400000	2.058565000	-1.845137000
1	14.135360000	1.818030000	-0.780446000
1	14.230529000	1.108954000	-2.375266000
6	15.273437000	2.976097000	-2.178870000
1	16.229900000	2.520216000	-1.924525000
1	15.197688000	3.917042000	-1.631307000
1	15.289059000	3.214190000	-3.243672000
**4- $CTA^+/(C_{12}\text{-}OSO_3)^-$ 2:1 ΔG=3.58**			
7	4.779361000	1.047266000	2.504432000
6	3.584591000	1.617240000	3.223540000
6	3.495992000	3.123682000	3.247329000
6	2.267060000	3.568139000	4.025565000
6	2.179012000	5.082405000	4.096187000
6	1.020839000	5.623233000	4.918605000
6	-0.358634000	5.359205000	4.337298000
6	-1.437143000	6.179816000	5.023029000
6	-2.832454000	5.952858000	4.469714000
6	4.665874000	-0.436333000	2.551221000
6	6.046222000	1.450073000	3.165937000
6	4.801572000	1.474608000	1.080421000
6	-3.838706000	6.983080000	4.951036000
6	-5.245361000	6.744283000	4.434404000
6	-6.210073000	7.871053000	4.757650000
6	-7.638683000	7.556555000	4.347390000
6	-8.575938000	8.756027000	4.368543000
6	-8.701236000	9.470778000	5.706021000
6	-9.250525000	8.610313000	6.830408000
6	-9.480428000	9.403240000	8.103081000
1	3.637014000	1.220656000	4.236034000
1	2.715640000	1.189192000	2.729444000
1	4.380782000	3.555807000	3.714980000
1	3.433655000	3.520969000	2.234305000
1	2.309629000	3.159181000	5.038264000
1	1.370286000	3.154218000	3.558276000
1	3.114167000	5.462025000	4.516029000
1	2.115457000	5.485536000	3.080870000
1	1.071745000	5.219426000	5.934444000
1	1.150275000	6.704438000	5.016546000
1	-0.351463000	5.605605000	3.270685000
1	-0.604558000	4.296223000	4.405169000
1	-1.182435000	7.239482000	4.922149000
1	-1.432054000	5.968459000	6.097178000
1	-3.174225000	4.948644000	4.737560000

1	-2.795429000	5.979896000	3.376101000
1	5.522588000	-0.863215000	2.039283000
1	3.743759000	-0.722282000	2.056625000
1	4.655873000	-0.748979000	3.590741000
1	6.864786000	0.933962000	2.674385000
1	5.995756000	1.165135000	4.212252000
1	6.178629000	2.521006000	3.069915000
1	5.617276000	0.955634000	0.586634000
1	3.851496000	1.208176000	0.629425000
1	4.965257000	2.544355000	1.030049000
1	-3.852418000	7.000163000	6.045072000
1	-3.503413000	7.976300000	4.636172000
1	-5.213546000	6.603949000	3.349403000
1	-5.630311000	5.808632000	4.848825000
1	-5.877853000	8.782841000	4.251004000
1	-6.169579000	8.085054000	5.828999000
1	-8.028582000	6.760208000	4.987685000
1	-7.632211000	7.147670000	3.333125000
1	-8.234487000	9.475890000	3.619484000
1	-9.570340000	8.432406000	4.048244000
1	-9.357884000	10.335289000	5.574429000
1	-7.730576000	9.875321000	6.005706000
1	-8.563664000	7.785085000	7.033323000
1	-10.190354000	8.153427000	6.506981000
1	-10.185099000	10.218584000	7.931411000
1	-8.548312000	9.842435000	8.462543000
1	-9.881146000	8.778157000	8.900643000
7	0.007682000	-3.635613000	0.886991000
6	0.271242000	-3.052758000	2.249637000
6	1.588312000	-3.448433000	2.881085000
6	1.596261000	-3.125291000	4.369946000
6	1.397240000	-1.656535000	4.699328000
6	1.414751000	-1.392416000	6.194862000
6	1.239710000	0.070548000	6.579489000
6	-0.015874000	0.695521000	5.992583000
6	-0.419150000	2.035362000	6.586713000
6	1.148298000	-3.386771000	-0.033332000
6	-1.205649000	-2.968959000	0.340316000
6	-0.251333000	-5.095537000	0.975812000
6	-0.861241000	1.968298000	8.040387000
6	-1.566260000	3.227608000	8.525520000
6	-2.954311000	3.412009000	7.937567000
6	-3.696976000	4.617745000	8.483271000
6	-5.082252000	4.751332000	7.876579000
6	-5.880195000	5.955081000	8.348646000
6	-6.335297000	5.872811000	9.795524000
6	-7.163688000	7.072494000	10.213982000
1	-0.566199000	-3.372361000	2.867975000
1	0.212952000	-1.975451000	2.123788000
1	1.769264000	-4.515939000	2.761295000
1	2.409038000	-2.925329000	2.389214000

1	2.547428000	-3.463151000	4.786681000
1	0.817537000	-3.712961000	4.864239000
1	2.174380000	-1.062242000	4.211286000
1	0.446581000	-1.315313000	4.287923000
1	0.615717000	-1.976561000	6.662128000
1	2.352236000	-1.766814000	6.614884000
1	1.217388000	0.138660000	7.668681000
1	2.111753000	0.645844000	6.253557000
1	-0.851914000	-0.002777000	6.110059000
1	0.124028000	0.820615000	4.917638000
1	-1.233272000	2.437502000	5.980341000
1	0.405651000	2.747688000	6.494093000
1	0.859342000	-3.720064000	-1.025267000
1	2.007752000	-3.952321000	0.308377000
1	1.366401000	-2.323688000	-0.030647000
1	-2.025430000	-3.120073000	1.036052000
1	-1.441022000	-3.416041000	-0.620293000
1	-0.995933000	-1.910792000	0.222751000
1	-1.098852000	-5.256618000	1.634410000
1	-0.473742000	-5.462659000	-0.021094000
1	0.628148000	-5.596079000	1.362764000
1	-1.534166000	1.114615000	8.172858000
1	0.003712000	1.775034000	8.677508000
1	-1.651371000	3.191900000	9.614855000
1	-0.951073000	4.103902000	8.296279000
1	-3.544187000	2.511414000	8.136807000
1	-2.894851000	3.500263000	6.849857000
1	-3.121617000	5.526228000	8.276974000
1	-3.764383000	4.536370000	9.571072000
1	-4.977446000	4.796047000	6.790361000
1	-5.653540000	3.841961000	8.084943000
1	-6.763918000	6.065127000	7.712715000
1	-5.288560000	6.865143000	8.205609000
1	-5.469112000	5.781962000	10.454267000
1	-6.920484000	4.958239000	9.930993000
1	-7.487718000	6.994622000	11.252038000
1	-8.054751000	7.164878000	9.592200000
1	-6.592603000	7.996874000	10.111289000
16	0.699167000	0.783336000	0.449445000
8	-0.300976000	0.597635000	1.710825000
8	1.767528000	-0.156449000	0.781948000
8	-0.072826000	0.409058000	-0.736512000
8	1.110712000	2.186891000	0.457128000
6	-4.467300000	1.485146000	4.148220000
6	-5.624308000	2.442310000	4.362162000
6	-3.548977000	1.911554000	3.018180000
6	-6.517203000	2.063267000	5.528389000
6	-2.367413000	0.978070000	2.834478000
6	-7.628865000	3.065266000	5.779659000
6	-1.430455000	1.477447000	1.766637000
6	-8.475508000	2.733421000	6.993869000

1	-3.885451000	1.411506000	5.072460000
1	-4.852317000	0.480891000	3.947570000
1	-5.226560000	3.446885000	4.530860000
1	-6.222785000	2.501645000	3.447865000
1	-3.180911000	2.921538000	3.219768000
1	-4.119200000	1.972139000	2.086874000
1	-5.905485000	1.973746000	6.431213000
1	-6.949330000	1.073211000	5.353015000
1	-2.714592000	-0.023584000	2.571477000
1	-1.820707000	0.892400000	3.774308000
1	-7.191572000	4.059334000	5.909778000
1	-8.270111000	3.128533000	4.895001000
1	-1.922942000	1.492602000	0.793448000
1	-1.078199000	2.483899000	1.997176000
1	-7.824196000	2.621506000	7.866284000
1	-8.957450000	1.761781000	6.847524000
6	-9.531609000	3.780962000	7.295103000
1	-9.038626000	4.745449000	7.443291000
1	-10.184200000	3.900386000	6.424206000
6	-10.377142000	3.435813000	8.508378000
1	-9.728119000	3.343666000	9.385313000
1	-10.823229000	2.449440000	8.354465000
6	-11.483617000	4.435667000	8.809213000
1	-12.124644000	4.536200000	7.928805000
1	-12.117112000	4.031824000	9.602146000
6	-10.977585000	5.804394000	9.228621000
1	-11.803827000	6.470240000	9.478993000
1	-10.400244000	6.281015000	8.437419000
1	-10.333496000	5.727335000	10.106496000

5- $CTA^+/(C_{12}\text{-}OSO_3)^-$ 2:1 ΔG=4.03

7	1.442815000	2.351221000	2.929639000
6	1.570906000	2.754477000	1.485067000
6	0.648403000	3.858017000	1.023902000
6	1.003711000	4.242396000	-0.402789000
6	0.076043000	5.289922000	-0.988094000
6	0.432724000	5.634240000	-2.423129000
6	-0.564072000	6.550964000	-3.115676000
6	-0.767728000	7.908974000	-2.458674000
6	0.511413000	8.709986000	-2.293002000
6	2.292614000	1.146771000	3.133820000
6	0.037309000	2.007574000	3.270142000
6	1.923396000	3.432043000	3.827506000
6	0.265824000	10.154495000	-1.897387000
6	1.544522000	10.953550000	-1.729080000
6	1.310060000	12.418156000	-1.412622000
6	2.598053000	13.209232000	-1.277968000
6	2.375511000	14.682367000	-0.990032000
6	3.672282000	15.462088000	-0.860302000
6	3.491472000	16.928573000	-0.498222000
6	2.791307000	17.748166000	-1.567614000

1	1.394236000	1.849458000	0.908746000
1	2.613048000	3.042376000	1.355748000
1	-0.388936000	3.523196000	1.056209000
1	0.730101000	4.737356000	1.663353000
1	0.983440000	3.348635000	-1.032922000
1	2.030502000	4.614200000	-0.430185000
1	-0.955498000	4.928257000	-0.944228000
1	0.109948000	6.188246000	-0.366691000
1	1.430857000	6.078816000	-2.448336000
1	0.504077000	4.709054000	-3.000681000
1	-0.232788000	6.708182000	-4.146125000
1	-1.528627000	6.038740000	-3.178679000
1	-1.247670000	7.787560000	-1.483839000
1	-1.469537000	8.483867000	-3.069107000
1	1.074324000	8.686183000	-3.231412000
1	1.152476000	8.236697000	-1.545128000
1	2.238926000	0.859396000	4.179110000
1	3.314076000	1.395818000	2.863120000
1	1.918023000	0.350093000	2.499494000
1	0.024352000	1.602889000	4.277316000
1	-0.322754000	1.274246000	2.555126000
1	-0.568499000	2.905698000	3.228607000
1	1.865032000	3.076471000	4.851353000
1	2.951405000	3.664707000	3.568374000
1	1.295346000	4.306600000	3.706107000
1	-0.360355000	10.632582000	-2.657492000
1	-0.309382000	10.185792000	-0.966623000
1	2.151662000	10.503640000	-0.937957000
1	2.138323000	10.877537000	-2.645106000
1	0.731730000	12.504690000	-0.487557000
1	0.694387000	12.863253000	-2.200419000
1	3.179712000	13.105889000	-2.199651000
1	3.209040000	12.772038000	-0.482211000
1	1.759905000	15.109507000	-1.785490000
1	1.798498000	14.787549000	-0.065697000
1	4.230998000	15.389478000	-1.798870000
1	4.293359000	14.983504000	-0.098161000
1	4.473419000	17.365165000	-0.302364000
1	2.934099000	16.999701000	0.440000000
1	3.321535000	17.678373000	-2.519072000
1	2.747251000	18.800932000	-1.288109000
1	1.769042000	17.409283000	-1.732850000
7	-3.027176000	1.876413000	-2.254395000
6	-1.535190000	1.942526000	-2.432681000
6	-0.990962000	1.382298000	-3.725397000
6	0.528707000	1.413751000	-3.663133000
6	1.214550000	0.942453000	-4.933585000
6	0.934553000	1.814263000	-6.147077000
6	1.883109000	1.532654000	-7.298633000
6	1.559711000	2.290727000	-8.579247000
6	1.458256000	3.802986000	-8.431119000

6	-3.496728000	0.466721000	-2.215840000
6	-3.729946000	2.611250000	-3.335661000
6	-3.345744000	2.516230000	-0.948777000
6	2.693406000	4.465264000	-7.848352000
6	2.576549000	5.977761000	-7.780261000
6	3.738383000	6.621803000	-7.045590000
6	3.718295000	8.142769000	-7.020962000
6	2.467943000	8.753203000	-6.409998000
6	2.584332000	10.252684000	-6.202652000
6	1.298641000	10.897239000	-5.717176000
6	1.449720000	12.379402000	-5.432508000
1	-1.119701000	1.413244000	-1.578733000
1	-1.278227000	2.996116000	-2.346027000
1	-1.317441000	0.353405000	-3.878707000
1	-1.343644000	1.970219000	-4.571504000
1	0.855763000	0.798942000	-2.822530000
1	0.860040000	2.432575000	-3.444589000
1	2.292412000	0.929830000	-4.751446000
1	0.929375000	-0.091451000	-5.149186000
1	-0.095255000	1.671343000	-6.485003000
1	1.016464000	2.863834000	-5.851268000
1	2.903159000	1.761286000	-6.978907000
1	1.871365000	0.461272000	-7.517058000
1	0.614436000	1.917241000	-8.982593000
1	2.323170000	2.056339000	-9.326167000
1	0.595668000	4.055185000	-7.808630000
1	1.252964000	4.234939000	-9.414449000
1	-3.345702000	0.011529000	-3.187471000
1	-4.554740000	0.470185000	-1.973588000
1	-2.929744000	-0.063120000	-1.457626000
1	-3.343481000	3.625472000	-3.370781000
1	-4.791070000	2.620350000	-3.107570000
1	-3.560032000	2.107349000	-4.279982000
1	-3.035867000	3.555490000	-0.989631000
1	-4.416328000	2.449723000	-0.783939000
1	-2.805165000	1.988315000	-0.169656000
1	2.876049000	4.082940000	-6.840435000
1	3.572032000	4.191863000	-8.441205000
1	2.507242000	6.386695000	-8.793314000
1	1.638405000	6.235271000	-7.281988000
1	4.674893000	6.290096000	-7.502903000
1	3.754501000	6.246417000	-6.018769000
1	3.838363000	8.524593000	-8.039550000
1	4.591005000	8.492869000	-6.461920000
1	1.605575000	8.547922000	-7.049013000
1	2.257583000	8.271777000	-5.449679000
1	2.891631000	10.726919000	-7.140132000
1	3.383707000	10.455276000	-5.482709000
1	0.960060000	10.385926000	-4.813379000
1	0.516166000	10.744024000	-6.465203000
1	0.512021000	12.819154000	-5.092120000

1	1.766362000	12.920064000	-6.326112000
1	2.198358000	12.553565000	-4.657770000
16	-0.374410000	-0.949754000	0.182485000
8	1.144441000	-0.402873000	0.035816000
8	-0.743441000	-1.478355000	-1.131047000
8	-0.338104000	-1.970147000	1.231018000
8	-1.092305000	0.266542000	0.557632000
6	4.970350000	0.899566000	-1.720478000
6	5.043544000	2.101399000	-2.652896000
6	3.550461000	0.424069000	-1.464280000
6	4.762852000	3.424453000	-1.961017000
6	3.477176000	-0.677195000	-0.423143000
6	4.654967000	4.593386000	-2.923496000
6	2.138124000	-1.363273000	-0.345512000
6	4.625743000	5.946039000	-2.234439000
1	5.541642000	0.069156000	-2.142557000
1	5.453891000	1.149274000	-0.770703000
1	6.034715000	2.153228000	-3.109932000
1	4.336376000	1.960461000	-3.476836000
1	3.130453000	0.057641000	-2.405060000
1	2.922921000	1.261615000	-1.153619000
1	5.563765000	3.619699000	-1.241282000
1	3.841100000	3.353332000	-1.378580000
1	3.744470000	-0.285196000	0.560763000
1	4.208835000	-1.454214000	-0.658272000
1	5.499583000	4.568949000	-3.619473000
1	3.754150000	4.474190000	-3.534885000
1	1.863459000	-1.783273000	-1.314334000
1	2.154785000	-2.163728000	0.394192000
1	5.553212000	6.073748000	-1.670984000
1	3.815440000	5.965845000	-1.499000000
6	4.438966000	7.096182000	-3.207673000
1	5.228807000	7.062851000	-3.965022000
1	3.499429000	6.942730000	-3.744294000
6	4.415720000	8.474401000	-2.567756000
1	3.655094000	8.494926000	-1.782116000
1	4.095144000	9.205420000	-3.315743000
6	5.744700000	8.929852000	-1.990556000
1	6.069364000	8.240416000	-1.208561000
1	6.507645000	8.886321000	-2.773070000
6	5.676749000	10.335859000	-1.423013000
1	6.638007000	10.652466000	-1.017895000
1	4.939604000	10.397251000	-0.620820000
1	5.384918000	11.053731000	-2.191554000

1-CTA$^+$/B(Ph)$_4^-$ 1:1 ΔG=0.0

7	1.589557000	-3.513942000	4.114465000
6	2.315984000	-2.822865000	2.989532000
6	3.501216000	-3.572113000	2.424795000
6	4.315108000	-2.647097000	1.534556000
6	5.414906000	-3.378621000	0.788356000

6	6.377602000	-2.452811000	0.056771000
6	5.742905000	-1.521428000	-0.967011000
6	5.036190000	-2.231940000	-2.107433000
6	4.454001000	-1.266990000	-3.126847000
6	2.417385000	-3.529666000	5.348122000
6	1.226895000	-4.905536000	3.743806000
6	0.344536000	-2.749328000	4.392992000
6	3.888075000	-1.932917000	-4.372417000
6	2.756765000	-2.907485000	-4.097907000
6	2.076380000	-3.415203000	-5.354976000
6	0.912275000	-4.342468000	-5.058319000
6	0.215300000	-4.860761000	-6.301958000
6	-0.956265000	-5.774876000	-5.994896000
6	-1.655353000	-6.298172000	-7.235877000
6	-2.826881000	-7.207038000	-6.915392000
1	2.617207000	-1.856087000	3.388381000
1	1.573676000	-2.648016000	2.213117000
1	4.144405000	-3.956420000	3.217034000
1	3.159716000	-4.427061000	1.840297000
1	4.750383000	-1.852987000	2.146846000
1	3.646685000	-2.153816000	0.827474000
1	5.988045000	-3.981631000	1.497687000
1	4.964570000	-4.085242000	0.086728000
1	6.908139000	-1.847675000	0.797084000
1	7.137812000	-3.060326000	-0.441849000
1	5.038628000	-0.847073000	-0.471959000
1	6.526334000	-0.881352000	-1.381785000
1	4.240589000	-2.862356000	-1.706082000
1	5.741728000	-2.905144000	-2.606007000
1	5.231466000	-0.559142000	-3.427518000
1	3.668455000	-0.672503000	-2.648334000
1	3.319073000	-4.103158000	5.170920000
1	1.837301000	-3.991867000	6.140523000
1	2.664880000	-2.506134000	5.611233000
1	0.673017000	-4.880412000	2.811474000
1	0.615588000	-5.320910000	4.538691000
1	2.131716000	-5.491619000	3.630178000
1	-0.175387000	-3.225379000	5.218441000
1	0.613727000	-1.731305000	4.654917000
1	-0.273726000	-2.753727000	3.501248000
1	3.524821000	-1.155393000	-5.050212000
1	4.692791000	-2.450763000	-4.904073000
1	3.129705000	-3.761114000	-3.526431000
1	2.012130000	-2.419419000	-3.465193000
1	2.805981000	-3.934639000	-5.984179000
1	1.718211000	-2.563100000	-5.941341000
1	0.186671000	-3.814451000	-4.431321000
1	1.267940000	-5.189854000	-4.463886000
1	-0.135028000	-4.013632000	-6.899694000
1	0.937976000	-5.396327000	-6.925196000
1	-1.679566000	-5.237945000	-5.373283000

1	-0.607143000	-6.620747000	-5.394419000
1	-0.932712000	-6.836527000	-7.854971000
1	-2.001308000	-5.452457000	-7.836272000
1	-3.312327000	-7.568497000	-7.821925000
1	-3.577014000	-6.682005000	-6.322065000
1	-2.501036000	-8.076302000	-6.342124000
5	0.116913000	0.055732000	-0.150060000
6	1.118702000	0.492652000	1.081819000
6	0.661003000	0.562901000	2.400425000
1	-0.391449000	0.397021000	2.600813000
6	1.499509000	0.837402000	3.469416000
1	1.097356000	0.886545000	4.474367000
6	2.855000000	1.040037000	3.257567000
1	3.516771000	1.253085000	4.087405000
6	3.347337000	0.953864000	1.965794000
1	4.406001000	1.091722000	1.780701000
6	2.490188000	0.684283000	0.908425000
1	2.909371000	0.617788000	-0.088253000
6	0.084514000	-1.589690000	-0.081440000
6	-0.905722000	-2.315529000	0.581168000
1	-1.739989000	-1.788009000	1.027793000
6	-0.869690000	-3.699752000	0.688910000
1	-1.663754000	-4.220480000	1.210993000
6	0.179287000	-4.415794000	0.135473000
1	0.212696000	-5.494963000	0.215035000
6	1.193878000	-3.723302000	-0.507867000
1	2.034080000	-4.261591000	-0.930559000
6	1.138701000	-2.342794000	-0.604852000
1	1.948956000	-1.831438000	-1.110719000
6	0.645064000	0.591603000	-1.616394000
6	0.430515000	-0.123303000	-2.795601000
1	-0.015362000	-1.109191000	-2.741393000
6	0.772500000	0.373553000	-4.045207000
1	0.595906000	-0.226353000	-4.930053000
6	1.348217000	1.628180000	-4.162305000
1	1.624079000	2.020236000	-5.132931000
6	1.561605000	2.373956000	-3.012197000
1	2.002928000	3.361074000	-3.081532000
6	1.210184000	1.859963000	-1.773339000
1	1.382835000	2.470448000	-0.894459000
6	-1.380555000	0.727275000	-0.014515000
6	-2.483688000	0.172134000	-0.669448000
1	-2.362087000	-0.766117000	-1.198176000
6	-3.731237000	0.776091000	-0.677769000
1	-4.556419000	0.303740000	-1.197282000
6	-3.924586000	1.984669000	-0.024866000
1	-4.896059000	2.462142000	-0.024890000
6	-2.849144000	2.571912000	0.621743000
1	-2.975878000	3.520159000	1.130356000
6	-1.608174000	1.949505000	0.620129000
1	-0.787501000	2.438632000	1.130842000

2-CTA+/B(Ph)4− 1:1 ΔG=0.88			
7	3.083787000	1.232828000	5.152789000
6	2.612708000	-0.057943000	5.766702000
6	1.127018000	-0.307430000	5.676143000
6	0.789927000	-1.718925000	6.125679000
6	-0.680996000	-2.036592000	5.926774000
6	-1.007963000	-3.507053000	6.099572000
6	-2.466537000	-3.823305000	5.825937000
6	-2.765346000	-5.310183000	5.798515000
6	-4.209210000	-5.625004000	5.453828000
6	2.416071000	2.398010000	5.786502000
6	2.828107000	1.252683000	3.686008000
6	4.549367000	1.333539000	5.379999000
6	-4.468133000	-7.111626000	5.292772000
6	-5.901573000	-7.465126000	4.919745000
6	-6.418433000	-6.813715000	3.643265000
6	-5.564086000	-7.072598000	2.415611000
6	-6.151504000	-6.478426000	1.148512000
6	-5.238063000	-6.614522000	-0.055128000
6	-5.806034000	-5.992434000	-1.317220000
6	-4.872142000	-6.112124000	-2.506583000
1	2.942076000	-0.032902000	6.803845000
1	3.162723000	-0.841949000	5.250706000
1	0.576829000	0.409163000	6.286552000
1	0.786858000	-0.188096000	4.649815000
1	1.066435000	-1.857870000	7.174005000
1	1.387982000	-2.430229000	5.551252000
1	-1.284968000	-1.435848000	6.612786000
1	-0.973576000	-1.733949000	4.917060000
1	-0.383299000	-4.087230000	5.413108000
1	-0.740327000	-3.834338000	7.108673000
1	-3.095633000	-3.333870000	6.575518000
1	-2.746858000	-3.390551000	4.860910000
1	-2.509757000	-5.757526000	6.763952000
1	-2.113468000	-5.786757000	5.059379000
1	-4.465769000	-5.104656000	4.528204000
1	-4.870211000	-5.218266000	6.225755000
1	2.856747000	3.303828000	5.382057000
1	2.575067000	2.347774000	6.859427000
1	1.357570000	2.370097000	5.556833000
1	3.316511000	0.393136000	3.237635000
1	3.238343000	2.174955000	3.286943000
1	1.761294000	1.213425000	3.505501000
1	5.029276000	0.476001000	4.919050000
1	4.906865000	2.252748000	4.927294000
1	4.736414000	1.342006000	6.449020000
1	-3.784302000	-7.509546000	4.538565000
1	-4.213450000	-7.622216000	6.225494000
1	-5.976996000	-8.551257000	4.818151000
1	-6.563586000	-7.190314000	5.745567000
1	-6.510413000	-5.734169000	3.788662000

1	-7.432490000	-7.176713000	3.454840000
1	-5.428020000	-8.150808000	2.283912000
1	-4.564421000	-6.657133000	2.567860000
1	-7.113409000	-6.953787000	0.933795000
1	-6.367973000	-5.418471000	1.314902000
1	-5.028316000	-7.673345000	-0.236091000
1	-4.275440000	-6.146329000	0.172457000
1	-6.024123000	-4.937719000	-1.128411000
1	-6.763351000	-6.464975000	-1.552867000
1	-5.302446000	-5.659785000	-3.400075000
1	-4.658416000	-7.157902000	-2.732461000
1	-3.921172000	-5.615940000	-2.306468000
5	-0.259949000	-0.766504000	0.884598000
6	1.239895000	-1.318467000	1.287915000
6	2.366892000	-0.974388000	0.536327000
1	2.236614000	-0.396386000	-0.370931000
6	3.652235000	-1.345391000	0.899891000
1	4.493553000	-1.054354000	0.282346000
6	3.862684000	-2.090735000	2.050236000
1	4.862073000	-2.390896000	2.338328000
6	2.769288000	-2.438884000	2.826307000
1	2.912969000	-3.015963000	3.731762000
6	1.491452000	-2.049241000	2.449967000
1	0.661621000	-2.331279000	3.086451000
6	-0.341175000	0.728818000	1.572081000
6	0.278921000	1.829721000	0.972618000
1	0.744724000	1.705216000	0.001923000
6	0.325875000	3.079084000	1.569659000
1	0.819039000	3.901187000	1.064749000
6	-0.253622000	3.279189000	2.814405000
1	-0.219317000	4.251586000	3.288836000
6	-0.868876000	2.209252000	3.441665000
1	-1.317599000	2.339981000	4.419261000
6	-0.905146000	0.965027000	2.825777000
1	-1.386580000	0.148250000	3.348706000
6	-1.474473000	-1.768368000	1.372676000
6	-2.773081000	-1.294101000	1.576467000
1	-2.960231000	-0.228788000	1.512429000
6	-3.843920000	-2.132735000	1.847636000
1	-4.831918000	-1.714741000	1.999599000
6	-3.654155000	-3.503646000	1.921698000
1	-4.483622000	-4.164343000	2.135865000
6	-2.382366000	-4.011731000	1.712878000
1	-2.213586000	-5.081223000	1.760036000
6	-1.323793000	-3.155701000	1.442793000
1	-0.346068000	-3.589574000	1.272281000
6	-0.464206000	-0.698083000	-0.749270000
6	-1.281688000	0.254093000	-1.360848000
1	-1.722576000	1.036340000	-0.754178000
6	-1.555969000	0.240441000	-2.721088000
1	-2.195405000	1.002230000	-3.150903000

6	-1.014448000	-0.745152000	-3.531366000
1	-1.221476000	-0.761199000	-4.593726000
6	-0.204434000	-1.712324000	-2.955200000
1	0.224841000	-2.494590000	-3.569777000
6	0.056878000	-1.681309000	-1.593599000
1	0.689705000	-2.454526000	-1.173246000
**3-CTA$^+$/B(Ph)$_4^-$ 1:1 ΔG=1.13**			
7	-1.012942000	-4.299221000	3.124961000
6	-1.844602000	-3.054761000	3.285230000
6	-2.717519000	-3.006308000	4.518441000
6	-3.548405000	-1.728504000	4.530829000
6	-4.673667000	-1.704872000	3.508628000
6	-5.493556000	-0.423447000	3.541316000
6	-4.744762000	0.799544000	3.041334000
6	-5.576229000	2.068078000	3.066367000
6	-4.830143000	3.270871000	2.518769000
6	-0.118763000	-4.503070000	4.293340000
6	-0.174043000	-4.126469000	1.907150000
6	-1.875369000	-5.494369000	2.938816000
6	-5.633867000	4.557637000	2.595233000
6	-4.980887000	5.743578000	1.902363000
6	-3.651742000	6.167984000	2.500698000
6	-3.033590000	7.356096000	1.787760000
6	-1.688030000	7.763423000	2.357945000
6	-1.053921000	8.933287000	1.629402000
6	0.293552000	9.337788000	2.198151000
6	0.919398000	10.507700000	1.462924000
1	-2.437330000	-2.988495000	2.377567000
1	-1.140023000	-2.224622000	3.291494000
1	-3.385154000	-3.867269000	4.561961000
1	-2.097546000	-3.027810000	5.413739000
1	-2.884213000	-0.875584000	4.384156000
1	-3.980653000	-1.613103000	5.526951000
1	-4.274070000	-1.838736000	2.500325000
1	-5.329365000	-2.559369000	3.694687000
1	-6.389373000	-0.559256000	2.929894000
1	-5.845100000	-0.244274000	4.562299000
1	-3.843733000	0.959426000	3.637945000
1	-4.402920000	0.614595000	2.018262000
1	-5.892776000	2.273277000	4.093827000
1	-6.493207000	1.915917000	2.488743000
1	-4.552500000	3.078967000	1.477280000
1	-3.891718000	3.383657000	3.067102000
1	-0.716383000	-4.724290000	5.169730000
1	0.460293000	-3.597669000	4.446358000
1	0.537868000	-5.339080000	4.074948000
1	0.413106000	-5.028196000	1.764135000
1	0.475008000	-3.270849000	2.056235000
1	-0.826397000	-3.959132000	1.056007000
1	-1.234284000	-6.349180000	2.747721000
1	-2.455989000	-5.664463000	3.837455000

1	-2.529057000	-5.316704000	2.090513000
1	-6.615873000	4.385253000	2.146026000
1	-5.819334000	4.804767000	3.645324000
1	-4.834442000	5.501303000	0.845603000
1	-5.667603000	6.594151000	1.927937000
1	-2.946739000	5.334624000	2.465997000
1	-3.791290000	6.409280000	3.559424000
1	-3.720031000	8.207181000	1.833345000
1	-2.917658000	7.115568000	0.726173000
1	-1.805566000	8.016965000	3.416064000
1	-1.007779000	6.906678000	2.324045000
1	-1.731855000	9.791848000	1.662921000
1	-0.936364000	8.679120000	0.571264000
1	0.969675000	8.479304000	2.163405000
1	0.175813000	9.590337000	3.255378000
1	1.885743000	10.776995000	1.889313000
1	0.276788000	11.388107000	1.511219000
1	1.074627000	10.269206000	0.409621000
5	-0.226283000	0.483108000	0.032713000
6	0.090069000	0.265514000	1.633139000
6	1.122874000	-0.582638000	2.045054000
1	1.741973000	-1.058730000	1.292453000
6	1.392196000	-0.842421000	3.378754000
1	2.208711000	-1.502043000	3.647687000
6	0.612470000	-0.264912000	4.371184000
1	0.813041000	-0.465014000	5.415960000
6	-0.432585000	0.563296000	3.998957000
1	-1.060530000	1.014933000	4.757644000
6	-0.680688000	0.817502000	2.655982000
1	-1.505459000	1.471985000	2.401808000
6	-0.943128000	-0.915873000	-0.460401000
6	-2.233070000	-1.231799000	-0.021754000
1	-2.752284000	-0.530491000	0.622078000
6	-2.879557000	-2.404156000	-0.375037000
1	-3.881957000	-2.598648000	-0.012588000
6	-2.242311000	-3.332970000	-1.185994000
1	-2.739279000	-4.252482000	-1.467650000
6	-0.953445000	-3.067137000	-1.615829000
1	-0.431822000	-3.785804000	-2.236577000
6	-0.324471000	-1.881852000	-1.253574000
1	0.682746000	-1.707498000	-1.611218000
6	1.133019000	0.825900000	-0.834882000
6	2.336807000	1.251437000	-0.274473000
1	2.435546000	1.289549000	0.803149000
6	3.425485000	1.640040000	-1.046424000
1	4.339404000	1.964017000	-0.562952000
6	3.345409000	1.616856000	-2.428052000
1	4.190132000	1.915821000	-3.035334000
6	2.157723000	1.209416000	-3.020593000
1	2.070053000	1.191961000	-4.100329000
6	1.083772000	0.829994000	-2.233355000

1	0.164788000	0.530386000	-2.724829000
6	-1.186848000	1.796550000	-0.234900000
6	-0.832812000	3.037259000	0.306813000
1	0.019982000	3.091885000	0.974699000
6	-1.516255000	4.205269000	0.015115000
1	-1.201884000	5.141503000	0.459945000
6	-2.600835000	4.178607000	-0.851022000
1	-3.140175000	5.087350000	-1.084868000
6	-2.978992000	2.969169000	-1.406950000
1	-3.821072000	2.926622000	-2.087515000
6	-2.280843000	1.806810000	-1.098998000
1	-2.600161000	0.880157000	-1.559970000
**4-CTA$^+$/B(Ph)$_4^-$ 1:1 ΔG=1.77**			
7	0.971872000	-5.172095000	-0.953795000
6	1.602998000	-4.106466000	-1.809854000
6	2.261793000	-4.596124000	-3.079896000
6	2.991326000	-3.458788000	-3.784614000
6	2.087489000	-2.329585000	-4.244089000
6	2.828707000	-1.237917000	-4.991879000
6	1.918679000	-0.107477000	-5.436713000
6	2.652454000	0.985528000	-6.193773000
6	1.770506000	2.149317000	-6.621096000
6	1.991629000	-6.116911000	-0.430745000
6	-0.061640000	-5.922281000	-1.712380000
6	0.320510000	-4.491190000	0.198637000
6	0.741063000	1.788384000	-7.680344000
6	-0.080492000	2.968975000	-8.176989000
6	-0.978502000	3.580485000	-7.116762000
6	-1.871869000	4.688722000	-7.640978000
6	-2.744145000	5.302468000	-6.560786000
6	-3.638443000	6.416300000	-7.075350000
6	-4.577044000	6.999926000	-6.029804000
6	-3.868357000	7.717369000	-4.894526000
1	2.324575000	-3.601043000	-1.170162000
1	0.799667000	-3.409548000	-2.031952000
1	2.980652000	-5.383710000	-2.858304000
1	1.516946000	-5.015091000	-3.757157000
1	3.511353000	-3.877037000	-4.648982000
1	3.764801000	-3.061033000	-3.122803000
1	1.296332000	-2.736732000	-4.881629000
1	1.590204000	-1.878707000	-3.384446000
1	3.621230000	-0.837037000	-4.352240000
1	3.327902000	-1.667269000	-5.865977000
1	1.120526000	-0.522243000	-6.056949000
1	1.429071000	0.330915000	-4.560291000
1	3.133936000	0.553014000	-7.076622000
1	3.460054000	1.365054000	-5.561727000
1	2.403597000	2.950851000	-7.012664000
1	1.273004000	2.557921000	-5.738020000
1	2.425837000	-6.672359000	-1.253315000
1	1.500094000	-6.800441000	0.254338000

1	2.755326000	-5.546449000	0.088594000
1	-0.559204000	-6.601017000	-1.026959000
1	0.414519000	-6.484784000	-2.506688000
1	-0.772370000	-5.211257000	-2.121596000
1	-0.150797000	-5.244239000	0.822207000
1	1.082819000	-3.959208000	0.757513000
1	-0.420904000	-3.795837000	-0.181730000
1	1.260384000	1.332682000	-8.528315000
1	0.059331000	1.026181000	-7.294562000
1	0.589428000	3.738696000	-8.573320000
1	-0.698673000	2.640878000	-9.017293000
1	-0.371198000	3.975597000	-6.300333000
1	-1.600309000	2.793575000	-6.677896000
1	-2.505863000	4.298834000	-8.443352000
1	-1.253206000	5.470242000	-8.093273000
1	-3.365863000	4.520980000	-6.111851000
1	-2.101603000	5.677718000	-5.760334000
1	-4.232618000	6.032312000	-7.909122000
1	-3.016418000	7.217238000	-7.487689000
1	-5.201218000	6.199487000	-5.622658000
1	-5.258968000	7.698110000	-6.520406000
1	-3.225465000	8.511417000	-5.278682000
1	-3.245091000	7.038627000	-4.312955000
1	-4.585184000	8.172008000	-4.210419000
5	-0.060499000	0.496170000	-0.354604000
6	1.402281000	-0.243016000	-0.188195000
6	1.667060000	-1.041537000	0.928879000
1	0.909071000	-1.133209000	1.699141000
6	2.862103000	-1.721219000	1.097817000
1	3.021223000	-2.322251000	1.985275000
6	3.852973000	-1.637755000	0.129808000
1	4.789894000	-2.165886000	0.253044000
6	3.614704000	-0.877654000	-1.002281000
1	4.367145000	-0.813283000	-1.778917000
6	2.411573000	-0.199518000	-1.149946000
1	2.260636000	0.384245000	-2.049503000
6	-1.107873000	-0.716672000	-0.734008000
6	-1.979072000	-1.307429000	0.181327000
1	-2.027454000	-0.919976000	1.191606000
6	-2.798770000	-2.378965000	-0.151391000
1	-3.460438000	-2.803152000	0.594362000
6	-2.769007000	-2.909466000	-1.430039000
1	-3.403541000	-3.745349000	-1.695505000
6	-1.901407000	-2.357075000	-2.361797000
1	-1.853229000	-2.764341000	-3.364907000
6	-1.093851000	-1.287883000	-2.010165000
1	-0.424765000	-0.879613000	-2.758678000
6	-0.035490000	1.682316000	-1.498822000
6	-1.055324000	1.893492000	-2.426659000
1	-1.879489000	1.192425000	-2.476089000
6	-1.064136000	2.979161000	-3.294023000

1	-1.877706000	3.100062000	-3.999034000
6	-0.035604000	3.904845000	-3.262452000
1	-0.033589000	4.750396000	-3.938149000
6	0.992728000	3.729997000	-2.347119000
1	1.804200000	4.446560000	-2.302306000
6	0.980652000	2.643417000	-1.488267000
1	1.790207000	2.541640000	-0.774331000
6	-0.508919000	1.296076000	1.015013000
6	-1.824189000	1.748409000	1.164412000
1	-2.554470000	1.494546000	0.404118000
6	-2.234853000	2.514442000	2.242646000
1	-3.266449000	2.837600000	2.315342000
6	-1.325662000	2.872406000	3.228782000
1	-1.637695000	3.470781000	4.075126000
6	-0.011677000	2.453984000	3.106125000
1	0.715868000	2.729055000	3.860433000
6	0.379212000	1.682694000	2.018104000
1	1.416204000	1.377008000	1.954119000
**5-CTA^{+}/B(Ph)$_4^{-}$ 1:1 ΔG=1.83**			
7	-3.329068000	-4.648400000	-1.396709000
6	-2.507475000	-3.690014000	-2.218484000
6	-2.930864000	-3.538388000	-3.661824000
6	-1.864236000	-2.769185000	-4.423233000
6	-2.273742000	-2.452111000	-5.848615000
6	-1.164696000	-1.805605000	-6.668169000
6	-0.608674000	-0.505540000	-6.102805000
6	-1.637141000	0.600959000	-5.959170000
6	-1.049033000	1.900096000	-5.439747000
6	-3.160777000	-6.043438000	-1.879785000
6	-4.768290000	-4.283238000	-1.428626000
6	-2.844549000	-4.575009000	0.008343000
6	-2.072188000	3.021213000	-5.372239000
6	-1.589728000	4.275245000	-4.659862000
6	-0.346173000	4.903796000	-5.262963000
6	0.042158000	6.210807000	-4.595789000
6	1.312842000	6.812837000	-5.168982000
6	1.795864000	8.064339000	-4.452337000
6	0.859024000	9.255016000	-4.561622000
6	1.424210000	10.502279000	-3.908056000
1	-1.482132000	-4.049164000	-2.147774000
1	-2.566103000	-2.730082000	-1.708877000
1	-3.076681000	-4.507503000	-4.139081000
1	-3.877665000	-3.000121000	-3.721501000
1	-0.939859000	-3.351598000	-4.429620000
1	-1.639025000	-1.846314000	-3.886638000
1	-2.580630000	-3.375286000	-6.347271000
1	-3.156711000	-1.808385000	-5.835747000
1	-0.344500000	-2.521181000	-6.772690000
1	-1.538211000	-1.620011000	-7.678864000
1	-0.138932000	-0.690023000	-5.132798000
1	0.192998000	-0.158487000	-6.760202000

1	-2.437164000	0.281996000	-5.286433000
1	-2.109672000	0.780605000	-6.930462000
1	-0.215542000	2.196563000	-6.081023000
1	-0.622766000	1.734099000	-4.444915000
1	-3.695288000	-6.703969000	-1.204469000
1	-2.101956000	-6.282771000	-1.880450000
1	-3.570195000	-6.132093000	-2.878549000
1	-5.303239000	-4.953335000	-0.763190000
1	-5.142025000	-4.391842000	-2.439987000
1	-4.871832000	-3.256375000	-1.094046000
1	-1.797523000	-4.858701000	0.027677000
1	-2.962904000	-3.557077000	0.364514000
1	-3.433171000	-5.259335000	0.610998000
1	-2.385779000	3.277742000	-6.389028000
1	-2.966891000	2.652090000	-4.862661000
1	-2.396228000	5.013704000	-4.659361000
1	-1.393000000	4.036815000	-3.611144000
1	-0.507337000	5.076293000	-6.332282000
1	0.492724000	4.207210000	-5.188945000
1	0.180247000	6.041475000	-3.523443000
1	-0.785120000	6.918311000	-4.689493000
1	2.103968000	6.058751000	-5.131510000
1	1.159338000	7.039813000	-6.228781000
1	2.770992000	8.349608000	-4.856996000
1	1.961366000	7.833239000	-3.395021000
1	-0.103082000	9.014042000	-4.105674000
1	0.656191000	9.453861000	-5.617887000
1	1.611417000	10.335270000	-2.846147000
1	0.739107000	11.345522000	-3.996387000
1	2.370057000	10.790791000	-4.369126000
5	0.006269000	-0.100325000	-0.264724000
6	0.716533000	1.190504000	-1.003521000
6	0.057150000	2.417542000	-1.108600000
1	-0.977386000	2.487426000	-0.792980000
6	0.673238000	3.559231000	-1.596715000
1	0.119335000	4.487876000	-1.652994000
6	1.994034000	3.514802000	-2.015435000
1	2.477703000	4.401075000	-2.406616000
6	2.682493000	2.315630000	-1.917758000
1	3.718342000	2.259226000	-2.230494000
6	2.051102000	1.186334000	-1.414861000
1	2.623491000	0.269442000	-1.338906000
6	0.340062000	0.152842000	1.328720000
6	-0.444623000	0.992908000	2.122867000
1	-1.352223000	1.414155000	1.706895000
6	-0.108197000	1.318365000	3.428383000
1	-0.752668000	1.971653000	4.004427000
6	1.050816000	0.812033000	3.996673000
1	1.319158000	1.059549000	5.015721000
6	1.861480000	-0.012774000	3.232775000
1	2.776354000	-0.412774000	3.653388000

6	1.506031000	-0.327313000	1.928927000
1	2.166098000	-0.969667000	1.358173000
6	0.578388000	-1.547705000	-0.801779000
6	0.558120000	-2.690154000	0.002467000
1	0.222692000	-2.604582000	1.029605000
6	0.952408000	-3.936922000	-0.458507000
1	0.923239000	-4.792646000	0.205593000
6	1.381871000	-4.092243000	-1.767733000
1	1.693643000	-5.061796000	-2.134752000
6	1.392517000	-2.984925000	-2.600314000
1	1.708465000	-3.086144000	-3.631694000
6	0.993549000	-1.746337000	-2.119755000
1	1.005571000	-0.904066000	-2.801066000
6	-1.610334000	-0.160179000	-0.575281000
6	-2.544018000	-0.636760000	0.346142000
1	-2.210992000	-0.920050000	1.337563000
6	-3.893073000	-0.762897000	0.044856000
1	-4.580840000	-1.135825000	0.794570000
6	-4.361813000	-0.423502000	-1.214385000
1	-5.412628000	-0.520455000	-1.455882000
6	-3.458038000	0.032810000	-2.161497000
1	-3.799614000	0.291360000	-3.156655000
6	-2.115900000	0.157469000	-1.838497000
1	-1.434570000	0.513511000	-2.602254000
**1-CTA$^+$/B(Ph)$_4^-$ 2:1 ΔG=0.0**			
7	-1.693911000	-4.071261000	3.765787000
6	-1.307126000	-5.452181000	3.314019000
6	-2.296645000	-6.144288000	2.408487000
6	-1.815976000	-7.558129000	2.118964000
6	-2.708119000	-8.312384000	1.146061000
6	-2.659424000	-7.781681000	-0.275827000
6	-3.444605000	-8.629060000	-1.259175000
6	-3.412746000	-8.074890000	-2.670770000
6	-4.049512000	-8.989299000	-3.699348000
6	-2.031636000	-3.200037000	2.608393000
6	-0.528258000	-3.484581000	4.479359000
6	-2.847481000	-4.123564000	4.698981000
6	-3.985871000	-8.422466000	-5.108477000
6	-4.507456000	-9.359382000	-6.197746000
6	-3.436887000	-10.178846000	-6.905948000
6	-2.658476000	-11.140993000	-6.026211000
6	-1.663994000	-11.979091000	-6.809139000
6	-0.913101000	-12.979262000	-5.950075000
6	0.107911000	-13.793583000	-6.722522000
6	0.858990000	-14.779440000	-5.847760000
1	-1.143212000	-6.025408000	4.224762000
1	-0.352460000	-5.340436000	2.804214000
1	-3.284437000	-6.193308000	2.867619000
1	-2.393022000	-5.590305000	1.476856000
1	-1.756967000	-8.109867000	3.059846000
1	-0.798793000	-7.520456000	1.717927000

1	-2.406590000	-9.362187000	1.138350000
1	-3.740032000	-8.295059000	1.509307000
1	-3.034737000	-6.755701000	-0.309434000
1	-1.615412000	-7.734996000	-0.601538000
1	-3.039029000	-9.645102000	-1.260569000
1	-4.482609000	-8.715120000	-0.923529000
1	-2.374485000	-7.890909000	-2.960982000
1	-3.908831000	-7.099432000	-2.689844000
1	-5.095725000	-9.170072000	-3.434300000
1	-3.555753000	-9.962711000	-3.664019000
1	-2.950321000	-3.554286000	2.155458000
1	-1.215566000	-3.235879000	1.894814000
1	-2.164767000	-2.187678000	2.973959000
1	0.295056000	-3.395871000	3.777571000
1	-0.258436000	-4.141175000	5.300697000
1	-0.810049000	-2.505693000	4.852732000
1	-3.707192000	-4.533952000	4.182655000
1	-2.580938000	-4.746793000	5.546760000
1	-3.064509000	-3.112263000	5.028244000
1	-4.556736000	-7.490695000	-5.124128000
1	-2.952667000	-8.142091000	-5.337667000
1	-5.256691000	-10.032124000	-5.769359000
1	-5.030785000	-8.773203000	-6.955665000
1	-2.733810000	-9.494112000	-7.390917000
1	-3.909583000	-10.747699000	-7.711728000
1	-3.357109000	-11.801749000	-5.503319000
1	-2.120124000	-10.587898000	-5.252468000
1	-2.188203000	-12.511765000	-7.608666000
1	-0.946515000	-11.316434000	-7.303523000
1	-1.628968000	-13.656230000	-5.473390000
1	-0.407458000	-12.450330000	-5.137290000
1	0.818402000	-13.115048000	-7.202405000
1	-0.395464000	-14.330102000	-7.531380000
1	0.173767000	-15.488663000	-5.380929000
1	1.394213000	-14.262972000	-5.049354000
1	1.588054000	-15.350385000	-6.422787000
7	3.094432000	3.507301000	-3.469694000
6	2.946982000	2.607640000	-4.665126000
6	3.286022000	1.154748000	-4.436585000
6	3.065181000	0.375085000	-5.724095000
6	3.402374000	-1.102693000	-5.603501000
6	2.540661000	-1.846311000	-4.599432000
6	2.752732000	-3.348092000	-4.605961000
6	1.912128000	-4.048085000	-3.552473000
6	2.065255000	-5.560489000	-3.512319000
6	4.499688000	3.532405000	-2.990373000
6	2.198927000	3.076109000	-2.362798000
6	2.706684000	4.880097000	-3.888678000
6	1.484425000	-6.270354000	-4.724108000
6	1.617646000	-7.785249000	-4.686207000
6	0.836347000	-8.454698000	-3.568775000

6	0.873544000	-9.970277000	-3.653823000
6	0.045309000	-10.681607000	-2.595217000
6	0.512899000	-10.447109000	-1.169002000
6	-0.215209000	-11.310745000	-0.155205000
6	0.245869000	-11.066412000	1.268997000
1	3.583199000	3.035314000	-5.437826000
1	1.909645000	2.707443000	-4.980611000
1	4.325370000	1.040824000	-4.126699000
1	2.659716000	0.744500000	-3.647099000
1	3.670550000	0.818791000	-6.517897000
1	2.021604000	0.479804000	-6.033757000
1	3.284662000	-1.565947000	-6.586465000
1	4.457044000	-1.216479000	-5.335304000
1	2.734150000	-1.469880000	-3.592487000
1	1.487034000	-1.634247000	-4.805775000
1	2.518282000	-3.737150000	-5.599620000
1	3.810040000	-3.570338000	-4.429818000
1	0.857523000	-3.800473000	-3.710657000
1	2.170827000	-3.638155000	-2.572201000
1	1.578082000	-5.930363000	-2.608537000
1	3.125353000	-5.817734000	-3.416298000
1	5.143475000	3.820315000	-3.815783000
1	4.768887000	2.550624000	-2.620671000
1	4.570457000	4.258375000	-2.186551000
1	2.524510000	2.116125000	-1.982459000
1	1.185355000	3.006151000	-2.743385000
1	2.258723000	3.819748000	-1.574926000
1	3.355611000	5.192894000	-4.700565000
1	1.672572000	4.861764000	-4.217851000
1	2.818915000	5.545682000	-3.038990000
1	0.425384000	-6.006899000	-4.815258000
1	1.969164000	-5.900764000	-5.629761000
1	1.277215000	-8.191681000	-5.642729000
1	2.675087000	-8.055319000	-4.600771000
1	-0.204046000	-8.117514000	-3.609661000
1	1.224295000	-8.131949000	-2.601718000
1	1.912334000	-10.309105000	-3.584896000
1	0.519821000	-10.273095000	-4.643326000
1	-1.001871000	-10.375343000	-2.687688000
1	0.066069000	-11.756161000	-2.797090000
1	0.380579000	-9.396439000	-0.898242000
1	1.587651000	-10.646947000	-1.104485000
1	-0.070827000	-12.363779000	-0.411455000
1	-1.289596000	-11.123973000	-0.229370000
1	0.088181000	-10.026418000	1.558657000
1	1.310165000	-11.281802000	1.377356000
1	-0.294353000	-11.694349000	1.977779000
5	0.190140000	-0.203395000	0.027123000
6	-0.388155000	0.568478000	-1.309914000
6	-1.052159000	1.795147000	-1.248629000
1	-1.272345000	2.229277000	-0.280713000

6	-1.447881000	2.488848000	-2.383920000
1	-1.966117000	3.435303000	-2.284831000
6	-1.174454000	1.979422000	-3.643492000
1	-1.476604000	2.516691000	-4.533404000
6	-0.494783000	0.774535000	-3.741588000
1	-0.254986000	0.365443000	-4.715866000
6	-0.117416000	0.092772000	-2.595182000
1	0.412999000	-0.845264000	-2.706058000
6	1.724912000	0.378238000	0.179638000
6	2.009530000	1.552980000	0.879282000
1	1.210087000	2.057498000	1.408914000
6	3.279890000	2.108524000	0.923676000
1	3.450712000	3.020890000	1.482569000
6	4.328831000	1.504684000	0.248964000
1	5.322251000	1.933710000	0.276248000
6	4.078505000	0.348421000	-0.473361000
1	4.880553000	-0.132168000	-1.021199000
6	2.802255000	-0.194226000	-0.501252000
1	2.641971000	-1.095992000	-1.080283000
6	0.159744000	-1.843615000	-0.137465000
6	1.066297000	-2.670551000	0.529991000
1	1.866908000	-2.218889000	1.104141000
6	0.985432000	-4.055424000	0.490791000
1	1.715117000	-4.653896000	1.023311000
6	-0.029946000	-4.674681000	-0.220610000
1	-0.099561000	-5.754237000	-0.255472000
6	-0.956720000	-3.885171000	-0.882078000
1	-1.763593000	-4.349451000	-1.437033000
6	-0.855163000	-2.501865000	-0.835935000
1	-1.597728000	-1.914333000	-1.363372000
6	-0.728096000	0.083613000	1.365491000
6	-0.186650000	0.085263000	2.652745000
1	0.882524000	-0.041205000	2.774916000
6	-0.962527000	0.247816000	3.791228000
1	-0.494043000	0.247510000	4.768317000
6	-2.335119000	0.407585000	3.682928000
1	-2.947192000	0.537513000	4.566283000
6	-2.910323000	0.385254000	2.421599000
1	-3.983131000	0.493313000	2.315210000
6	-2.116658000	0.221225000	1.295992000
1	-2.600233000	0.203426000	0.326541000
**2-CTA$^+$/B(Ph)$_4^-$ 2:1 ΔG=0.79**			
7	-5.520302000	-1.797819000	0.866935000
6	-4.571129000	-1.887852000	-0.299188000
6	-5.223665000	-2.110282000	-1.645934000
6	-4.169854000	-2.417151000	-2.703434000
6	-3.657144000	-3.846384000	-2.682522000
6	-2.488691000	-4.046384000	-3.630117000
6	-1.946182000	-5.463895000	-3.629383000
6	-0.764128000	-5.679127000	-4.563052000
6	0.488600000	-4.914292000	-4.170866000

6	-6.577167000	-0.783667000	0.623018000
6	-4.733169000	-1.392015000	2.061880000
6	-6.148538000	-3.115710000	1.140675000
6	1.685302000	-5.234339000	-5.048653000
6	2.935803000	-4.478468000	-4.639358000
6	4.152484000	-4.808560000	-5.487829000
6	5.440762000	-4.172308000	-4.985862000
6	5.490459000	-2.660063000	-5.121649000
6	6.708588000	-2.042916000	-4.459273000
6	6.740504000	-0.528328000	-4.552197000
6	7.944710000	0.084023000	-3.863104000
1	-3.879217000	-2.688284000	-0.046808000
1	-4.016843000	-0.951027000	-0.307399000
1	-5.943226000	-2.928694000	-1.609761000
1	-5.768883000	-1.212981000	-1.935274000
1	-3.330180000	-1.730029000	-2.581433000
1	-4.592827000	-2.207008000	-3.687591000
1	-3.342919000	-4.119644000	-1.672454000
1	-4.471302000	-4.527746000	-2.945799000
1	-2.794199000	-3.778224000	-4.646628000
1	-1.698612000	-3.344378000	-3.354619000
1	-1.652504000	-5.737382000	-2.611098000
1	-2.750602000	-6.149710000	-3.908550000
1	-1.052644000	-5.403834000	-5.582452000
1	-0.527039000	-6.746047000	-4.592023000
1	0.735932000	-5.143764000	-3.129103000
1	0.300058000	-3.838655000	-4.209040000
1	-6.100640000	0.156099000	0.363651000
1	-7.159053000	-0.674050000	1.532643000
1	-7.217067000	-1.119942000	-0.184421000
1	-3.947634000	-2.123276000	2.221273000
1	-5.398936000	-1.357060000	2.918432000
1	-4.301337000	-0.413828000	1.876392000
1	-5.361935000	-3.841475000	1.321459000
1	-6.778264000	-3.016671000	2.019059000
1	-6.749842000	-3.413049000	0.289874000
1	1.883437000	-6.309946000	-5.012567000
1	1.443490000	-5.003025000	-6.090720000
1	2.729806000	-3.406667000	-4.679857000
1	3.162561000	-4.704852000	-3.592347000
1	3.970740000	-4.503867000	-6.523322000
1	4.280143000	-5.894230000	-5.507767000
1	6.290498000	-4.602673000	-5.522434000
1	5.577801000	-4.441838000	-3.933777000
1	5.474009000	-2.391613000	-6.182692000
1	4.592742000	-2.219681000	-4.684829000
1	7.618337000	-2.453682000	-4.908458000
1	6.729888000	-2.337492000	-3.405173000
1	5.823764000	-0.123837000	-4.114183000
1	6.729747000	-0.233180000	-5.604257000
1	8.874948000	-0.284025000	-4.299073000

1	7.956158000	-0.166312000	-2.800952000
1	7.943857000	1.170531000	-3.949074000
7	4.317351000	3.411307000	-1.806158000
6	4.900562000	2.994100000	-3.128416000
6	5.159737000	4.109156000	-4.115212000
6	5.864025000	3.572640000	-5.355677000
6	5.038767000	2.587261000	-6.165638000
6	5.758700000	2.120413000	-7.418854000
6	5.110091000	0.953344000	-8.151692000
6	3.740037000	1.223550000	-8.768654000
6	2.547064000	0.997878000	-7.852582000
6	5.280479000	4.231523000	-1.028423000
6	4.019834000	2.170342000	-1.038520000
6	3.054905000	4.172530000	-1.991067000
6	1.223564000	1.188758000	-8.570319000
6	0.010686000	1.072193000	-7.665015000
6	-1.295600000	1.206505000	-8.427683000
6	-2.549607000	1.135976000	-7.570459000
6	-2.713178000	2.296975000	-6.603768000
6	-4.077516000	2.326110000	-5.939627000
6	-4.252813000	3.482939000	-4.973198000
6	-5.638030000	3.535068000	-4.357343000
1	4.196718000	2.273538000	-3.534783000
1	5.825294000	2.471150000	-2.889461000
1	4.222070000	4.578997000	-4.413124000
1	5.783711000	4.880673000	-3.665724000
1	6.807576000	3.105116000	-5.061697000
1	6.127627000	4.422171000	-5.989157000
1	4.795922000	1.711591000	-5.560665000
1	4.089048000	3.057567000	-6.428138000
1	5.867527000	2.962680000	-8.109003000
1	6.774670000	1.824463000	-7.141592000
1	5.793109000	0.647057000	-8.946868000
1	5.036572000	0.097276000	-7.473588000
1	3.616062000	0.573507000	-9.639012000
1	3.712244000	2.247324000	-9.155351000
1	2.589071000	1.671075000	-6.995373000
1	2.594922000	-0.016549000	-7.444726000
1	4.840719000	4.439512000	-0.058122000
1	5.470828000	5.160800000	-1.552201000
1	6.199929000	3.666431000	-0.911138000
1	3.284066000	1.589876000	-1.584967000
1	3.631445000	2.444711000	-0.063277000
1	4.941762000	1.606499000	-0.931971000
1	3.276568000	5.125833000	-2.456291000
1	2.386082000	3.589622000	-2.616343000
1	2.610312000	4.330682000	-1.014303000
1	1.213735000	2.172693000	-9.049758000
1	1.143940000	0.456831000	-9.380251000
1	0.029380000	0.107942000	-7.146578000
1	0.073228000	1.838358000	-6.888513000

1	-1.338303000	0.420075000	-9.186117000
1	-1.294119000	2.154962000	-8.974358000
1	-2.561320000	0.193820000	-7.013337000
1	-3.420580000	1.109065000	-8.230953000
1	-1.939546000	2.257063000	-5.832509000
1	-2.559241000	3.236344000	-7.144458000
1	-4.244079000	1.382296000	-5.410946000
1	-4.851441000	2.382098000	-6.711353000
1	-4.051697000	4.420718000	-5.498255000
1	-3.501439000	3.407877000	-4.182968000
1	-5.862112000	2.614036000	-3.817410000
1	-6.401945000	3.660888000	-5.126286000
1	-5.732760000	4.363190000	-3.654710000
5	-0.169217000	0.321274000	-0.318759000
6	-0.501932000	-1.147923000	0.350471000
6	-0.766974000	-1.299728000	1.713206000
1	-0.675527000	-0.440188000	2.366910000
6	-1.141925000	-2.513154000	2.270795000
1	-1.335819000	-2.580967000	3.334673000
6	-1.272769000	-3.637722000	1.470806000
1	-1.562028000	-4.588849000	1.899283000
6	-1.031683000	-3.519665000	0.111159000
1	-1.134170000	-4.383454000	-0.534259000
6	-0.661019000	-2.296701000	-0.427438000
1	-0.485291000	-2.238686000	-1.494968000
6	-1.656639000	0.955316000	-0.634587000
6	-2.392840000	1.606982000	0.358983000
1	-1.938101000	1.769115000	1.329281000
6	-3.687945000	2.057741000	0.155967000
1	-4.217826000	2.559214000	0.957127000
6	-4.308210000	1.865413000	-1.069577000
1	-5.317743000	2.217533000	-1.239254000
6	-3.615905000	1.204369000	-2.069527000
1	-4.089522000	1.024790000	-3.026345000
6	-2.320712000	0.760310000	-1.846401000
1	-1.813760000	0.237158000	-2.648028000
6	0.773550000	0.178518000	-1.661657000
6	0.706614000	1.065457000	-2.737983000
1	-0.039369000	1.851058000	-2.729539000
6	1.559799000	0.981734000	-3.830068000
1	1.461832000	1.687409000	-4.646454000
6	2.538395000	0.002414000	-3.877280000
1	3.206094000	-0.069853000	-4.726121000
6	2.657097000	-0.872117000	-2.808386000
1	3.429189000	-1.631445000	-2.810552000
6	1.789518000	-0.778306000	-1.732253000
1	1.909531000	-1.478812000	-0.914462000
6	0.707562000	1.285347000	0.690025000
6	0.613897000	2.677682000	0.648430000
1	-0.107576000	3.137698000	-0.016876000
6	1.407249000	3.504770000	1.430487000

1	1.292613000	4.580396000	1.366681000
6	2.346602000	2.959264000	2.291304000
1	2.968359000	3.597628000	2.905919000
6	2.481488000	1.580134000	2.342611000
1	3.217892000	1.133576000	3.000061000
6	1.677452000	0.770686000	1.553646000
1	1.809802000	-0.303029000	1.616992000
**3-CTA$^+$/B(Ph)$_4^-$ 2:1 ΔG=1.28**			
7	-3.388971000	-0.030541000	4.596864000
6	-3.230255000	-0.433499000	6.036993000
6	-2.001016000	-1.252443000	6.347093000
6	-2.058970000	-1.783223000	7.769251000
6	-0.839043000	-2.617374000	8.113153000
6	-0.919938000	-3.264585000	9.483185000
6	0.296460000	-4.116721000	9.795487000
6	0.260164000	-4.802466000	11.153586000
6	-0.914605000	-5.746498000	11.374604000
6	-4.599397000	0.827869000	4.498637000
6	-3.585017000	-1.225467000	3.734631000
6	-2.210697000	0.743083000	4.119284000
6	-1.035282000	-6.851419000	10.339616000
6	-2.150056000	-7.831377000	10.663804000
6	-2.311015000	-8.967213000	9.663801000
6	-2.783356000	-8.522853000	8.288996000
6	-2.973509000	-9.663262000	7.299144000
6	-4.126443000	-10.595923000	7.633623000
6	-4.401901000	-11.643538000	6.564781000
6	-3.280033000	-12.650899000	6.385070000
1	-4.134396000	-0.985503000	6.286679000
1	-3.228110000	0.492681000	6.608557000
1	-1.917441000	-2.098784000	5.666390000
1	-1.102436000	-0.649837000	6.221486000
1	-2.959568000	-2.391230000	7.890233000
1	-2.148233000	-0.951735000	8.473561000
1	-0.717702000	-3.399756000	7.357576000
1	0.057565000	-1.993382000	8.052390000
1	-1.033208000	-2.491750000	10.250039000
1	-1.824550000	-3.876221000	9.528580000
1	0.415290000	-4.866264000	9.009597000
1	1.190530000	-3.489830000	9.744208000
1	0.251694000	-4.041001000	11.938451000
1	1.190198000	-5.363123000	11.283316000
1	-1.848697000	-5.179057000	11.399612000
1	-0.813498000	-6.197595000	12.365529000
1	-4.736229000	1.116275000	3.461800000
1	-4.450382000	1.706666000	5.117753000
1	-5.455380000	0.260132000	4.849357000
1	-3.816545000	-0.885659000	2.731389000
1	-4.409367000	-1.807363000	4.134969000
1	-2.675190000	-1.813081000	3.717979000
1	-2.419576000	1.094965000	3.113621000

1	-2.059681000	1.583347000	4.790629000
1	-1.338416000	0.099908000	4.109684000
1	-1.206628000	-6.411039000	9.356006000
1	-0.085696000	-7.392970000	10.271038000
1	-1.964720000	-8.255433000	11.654448000
1	-3.096038000	-7.285459000	10.740386000
1	-3.019193000	-9.689942000	10.073416000
1	-1.358918000	-9.497839000	9.560658000
1	-2.064366000	-7.816729000	7.869496000
1	-3.724954000	-7.973672000	8.393442000
1	-3.147235000	-9.242446000	6.304411000
1	-2.041913000	-10.230668000	7.230852000
1	-5.028487000	-9.995258000	7.781535000
1	-3.936907000	-11.103304000	8.582988000
1	-4.598500000	-11.141994000	5.612952000
1	-5.319720000	-12.176565000	6.823113000
1	-3.057076000	-13.155238000	7.327008000
1	-2.362257000	-12.176681000	6.038494000
1	-3.551475000	-13.413964000	5.655341000
7	2.402278000	-1.025494000	-5.193030000
6	2.695044000	-1.482307000	-3.788625000
6	4.103690000	-1.970339000	-3.538309000
6	4.183368000	-2.547441000	-2.134501000
6	5.597987000	-2.892393000	-1.707702000
6	5.683055000	-3.442175000	-0.290302000
6	5.235360000	-2.450952000	0.769250000
6	5.397066000	-2.940280000	2.196419000
6	4.933351000	-1.896841000	3.198369000
6	3.339691000	0.047241000	-5.612907000
6	1.016134000	-0.485987000	-5.215216000
6	2.481187000	-2.160702000	-6.148072000
6	5.067016000	-2.293379000	4.659682000
6	4.139439000	-3.415325000	5.094353000
6	4.176244000	-3.653721000	6.594640000
6	3.091057000	-4.585364000	7.109664000
6	3.183097000	-6.013837000	6.603904000
6	2.108180000	-6.912170000	7.188284000
6	2.190238000	-8.348826000	6.706576000
6	1.095768000	-9.225051000	7.285763000
1	1.968818000	-2.264904000	-3.576503000
1	2.475070000	-0.631606000	-3.145656000
1	4.397986000	-2.730391000	-4.262054000
1	4.809911000	-1.143678000	-3.624947000
1	3.555871000	-3.439435000	-2.072332000
1	3.755803000	-1.822356000	-1.442959000
1	6.017322000	-3.621651000	-2.405705000
1	6.224792000	-1.998491000	-1.783035000
1	5.078879000	-4.351789000	-0.216201000
1	6.714465000	-3.741579000	-0.086608000
1	5.795533000	-1.518314000	0.643642000
1	4.185113000	-2.196644000	0.615047000

1	6.445468000	-3.189903000	2.387701000
1	4.830183000	-3.865869000	2.325619000
1	3.887889000	-1.645933000	2.992883000
1	5.503228000	-0.979014000	3.027978000
1	3.302429000	0.844317000	-4.877324000
1	3.022902000	0.413658000	-6.584362000
1	4.341832000	-0.359274000	-5.681655000
1	0.335415000	-1.271210000	-4.903642000
1	0.787999000	-0.164411000	-6.226338000
1	0.959659000	0.352413000	-4.528679000
1	1.804288000	-2.939861000	-5.811983000
1	2.185046000	-1.801672000	-7.128696000
1	3.498156000	-2.532122000	-6.185004000
1	6.104243000	-2.572699000	4.870819000
1	4.858261000	-1.415229000	5.277368000
1	3.116530000	-3.163065000	4.799116000
1	4.393581000	-4.335184000	4.564813000
1	4.074204000	-2.690203000	7.101893000
1	5.158769000	-4.043611000	6.878859000
1	2.112094000	-4.173163000	6.843544000
1	3.124931000	-4.598701000	8.202144000
1	3.107222000	-6.031130000	5.513757000
1	4.169563000	-6.420370000	6.849312000
1	1.123226000	-6.504802000	6.938096000
1	2.174215000	-6.894000000	8.280841000
1	3.168945000	-8.760523000	6.967521000
1	2.133495000	-8.365640000	5.614774000
1	1.168700000	-10.251441000	6.925482000
1	0.110217000	-8.845446000	7.013316000
1	1.148632000	-9.249206000	8.375462000
5	0.166502000	-0.139968000	0.162331000
6	-0.035395000	-1.484016000	-0.771462000
6	0.740330000	-2.632908000	-0.601701000
1	1.453757000	-2.672019000	0.212684000
6	0.642141000	-3.733587000	-1.439993000
1	1.264835000	-4.602379000	-1.263780000
6	-0.241710000	-3.721735000	-2.506989000
1	-0.324754000	-4.577422000	-3.164857000
6	-1.007729000	-2.586704000	-2.723343000
1	-1.693520000	-2.548606000	-3.561348000
6	-0.897085000	-1.497781000	-1.871566000
1	-1.506568000	-0.625277000	-2.074449000
6	-1.217743000	0.741267000	0.308926000
6	-2.485468000	0.157301000	0.326431000
1	-2.576390000	-0.910849000	0.168032000
6	-3.644268000	0.891134000	0.538788000
1	-4.606648000	0.393182000	0.536008000
6	-3.573863000	2.256623000	0.763379000
1	-4.473163000	2.834792000	0.933026000
6	-2.328547000	2.866554000	0.774939000
1	-2.249767000	3.931446000	0.958431000

6	-1.183651000	2.117252000	0.549869000
1	-0.226298000	2.624957000	0.562073000
6	1.348489000	0.703204000	-0.618108000
6	1.063265000	1.551961000	-1.690656000
1	0.028595000	1.731746000	-1.959464000
6	2.054552000	2.177629000	-2.431617000
1	1.784041000	2.828099000	-3.254975000
6	3.391239000	1.966795000	-2.126957000
1	4.170426000	2.448532000	-2.703860000
6	3.710119000	1.121155000	-1.076895000
1	4.747980000	0.932233000	-0.829055000
6	2.702971000	0.509506000	-0.344509000
1	2.983607000	-0.147685000	0.469383000
6	0.589314000	-0.493650000	1.716389000
6	0.199274000	-1.666466000	2.366136000
1	-0.353994000	-2.417315000	1.815024000
6	0.493810000	-1.916919000	3.699248000
1	0.176373000	-2.846632000	4.156430000
6	1.186029000	-0.980392000	4.448572000
1	1.421678000	-1.171468000	5.487795000
6	1.563505000	0.208464000	3.844660000
1	2.096121000	0.959767000	4.415566000
6	1.267173000	0.436326000	2.509402000
1	1.579134000	1.375216000	2.067013000
**4-CTA$^+$/B(Ph)$_4^-$ 2:1 ΔG=1.40**			
7	4.407528000	1.150245000	3.201631000
6	3.242546000	0.200120000	3.288482000
6	3.523966000	-1.116243000	3.981491000
6	2.216864000	-1.733126000	4.450876000
6	2.382848000	-3.127439000	5.035094000
6	1.254687000	-3.515203000	5.979154000
6	-0.135880000	-3.530118000	5.364974000
6	-1.210201000	-3.725477000	6.421658000
6	-2.637132000	-3.791968000	5.900266000
6	5.594048000	0.489877000	2.601200000
6	4.759158000	1.681258000	4.544401000
6	4.000123000	2.290535000	2.337742000
6	-3.073349000	-2.573289000	5.100564000
6	-4.586338000	-2.459308000	4.987410000
6	-5.067639000	-1.495336000	3.913938000
6	-4.498037000	-0.090730000	4.003348000
6	-4.998116000	0.809458000	2.886535000
6	-4.246518000	2.123032000	2.779631000
6	-4.732259000	2.998009000	1.638201000
6	-3.935566000	4.281044000	1.496209000
1	2.917046000	0.025426000	2.264785000
1	2.457946000	0.752411000	3.802502000
1	4.037944000	-1.795147000	3.299923000
1	4.171282000	-0.981259000	4.847864000
1	1.506106000	-1.754131000	3.624300000
1	1.779924000	-1.074228000	5.205878000

1	2.468034000	-3.857422000	4.225053000
1	3.324065000	-3.174867000	5.588939000
1	1.464256000	-4.498307000	6.408890000
1	1.256154000	-2.811539000	6.817747000
1	-0.316011000	-2.592129000	4.837225000
1	-0.200196000	-4.323903000	4.613636000
1	-1.139684000	-2.902110000	7.139677000
1	-0.994543000	-4.637530000	6.985519000
1	-3.307629000	-3.911294000	6.756015000
1	-2.768065000	-4.688909000	5.287366000
1	5.309798000	0.085345000	1.635843000
1	5.932254000	-0.302319000	3.259314000
1	6.375449000	1.234095000	2.485041000
1	3.878860000	2.153870000	4.968321000
1	5.556294000	2.408048000	4.424016000
1	5.095955000	0.869928000	5.178161000
1	3.122162000	2.757171000	2.773298000
1	4.821341000	2.998441000	2.291123000
1	3.770462000	1.909916000	1.348004000
1	-2.673942000	-1.672277000	5.572978000
1	-2.634539000	-2.609038000	4.099530000
1	-5.003174000	-3.448070000	4.775155000
1	-4.991419000	-2.164279000	5.960252000
1	-6.159538000	-1.444777000	3.940944000
1	-4.805813000	-1.901560000	2.933633000
1	-3.408940000	-0.138820000	3.950338000
1	-4.739671000	0.351598000	4.975202000
1	-4.905511000	0.275068000	1.936070000
1	-6.066130000	1.004832000	3.018790000
1	-3.180742000	1.913797000	2.642361000
1	-4.330470000	2.674355000	3.721664000
1	-5.789316000	3.234527000	1.787901000
1	-4.677408000	2.430112000	0.705166000
1	-2.880595000	4.065483000	1.317665000
1	-4.000411000	4.885062000	2.402768000
1	-4.297254000	4.887347000	0.665513000
7	-3.799582000	-1.713717000	-4.274202000
6	-3.984887000	-0.978756000	-2.971110000
6	-5.409738000	-0.917886000	-2.470099000
6	-5.507399000	0.037806000	-1.290866000
6	-6.902358000	0.129712000	-0.692064000
6	-7.390548000	-1.155562000	-0.042685000
6	-8.768729000	-1.040242000	0.593272000
6	-8.809169000	-0.182556000	1.845707000
6	-10.188364000	-0.107780000	2.473658000
6	-4.375466000	-3.081122000	-4.204851000
6	-2.339943000	-1.824009000	-4.537195000
6	-4.423060000	-0.967834000	-5.397963000
6	-10.233086000	0.760417000	3.717220000
6	-11.607627000	0.825208000	4.356920000
6	-11.650427000	1.722654000	5.580607000

6	-12.988126000	1.729571000	6.306375000
6	-14.177781000	2.184879000	5.468891000
6	-14.032204000	3.557900000	4.828868000
6	-13.837719000	4.692982000	5.818690000
6	-13.788100000	6.051086000	5.143937000
1	-3.580140000	0.018696000	-3.133821000
1	-3.352360000	-1.488116000	-2.246443000
1	-6.089323000	-0.577361000	-3.251995000
1	-5.733257000	-1.912703000	-2.168697000
1	-5.192648000	1.030521000	-1.618747000
1	-4.800104000	-0.269336000	-0.517632000
1	-6.899040000	0.932115000	0.047660000
1	-7.612403000	0.431383000	-1.468568000
1	-7.419045000	-1.952054000	-0.788201000
1	-6.666997000	-1.471753000	0.715867000
1	-9.473912000	-0.643273000	-0.143968000
1	-9.126961000	-2.041643000	0.846766000
1	-8.466405000	0.830167000	1.620177000
1	-8.103208000	-0.592438000	2.573902000
1	-10.527605000	-1.117596000	2.724674000
1	-10.900556000	0.278914000	1.738107000
1	-3.946346000	-3.593972000	-3.349876000
1	-4.123350000	-3.602263000	-5.122848000
1	-5.452176000	-3.011808000	-4.105264000
1	-1.920361000	-0.825280000	-4.585085000
1	-2.198358000	-2.340308000	-5.481259000
1	-1.883210000	-2.382080000	-3.726787000
1	-3.999318000	0.031171000	-5.424394000
1	-4.201113000	-1.495538000	-6.319967000
1	-5.495314000	-0.921276000	-5.251550000
1	-9.510752000	0.383549000	4.448027000
1	-9.906859000	1.773242000	3.460945000
1	-12.328080000	1.175632000	3.613354000
1	-11.924850000	-0.184341000	4.637445000
1	-11.381432000	2.741255000	5.287457000
1	-10.876967000	1.399507000	6.282785000
1	-12.903498000	2.362422000	7.192451000
1	-13.194145000	0.721556000	6.677042000
1	-14.371360000	1.450523000	4.684048000
1	-15.067199000	2.184394000	6.105169000
1	-14.927150000	3.760135000	4.233734000
1	-13.198014000	3.552902000	4.121632000
1	-12.916364000	4.537778000	6.383915000
1	-14.651331000	4.674813000	6.549436000
1	-12.965720000	6.100378000	4.428502000
1	-13.647436000	6.853363000	5.868389000
1	-14.712191000	6.251148000	4.599379000
5	-0.339154000	-0.219015000	0.069269000
6	-0.873319000	0.781582000	-1.126859000
6	-0.410251000	0.711188000	-2.441909000
1	0.384955000	0.016256000	-2.684865000

6	-0.926753000	1.501315000	-3.459216000
1	-0.528343000	1.416561000	-4.463295000
6	-1.953718000	2.394239000	-3.197201000
1	-2.360849000	3.013367000	-3.986342000
6	-2.453430000	2.473997000	-1.906352000
1	-3.263922000	3.156880000	-1.680016000
6	-1.918800000	1.681080000	-0.903222000
1	-2.330880000	1.768766000	0.095103000
6	-1.315649000	-1.542881000	-0.049851000
6	-1.004827000	-2.596569000	-0.914650000
1	-0.064038000	-2.576085000	-1.451701000
6	-1.851580000	-3.674780000	-1.119013000
1	-1.562424000	-4.467536000	-1.798672000
6	-3.070642000	-3.736160000	-0.461495000
1	-3.740982000	-4.571964000	-0.616534000
6	-3.419813000	-2.701073000	0.389407000
1	-4.376503000	-2.724828000	0.895741000
6	-2.555674000	-1.632323000	0.584872000
1	-2.865052000	-0.838148000	1.254195000
6	-0.400889000	0.529693000	1.535286000
6	-0.672708000	-0.136430000	2.729409000
1	-0.941973000	-1.185659000	2.700510000
6	-0.608738000	0.490681000	3.966281000
1	-0.829745000	-0.070234000	4.866105000
6	-0.259827000	1.826597000	4.055152000
1	-0.209997000	2.321609000	5.016676000
6	0.032826000	2.518015000	2.888051000
1	0.318366000	3.562306000	2.934382000
6	-0.037112000	1.874588000	1.663661000
1	0.200868000	2.441159000	0.770723000
6	1.245492000	-0.638673000	-0.096066000
6	1.738619000	-1.828208000	0.448085000
1	1.048728000	-2.507731000	0.934763000
6	3.076886000	-2.182532000	0.386473000
1	3.408730000	-3.118233000	0.820205000
6	3.991090000	-1.340886000	-0.228566000
1	5.037358000	-1.612503000	-0.288002000
6	3.543369000	-0.138209000	-0.751098000
1	4.244715000	0.542636000	-1.218756000
6	2.199029000	0.199253000	-0.675981000
1	1.884825000	1.149028000	-1.091900000
**5-CTA$^+$/B(Ph)$_4^-$ 2:1 ΔG=1.77**			
7	-3.171559000	-2.376971000	4.136610000
6	-2.350399000	-1.156212000	4.456968000
6	-3.020607000	-0.122362000	5.330419000
6	-2.013724000	0.960057000	5.681165000
6	-2.597248000	2.058006000	6.551725000
6	-1.596823000	3.170026000	6.832040000
6	-1.408658000	4.135045000	5.668594000
6	-0.136077000	4.963939000	5.778814000
6	1.100434000	4.196205000	5.343666000

6	-3.486103000	-3.148184000	5.366936000
6	-2.362081000	-3.238364000	3.232339000
6	-4.435968000	-2.009927000	3.448602000
6	2.407418000	4.906379000	5.641822000
6	3.606594000	4.189744000	5.049136000
6	4.939193000	4.781870000	5.473532000
6	6.135263000	4.232071000	4.710406000
6	6.314543000	2.727451000	4.815724000
6	7.583045000	2.234270000	4.144150000
6	7.745797000	0.726027000	4.197833000
6	9.062944000	0.251840000	3.614019000
1	-2.076119000	-0.719663000	3.497975000
1	-1.444482000	-1.529588000	4.930198000
1	-3.867618000	0.327381000	4.811159000
1	-3.398151000	-0.568367000	6.250723000
1	-1.612091000	1.385118000	4.759861000
1	-1.167764000	0.505896000	6.203462000
1	-3.487205000	2.477328000	6.072417000
1	-2.934118000	1.618286000	7.493585000
1	-1.914621000	3.741823000	7.706931000
1	-0.638305000	2.717251000	7.099920000
1	-1.387211000	3.585961000	4.723235000
1	-2.281307000	4.790368000	5.617277000
1	-0.014867000	5.306487000	6.811504000
1	-0.223310000	5.865498000	5.167531000
1	1.031560000	3.999965000	4.268838000
1	1.117177000	3.214980000	5.825910000
1	-3.983938000	-4.066709000	5.072886000
1	-4.141455000	-2.563396000	6.001236000
1	-2.557421000	-3.371341000	5.882565000
1	-1.452691000	-3.522677000	3.751493000
1	-2.123873000	-2.673277000	2.337171000
1	-2.944613000	-4.119033000	2.981717000
1	-4.194798000	-1.419056000	2.571513000
1	-4.942672000	-2.925409000	3.160087000
1	-5.060323000	-1.442049000	4.128230000
1	2.369384000	5.929748000	5.255550000
1	2.529798000	4.994124000	6.725914000
1	3.564529000	3.134924000	5.332800000
1	3.534340000	4.211709000	3.957946000
1	5.082443000	4.613588000	6.545445000
1	4.905792000	5.866554000	5.337942000
1	7.042833000	4.721937000	5.074039000
1	6.044520000	4.507084000	3.655008000
1	6.328097000	2.437506000	5.871405000
1	5.456021000	2.218462000	4.370380000
1	8.449073000	2.707715000	4.617079000
1	7.591041000	2.559862000	3.098691000
1	6.915340000	0.256087000	3.663132000
1	7.667278000	0.394131000	5.236665000
1	9.160397000	0.553090000	2.569880000

1	9.151664000	-0.833841000	3.655522000
1	9.906373000	0.676673000	4.160715000
7	4.853815000	1.843512000	-2.998657000
6	5.084397000	1.136468000	-1.688342000
6	6.482845000	1.265806000	-1.125900000
6	6.656229000	0.359143000	0.086759000
6	6.922030000	-1.099180000	-0.244652000
6	6.874288000	-1.969478000	0.996750000
6	7.169520000	-3.437719000	0.743980000
6	6.909827000	-4.315217000	1.961787000
6	5.432265000	-4.517634000	2.252824000
6	3.396208000	1.799975000	-3.290928000
6	5.578484000	1.162624000	-4.102317000
6	5.281831000	3.264054000	-2.925864000
6	5.150984000	-5.221088000	3.567373000
6	3.662577000	-5.394794000	3.815903000
6	3.307463000	-6.029769000	5.151671000
6	3.655813000	-5.165040000	6.353599000
6	3.092239000	-5.672674000	7.672793000
6	3.664531000	-7.007844000	8.118771000
6	3.115819000	-7.507445000	9.447562000
6	3.506786000	-6.656021000	10.642592000
1	4.814561000	0.097247000	-1.865618000
1	4.365712000	1.559889000	-0.989194000
1	7.237344000	1.014146000	-1.872119000
1	6.656735000	2.298095000	-0.825604000
1	5.766653000	0.429089000	0.716489000
1	7.482121000	0.740678000	0.689637000
1	6.183289000	-1.465705000	-0.961923000
1	7.897052000	-1.189946000	-0.732156000
1	7.585079000	-1.588215000	1.735552000
1	5.884946000	-1.861805000	1.446638000
1	6.563774000	-3.796590000	-0.094074000
1	8.211656000	-3.541361000	0.431444000
1	7.394932000	-3.866854000	2.834907000
1	7.380399000	-5.291341000	1.819752000
1	4.981880000	-5.083995000	1.431690000
1	4.923562000	-3.551325000	2.267180000
1	2.870434000	2.326792000	-2.500908000
1	3.081889000	0.762594000	-3.323187000
1	3.221614000	2.279648000	-4.248570000
1	5.244455000	0.130874000	-4.149697000
1	6.644666000	1.207389000	-3.914666000
1	5.346228000	1.677090000	-5.029423000
1	6.359890000	3.308983000	-2.825165000
1	4.977380000	3.756913000	-3.843508000
1	4.800492000	3.727409000	-2.070460000
1	5.640468000	-6.200129000	3.579613000
1	5.600045000	-4.642557000	4.378330000
1	3.177085000	-4.416598000	3.748516000
1	3.241453000	-6.000163000	3.008477000

1	3.800960000	-7.001797000	5.229338000
1	2.232234000	-6.231484000	5.170249000
1	3.275994000	-4.155137000	6.174075000
1	4.740802000	-5.070537000	6.445343000
1	3.280401000	-4.921014000	8.442000000
1	2.003782000	-5.760014000	7.589814000
1	4.754406000	-6.925597000	8.187305000
1	3.462840000	-7.761138000	7.354856000
1	3.468982000	-8.528210000	9.610345000
1	2.025750000	-7.570093000	9.383930000
1	3.145122000	-7.096697000	11.571833000
1	3.096195000	-5.649002000	10.576644000
1	4.592108000	-6.567529000	10.717019000
5	1.312753000	0.275399000	1.456062000
6	1.493498000	-0.352717000	2.968318000
6	1.789100000	0.435375000	4.082284000
1	1.989789000	1.490641000	3.944090000
6	1.835649000	-0.077942000	5.370734000
1	2.068655000	0.576436000	6.202652000
6	1.583682000	-1.421922000	5.595489000
1	1.624573000	-1.831385000	6.596855000
6	1.271587000	-2.231030000	4.513696000
1	1.060645000	-3.282518000	4.668888000
6	1.224600000	-1.697757000	3.234982000
1	0.967075000	-2.356338000	2.413072000
6	-0.304657000	0.565900000	1.334720000
6	-0.875651000	1.679661000	1.957065000
1	-0.228348000	2.385881000	2.465367000
6	-2.240064000	1.920362000	1.957929000
1	-2.633396000	2.797123000	2.458456000
6	-3.102576000	1.038223000	1.324369000
1	-4.169969000	1.218760000	1.318715000
6	-2.573067000	-0.084420000	0.709639000
1	-3.230041000	-0.793647000	0.220123000
6	-1.203228000	-0.311240000	0.726437000
1	-0.826287000	-1.204821000	0.243909000
6	1.863299000	-0.741197000	0.282730000
6	1.421866000	-0.635024000	-1.039037000
1	0.648433000	0.085451000	-1.278921000
6	1.933498000	-1.415556000	-2.063891000
1	1.551258000	-1.302211000	-3.071436000
6	2.936303000	-2.337461000	-1.804311000
1	3.342279000	-2.950027000	-2.599172000
6	3.413338000	-2.453799000	-0.509276000
1	4.202941000	-3.159213000	-0.284097000
6	2.883129000	-1.668083000	0.504013000
1	3.280735000	-1.786319000	1.504814000
6	2.199039000	1.646732000	1.233839000
6	1.764571000	2.703049000	0.430865000
1	0.769653000	2.665979000	0.003161000
6	2.559104000	3.806738000	0.153795000

1	2.177835000	4.601471000	-0.476416000
6	3.839373000	3.893110000	0.677705000
1	4.464562000	4.752002000	0.467793000
6	4.308464000	2.854656000	1.465933000
1	5.311414000	2.894935000	1.872451000
6	3.499771000	1.760754000	1.729793000
1	3.899860000	0.961688000	2.343813000

Table S2. Isotropic polarizability and anisotropy of polarizability (in au) calculated for isolated cation and anions.

System	α_{iso}	γ
CTA^+	314.57	37.30
$(C_8\text{-}OSO_3)^-$	181.42	9.69
$(C_{10}\text{-}OSO_3)^-$	213.34	13.67
$(C_{12}\text{-}OSO_3)^-$	246.36	17.73
$B(Ph)_4^-$	428.87	7.06

References

(1) Roe, D. R.; Cheatham, T. E. I. PTRAJ and CPPTRAJ: Software for Processing and Analysis of Molecular Dynamics Trajectory Data. *J. Chem. Theory Comput.* **2013**, *9* (7), 3084–3095. https://doi.org/10.1021/ct400341p.

(2) Michaud-Agrawal, N.; Denning, E. J.; Woolf, T. B.; Beckstein, O. MDAnalysis: A Toolkit for the Analysis of Molecular Dynamics Simulations. *J. Comput. Chem.* **2011**, *32* (10), 2319–2327. https://doi.org/10.1002/jcc.21787.

(3) Shao, J.; Tanner, S. W.; Thompson, N.; Cheatham, T. E. Clustering Molecular Dynamics Trajectories: 1. Characterizing the Performance of Different Clustering Algorithms. *J. Chem. Theory Comput.* **2007**, *3* (6), 2312–2334. https://doi.org/10.1021/ct700119m.

(4) Pedregosa, F.; Varoquaux, G.; Gramfort, A.; Michel, V.; Thirion, B.; Grisel, O.; Blondel, M.; Prettenhofer, P.; Weiss, R.; Dubourg, V.; Vanderplas, J.; Passos, A.; Cournapeau, D.; Brucher, M.; Perrot, M.; Duchesnay, E.; Louppe, G. Scikit-Learn: Machine Learning in Python. *J. Mach. Learn. Res.* **2012**, *12*.
